# Acceptance and Commitment Therapy Among Informal Caregivers of People With Chronic Health Conditions: A Systematic Review and Meta-Analysis

**DOI:** 10.1001/jamanetworkopen.2023.46216

**Published:** 2023-12-05

**Authors:** Fen Ye, Jung Jae Lee, Dandan Xue, Doris Sau-fung Yu

**Affiliations:** 1 School of Nursing, LKS Faculty of Medicine, The University of Hong Kong, Hong Kong, China

## Abstract

**Importance:**

Although there is substantial evidence to suggest the health benefits of acceptance and commitment therapy (ACT) among informal caregivers of people with chronic health conditions, the great variation in intervention designs among published studies limits its application.

**Objectives:**

To identify intervention characteristics of ACT that are associated with improved psychological health and to assess the acceptability of ACT among informal caregivers.

**Data Sources:**

Seven English- and 3 Chinese-language databases without limits on publication dates, the reference lists of previous reviews, and gray literature were searched up to February 2023.

**Study Selection:**

Randomized clinical trials comparing the effect of ACT vs control groups on improving psychological health among informal caregivers.

**Data Extraction and Synthesis:**

Two reviewers independently screened searched records and extracted data from eligible studies. Random-effects meta-analysis and mixed-effects metaregression were performed. The Preferred Reporting Items for Systematic Reviews and Meta-Analyses guideline was followed.

**Main Outcomes and Measures:**

Psychological health outcomes (eg, depressive symptoms) measured by valid measurements and the acceptability of ACT based on identified parameters.

**Results:**

A total of 29 studies with 2010 participants, published between 2015 and 2023, were identified. ACT showed moderate to large effect sizes for improving psychological health at postintervention assessments (Hedges *g* range, −0.55 [95% CI, −0.98 to −0.12] to −1.14 [95% CI, −1.83 to −0.45]) and at 1-to-3–month and 4-to-6–month follow-ups (Hedges *g* range, −0.47 [95% CI, −0.69 to −0.25] to −1.29 [95% CI, −2.33 to −0.24]). Multivariable metaregression analysis regarding intervention characteristics found that ACT delivered in a mixed individual- and group-based format, face-to-face, or through more intervention sessions was associated with greater improvements for experiential avoidance (face-to-face: β = −1.170 [95% CI, −2.020 to −0.319]; number of sessions: β = −0.242 [95% CI, −0.353 to −0.130]), depressive symptoms (mixed delivery format: β = −2.583 [95% CI, −4.845 to −0.321]; face-to-face: β = −1.555 [95% CI, −3.002 to −0.108]), or anxiety symptoms (face-to-face: β = −1.241 [95% CI, −2.337 to −0.146]). In general, ACT had low attrition rates (11%), and participants’ adherence (51%-80%) and satisfactory ratings (72%-95%) lend support to its acceptability.

**Conclusions and Relevance:**

This systematic review and meta-analysis found that ACT was consistently associated with improvements in psychological health, supporting its application to improve informal care for chronic disease management. This review provides specific details on the design parameters of ACT for achieving greater efficacy.

## Key Points

**Question:**

Which intervention characteristics are associated with the efficacy of acceptance and commitment therapy (ACT) in improving the psychological health of informal caregivers of people with chronic health conditions, and what is the acceptability of ACT?

**Findings:**

This systematic review and meta-analysis of 29 studies with 2010 participants suggested that ACT with mixed delivery format, face-to-face modality, and more intervention sessions was associated with greater improvements in psychological health. ACT was acceptable according to attrition rate, adherence, and caregivers’ satisfaction.

**Meaning:**

In this study, ACT seemed to achieve better therapeutic outcomes when it was individualized by including more face-to-face encounters and when intensive interventions with a mixed delivery format to facilitate peer support were applied.

## Teaser

This systematic review and meta-analysis identifies intervention characteristics of ACT that are associated with improved psychological health and assesses the acceptability of ACT among informal caregivers.

## Introduction

The World Health Organization (WHO) reports that 349 million people are care dependent.^1^ This situation is largely due to rapid global aging, the high prevalence of noncommunicable diseases, and injury.^2^ Rising life expectancy and long-term care dependency place heavy loads on the current informal care system, which buffers the burden on formal services.^3,4,5^ Unprepared informal caregivers must cope with various competing roles and multiple stressors when taking care of patients throughout the deteriorating and debilitating chronic disease trajectory, leading to a higher psychological burden than formal caregivers and noncaregivers.^6,7,8,9^ Meta-analyses have consistently reported that the prevalence of depressive and anxiety symptoms in this cohort is as high as 21% to 57%,^10,11,12^ leading to decreased care quality,^13^ greater economic impacts,^4,14^ poor quality of life for both caregivers and care recipients,^15,16^ and even caregivers’ suicide.^17,18^ Moreover, the overwhelming and unavoidable caregiving burden can diminish caregivers’ self-awareness of their own needs and hinder help-seeking.^19,20,21^ Proactive support to promote the psychological wellness of caregivers is urgently needed to sustain the informal care resource.

Acceptance and commitment therapy (ACT), an evolving cognitive behavioral intervention that has consistently demonstrated robust efficacy in addressing psychological distress,^21,22,23^ targets psychological inflexibility (eg, experiential avoidance and lack of value clarity) to promote psychological well-being.^22^ Experiential avoidance of various private events, including psychological suffering, may inhibit life functioning and lead to the avoidance of difficult tasks (eg, caregiving role).^23^ Compared with conventional psychotherapy, ACT places greater emphasis on acceptance and mindfulness, rather than changing the cognitive content, to enhance caregivers’ capacity and flexibility to adapt to long-term committed actions.^24,25,26,27,28^ A previous meta-analysis reported that ACT showed superior effects to traditional cognitive behavioral therapy for transdiagnostic conditions (*g* = 0.40).^28^ Furthermore, the American Psychological Association has recognized ACT as an evidence-based therapy to promote psychological (eg, depressive symptoms) and physical (eg, chronic pain) health.^29^

Two previous meta-analyses involving randomized clinical trials (RCTs) and quasi-experimental designs have indicated the beneficial effects of ACT on psychological health and quality of life among informal caregivers.^24,25^ Despite such substantial evidence, the clinical application of ACT in caregiving supportive practice is limited by wide variations in its design in empirical tests. As such, the overall aim of this review was to identify the crucial intervention characteristics that contributed to the success of ACT and users’ acceptance of the intervention. The evidence is urgently needed to promote the adoption of ACT in clinical practice.

## Methods

This review followed updated guidance of the Cochrane Handbook for Systematic Reviews of Intervention^30^ and Preferred Reporting Items for Systematic Reviews and Meta-Analyses (PRISMA) 2020 guidelines.^31^ Study protocol was registered a priori on the PROSPERO (CRD42022352783).

### Eligibility Criteria

This review included RCTs that examined the effects of ACT on improving psychological health among informal caregivers, including family members, friends, or neighbors. Eligible care recipients should have any chronic health condition (eg, function disabilities) that requires particular attention,^32^ regardless of when it was initially diagnosed or what stage it was in. Studies must report psychological health outcomes of informal caregivers, including psychological flexibility (eg, experiential avoidance) and other psychological health outcomes (eg, depressive symptoms) using any validated measurement. A detailed description is available in eMethods 1 in Supplement 1.

### Identification, Selection of Studies, and Data Extraction

Paired reviewers (F.Y. and D.D.X.) systematically searched for RCTs in PubMed, Embase, PsycInfo, British Nursing Index, CINAHLPlus, Web of Science, Cochrane Library, and 3 Chinese databases (China National Knowledge Infrastructure, Wanfang, and Weipu) without limits on publication dates (updated in February 2023) and with language restrictions to English or Chinese. Search strategies are available in eTable 1 in Supplement 1. The reference lists of published and topic-related reviews were manually screened. Additionally, gray literature on the official website of the Association for Contextual Behavioral Science was inspected. Records were screened and selected by the paired reviewers, with discrepancies resolved by consensus among 2 reviewers (eMethods 2 in Supplement 1). Paired reviewers extracted and coded various study-level data using 2 data forms with afterward cross-verification, including study design, participant characteristics, intervention characteristics, psychological health outcomes at the postintervention assessment and longest postintervention follow-up, and parameters reflecting acceptability. Further details are available in eMethods 3 in Supplement 1.

### Quality Assessment

Paired reviewers independently assessed the quality of included studies using the Cochrane Collaboration’s Risk of Bias Tool for RCTs.^33^ The quality of intervention implementation was assessed using the following criteria outlined by Chambless and Hollon^34^: (1) treatment protocol (either a published protocol or a protocol especially developed for informal caregivers), (2) facilitator training, and (3) checking integrity by supervision, recording, or screening of the intervention or protocol adherence. Disagreements were settled through discussion among 2 reviewers.

### Statistical Analysis

All analyses comprised RCTs with available data and were performed with R Studio, version 2022.07.0 + 548 (R Project for Statistical Computing). Further explanations are available in eMethods 4 in Supplement 1. A narrative synthesis was carried out if evaluation outcomes or parameters of the acceptability could not be included in meta-analyses.

#### Effect Size Calculation and Sensitivity Analysis

The pooled pre-post effect size for psychological health outcome is reported as a standardized mean difference (SMD) with 95% CI after performing random-effects meta-analyses.^35,36^ Hedges *g* was used to adjust the SMD due to small sample sizes, and heterogeneity was indicated using *I*^2^ and Q statistics, with interpretations in accordance with the Cochrane Handbook.^30^ A 95% prediction interval (95% PI) was generated to show the effect size ranges within which future studies would fall.^37^ Publication bias was assessed visually and quantitatively using a funnel plot and Egger test, respectively.^38^ Post hoc sensitivity analyses were performed using the leave-one-out method to test the robustness of the findings by excluding studies with the largest and smallest sample size and effect size.^39^

#### Subgroup and Metaregression Analyses

When at least 10 RCTs were identified for specific psychological health outcomes at postintervention and follow-up assessments,^30^ subgroup analyses and metaregression were performed to explore heterogeneity and examine how intervention characteristics were associated with the outcomes. Subgroup analyses for prespecified categorical moderators were conducted using random-effects models. Potential continuous moderators were explored through univariable metaregression analysis using the mixed-effects model with restricted maximum likelihood estimation. To prevent overfitting and ensure a stable model, intervention-level characteristics were included in the primary multivariable metaregression analysis. Sensitivity analyses that incorporated all study-level characteristics into multivariable metaregression models were also conducted. The multivariable metaregressions were performed using the forward selection method, adding factors with *P* < .10, starting with the factors the smallest *P* value derived from the results of univariable analyses.^40^ Multicollinearity was checked and controlled using the variance inflation factor (VIF < 2.5). To ensure robustness of findings and to adjust for multiple comparisons, thereby reducing the risk of type I error, the Holm-Bonferroni method was applied. Additionally, *P* values using the Higgins and Thompson permutation test method while accounting for multiplicity adjustment (10 000 permutations) were calculated.^41^

#### Meta-Analysis (Metaprop) for Attrition Rate

Attrition rate as an indicator of acceptability was defined as the percentage of informal caregivers who had not completed the postintervention assessment after being randomized to different groups. Estimated attrition rates with 95% CIs and PIs were pooled using random-effects meta-analyses with the generalized linear mixed model (GLMM).^42^ GLMM, as a 1-step approach of combining proportions and fully accounting for within-study variances, is particularly critical for limited sample sizes, zero counts, and result interpretation.^43^ This review set a 2-sided alpha at .05 to determine statistical significance.

## Results

### Selection, Study Characteristics, and Quality of Included Studies

**Figure 1.**
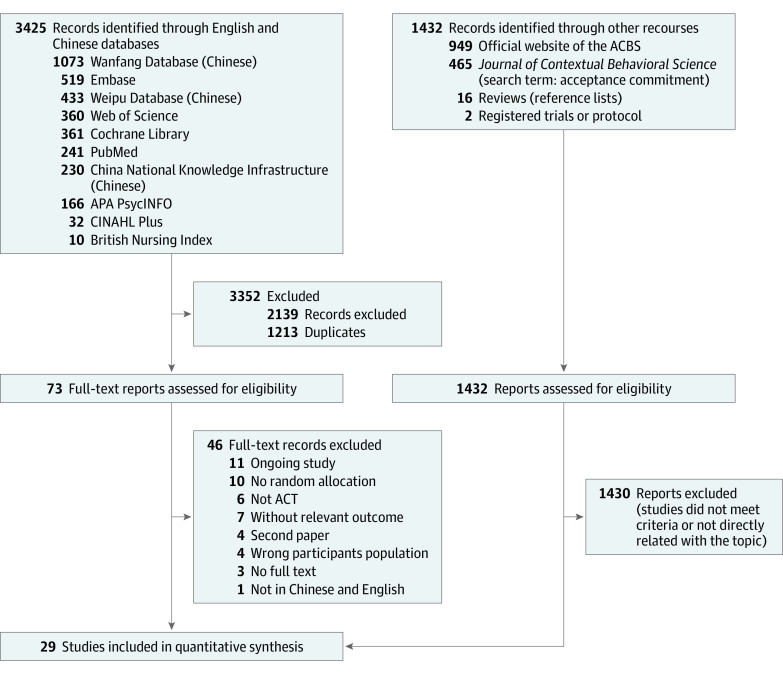
Flow Diagram of Identification, Screening, and Selection Processes

The systematic selection procedures (Figure 1) resulted in 29 eligible RCTs conducted in 11 countries (eFigure 1 in Supplement 1),^44,45,46,47,48,49,50,51,52,53,54,55,56,57,58,59,60,61,62,63,64,65,66,67,68,69,70,71,72^ comprising 2010 participants. The median sample size was 59 (range, 18-203), the mean age of participants was 46.4 years (range, 30.5-62.0 years), and the mean percentage of women was 77% (range, 38%-100%).

The main facilitators of ACT included trained professionals (eg, psychiatrist, psychologist, counselor, nurse, and social worker; 16 studies [55%])^44,46,47,48,50,53,54,57,60,62,64,65,66,67,68,71^ and trained students (eg, psychological and nursing students; 6 studies [21%]),^49,55,58,61,69,70^ whereas the other studies (7 [24%])^45,51,52,56,59,63,72^ adopted a self-help approach. Individual-based (16 studies [55%]),^45,46,47,49,51,52,54,56,57,59,60,63,65,68,70,72^ group-based (10 studies [34%]),^44,48,53,55,61,62,64,66,67,69^ and mixed (a combination of individual- and group-based delivery; 3 studies [10%])^50,58,71^ formats were used to deliver ACT. Most studies (22 [76%])^44,46,47,48,49,52,53,55,57,58,60,61,62,64,65,66,67,68,69,70,71,72^ used a face-to-face modality, while the others used telephone calls, online platform, and email. Approximately 55% of included studies (16 studies)^44,46,47,50,53,54,55,57,58,61,65,68,69,70,71,72^ used an active control group. The median intervention duration, number of intervention sessions, and time of therapeutic encounter were 8 weeks (range, 1-24 weeks), 6 sessions (range, 1-24 sessions), and 300 minutes (range, 0-2160 minutes), respectively. Further details are available in eResults 1 and 2 and eTables 2, 3, and 4 in Supplement 1.

Six of the RCTs had a low risk of bias,^50,51,53,56,62,65^ 16 had some concerns,^44,46,49,54,55,57,58,59,60,63,64,66,67,68,69,72^ and 7 had a high risk of bias.^45,47,48,52,61,70,71^ The risk of bias was mainly due to minimal baseline differences in the participants’ characteristics, uncertainty about concealment, the use of an inappropriate analysis method without dealing with missing data, the unavailability of data for almost all participants, and the lack of a published protocol or trial registration (eFigure 2 in Supplement 1).

### Overall Efficacy and Sensitivity Analysis

Table 1 presents the association of interventions with improved psychological health outcomes (eg, psychological flexibility). eFigures 3 to 9 in Supplement 1 present the forest plots for the meta-analyses at postintervention and follow-up assessments. The included studies found small to large effect sizes for the efficacy of ACT in improving psychological health outcomes at the postintervention assessment (Hedges *g* range, −0.55 [95% CI, −0.98 to −0.12] to −1.14 [95% CI, −1.83 to −0.45]) and at 1-to-3–month and 4-to-6–month follow-up assessments (Hedges *g* range, −0.47 [95% CI, −0.69 to −0.25] to −1.29 [95% CI, −2.33 to −0.24]), with low to considerable heterogeneity (*I^2^* = 0%-95%). However, no significant effect sizes were found for mindfulness (postintervention assessment) or value-based living (follow-ups). eFigures 10 and 11 in Supplement 1 present the funnel plots. Egger tests showed no publication bias for outcomes with 10 studies or more studies. Sensitivity analyses yielded similar results and identified the influential studies (eTable 5 in Supplement 1). After eliminating each main outlier,^44,45,46,47,48^ heterogeneity decreased, but significant results remained for value-based living, mindfulness, cognitive fusion, and stress symptoms (Hedges *g *range, −0.34 [95% CI, −0.49 to −0.19] to 1.23 [95% CI, 0.79 to 1.67]; *I^2^ =* 0%-44%).

**Table 1.** Effect Sizes of ACT for Psychological Flexibility and Other Psychological Health Outcomes Among Informal Caregivers

Outcomes	Studies, No.	Sample size, No.	SMD (95% CI) [95% PI]	*P* value	Heterogeneity			Publication bias (Eggers test)	
					*I*^2^, %	Q	*P* value	β (SE)	*P* value
**Psychological flexibility**									
Experiential avoidance									
Postintervention	23	1422	−1.04 (−1.65 to −0.44) [−4.19 to 2.10]	<.001	90	243.05	<.001	−1.96 (1.71)	.26
Follow-up^a^	15	979	−1.17 (−1.85 to −0.49) [−4.11 to 1.78]	<.001	89	139.58	<.001	−0.87 (2.04)	.68
Value-based living									
Postintervention	9	305	0.98 (0.25 to 1.71) [−1.65 to 3.60]	.01	82	49.78	<.001	2.68 (1.96)	.21
Follow-up^a^	5	159	0.27 (−0.29 to 0.84) [−1.20 to 1.95]	.34	60	12.52	.03	4.33 (1.15)	.02
Mindfulness									
Postintervention	7	330	1.19 (−0.23 to 2.61) [−3.99 to 6.37]	.10	88	58.29	<.001	3.41 (2.20)	.17
Follow-up^a^	3	125	0.90 (0.30 to 1.51) [−1.30 to 3.11]	.003	44	5.34	.15	−0.74 (2.04)	.75
Cognitive fusion									
Postintervention	6	264	−0.61 (−1.06 to −0.15) [−2.00 to 0.79]	.01	70	16.41	.01	−0.31 (2.92)	.92
Follow-up^a^	3	116	−0.68 (−1.05 to −0.30) [−3.12 to 1.77]	<.001	0	1.14	.57	1.00 (1.76)	.67
**Other psychological health outcomes**									
Depressive symptoms									
Post-intervention	14	945	−1.14 (−1.83 to −0.45) [−4.00 to 1.72]	.001	93	198.96	<.001	−5.17 (3.00)	.11
Follow-up^a^	9	668	−1.29 (−2.33 to −0.24) [−5.19 to 2.62]	.02	95	163.96	<.001	−4.13 (4.25)	.36
Anxiety symptoms									
Post-intervention	13	885	−1.02 (−1.57 to −0.48) [−3.21 to 1.16]	<.001	90	123.18	<.001	−4.07 (2.66)	.15
Follow-up^a^	10	708	−1.09 (−1.75 to −0.43) [−3.54 to 1.35]	.001	90	87.02	<.001	−1.73 (2.91)	.57
Stress symptoms									
Post-intervention	18	1270	−0.55 (−0.98 to −0.12) [−2.54 to 1.44]	.01	82	103.73	<.001	−2.16 (1.49)	.17
Follow-up^a^	12	874	−0.85 (−1.62 to −0.08) [−3.95 to 2.25]	.03	88	97.26	<.001	−2.68 (1.79)	.16

Abbreviations: ACT, acceptance and commitment therapy; PI, prediction interval.

^a^ Follow-up assessments included 1-to-3–month and 4-to-6–month follow-up assessments.

### Subgroup and Metaregression Analyses

eTables 6 and 7 in Supplement 1 show the between-group differences in the efficacy of the interventions for psychological health outcomes (≥10 studies ), including experiential avoidance and depressive, anxiety, and stress symptoms at postintervention and follow-up assessments. The risk of bias, health context for caregiving (particularly among caregivers of children with chronic health conditions and adults with central nervous system [CNS] diseases), the number of applied ACT processes (involving 4-6 interconnected processes such as acceptance, cognitive defusion, being present, self-as-context, value, and committed action), delivery formats (individual-based, group-based, and mixed formats), a face-to-face modality, and self-help were significantly associated with the effect sizes. Univariable metaregression (eTable 8 in Supplement 1) found significant associations between experiential avoidance, anxiety symptoms, or stress symptoms and factors such as the recruitment of younger caregivers, longer intervention duration, increased number of intervention sessions, longer time of therapeutic encounter, or lower attrition rate.

Multivariable metaregression analysis results regarding intervention characteristics are reported in Table 2. Statistically significant associations were found between the effect size for experiential avoidance at the postintervention assessment and a face-to-face modality (β = −1.170 [95% CI, −2.020 to −0.319]; *P* = .01) and every increase in the number of sessions (β = −0.242 [95% CI, −0.353 to −0.130]; *P* < .001). Greater effect size for depressive symptoms at the postintervention assessment was observed for a mixed individual- and group-based format (β = −2.583 [95% CI, −4.845 to −0.321]; *P* = .03) and face-to-face modality (β = −1.555 [95% CI, −3.002 to −0.108]; *P* = .04). A greater effect size of ACT on anxiety symptoms at the postintervention assessment was found for face-to-face modality (β = −1.241 [95% CI, −2.337 to −0.146]; *P* = .03).

**Table 2.** Summary of Multivariable Metaregression Analyses

Model	β (SE) [95% CI]	*P* value	*P* value		QM	*P* value	*I*^2^ residual, %
			Adjusted^a^	Permutation test^b^			
**Models regarding intervention characteristics**							
Experiential avoidance (post-intervention)							
Face-to-face	−1.170 (0.434) [−2.020 to −0.319]	.01	.01	.01	25.60	<.001	91
No. of sessions	−0.242 (0.057) [−0.353 to −0.130]	<.001	<.001	.004			
Experiential avoidance (follow-up)							
Face-to-face	−1.182 (0.643) [−2.442 to 0.078]	.07	.13	.04	3.38	.07	94
Depressive symptoms (postintervention)							
Delivery format							
Individual-based	−1.234 (0.683) [−2.572 to 0.105]	.07	.14	.09	9.52	.02	92
Mixed	−2.583 (1.154) [−4.845 to −0.321]	.03	.10	.08			
Face-to-face	−1.555 (0.738) [−3.002 to −0.108]	.04	.11	.049			
Anxiety symptoms (postintervention)							
Face-to-face	−1.241 (0.559) [−2.337 to −0.146]	.03	.05	.04	4.93	.03	90
**Models regarding study, participant, and intervention characteristics**							
Experiential avoidance (post-intervention)							
Risk of bias					36.31	<.001	89
Low	1.268 (0.593) [0.106 to 2.429]	.03	.09	.04			
Some concern	1.219 (0.562) [0.118 to 2.319]	.03	.09	.04			
No. of sessions	−0.239 (0.055) [−0.348 to −0.131]	<.001	.001	.004			
Attrition rate	4.500 (1.364) [1.826 to 7.174)	.001	.004	.003			
Experiential avoidance (follow-up)							
Face-to-face	−0.613 (0.223) [−1.050 to −0.176]	.01	.03	.04	38.48	<.001	13
No. of ACT processes							
6	−0.065 (0.400) [−0.849 to 0.718]	.87	.87	.87			
5	−0.970 (0.379) [−1.712 to −0.227]	.01	.03	.04			
Mean age	0.048 (0.017) [0.014 to 0.083]	.01	.03	.04			
No. of sessions	−0.168 (0.055) [−0.275 to −0.061]	.002	.01	.03			
Depressive symptoms (postintervention)							
Risk of bias					28.88	<.001	84
Low	2.475 (0.783) [0.940 to 4.010]	.002	.01	.02			
Some concern	1.837 (0.679) [0.506 to 3.167]	.001	.04	.03			
Delivery format							
Individual-based	−0.934 (0.544) [−2.000 to 0.131]	.09	.29	.12			
Mixed	−2.255 (0.892) [−4.005 to −0.506]	.01	.06	.08			
Face-to-face	−1.170 (0.679) [−2.500 to 0.161]	.08	.29	.14			
Self-help	1.227 (0.685) [−0.115 to 2.570]	.07	.29	.12			
Anxiety symptoms (postintervention)							
Risk of bias					15.73	.001	82
Low	1.772 (0.673) [0.453 to 3.091]	.001	.03	.03			
Some concern	0.625 (0.553) [−0.459 to 1.709]	.26	.52	.29			
Face-to-face	−1.723 (0.476) [−2.655 to −0.790]	<.001	.001	.01			
Anxiety symptoms (follow-up)							
6 ACT processes	0.787 (0.402) [−0.001 to 1.575]	.05	.05	.14	36.78	<.001	0
Mean age	0.078 (0.015) [0.049 to 0.108]	<.001	<.001	.01			
No. of sessions	−0.239 (0.060) [−0.357 to −0.121]	<.001	<.001	.03			
Stress symptoms (postintervention)							
Attrition rate	3.106 (1.390) [0.383 to 5.829]	.03	.03	.05	5.00	.03	90
Stress symptoms (follow-up)							
Mean age	0.033 (0.011) [0.012 to 0.053]	.002	.004	<.001	9.57	.01	0
Self-help	−0.359 (0.187) [−0.727 to 0.008]	.06	.06	.11			

Abbreviation: ACT, acceptance and commitment therapy.

^a^ Holm-Bonferroni method for adjusting *P* value.

^b^ Higgins and Thompson permutation test (10 000 iterations).

### Acceptability of ACT

**Figure 2.**
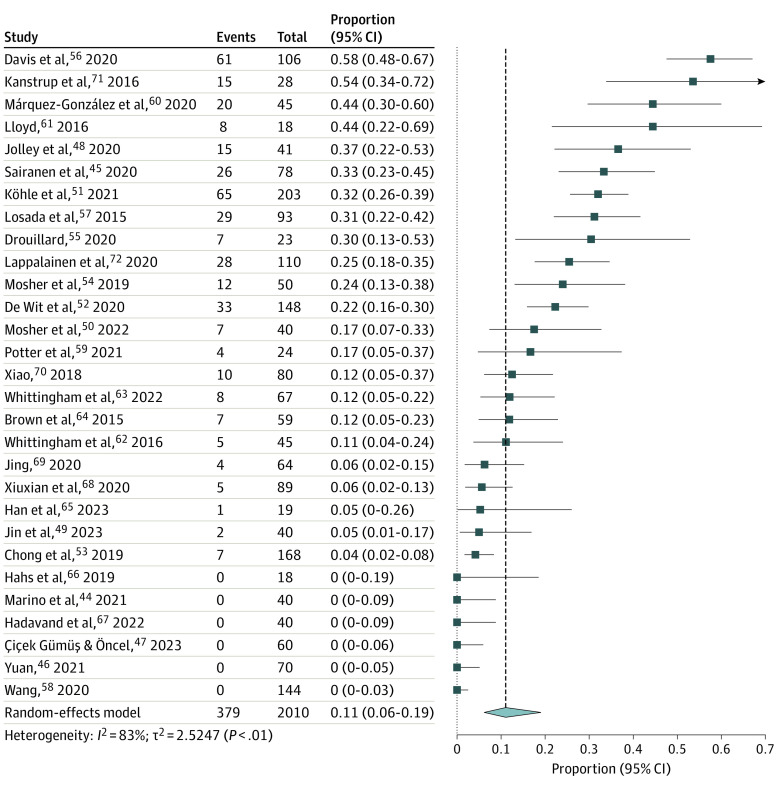
Meta-Analysis of Estimated Attrition Rates at the Postintervention Assessment

The pooled attrition rate of all RCTs, as presented in Figure 2, was 11% (95% CI, 6%-19%; *I^2^ =* 83%). Adherence to all intervention sessions (median, 76%; range, 51%-80%) was reported in 8 studies.^49,50,51,52,53,54,60,72^ Of the 10 studies that provided participants’ feedback,^45,48,49,50,51,52,55,56,57,72^ 4 studies^49,50,51,72^ included self-reported satisfaction rates (72%-95%). Participants reported that ACT had beneficial effects on caregiving, improved psychological health, and provided helpful intervention content. They also provided feedback on the limitations and made suggestions for improving existing interventions (Table 3). Those participants thought ACT was unhelpful mainly because “it is not for me*.”*^56^

**Table 3.** Narrative Summary of Participants’ Feedback on the Intervention

Theme	Details
Impact on caregiving	Sharing experiences and connecting with caregivers^48,55^ Realizing that self-care is essential for caregiving^48^ Allowing greater space and personal time for caregivers^48^ Realizing that others can help^48^ Having a holistic understanding of the care recipient and their disease^55^
Psychological health	Relaxing^48^ Reducing worry, anxiety, guilty, and stress^48^ Facing and dealing with problems^48^ Solving problems effectively^48^ Changing the way of reacting or looking at things and responding^48^ Being positive and hopeful^55^
Intervention content	Good quality^51^ Telephone sessions for blended interventions, facilitators, online interventions, and contents (eg, number and length of sessions, topics, experiential exercises, or information provided) are helpful^48,50,52,55,56^ Identifying caregivers’ value^48^ Favoring mindfulness and values sessions^55^ Matching the caregivers’ situation^52^ Useful for daily life^48^ Willing to participate in another comparable intervention^48^ Willing to recommend interventions to other caregivers^48,51,56,72^
Limitation or suggestion	Difficulties with the language when reading the text^59^ Difficulties with applying the concepts presented in the text^59^ Difficulties with ACT exercises^57^ Challenges with the text engagement and unsuitable content for caregivers^56,59^ Improving peer contact and mindfulness exercises for the online intervention^52^ Preferring the condensed version of the reading texts^59^ Giving enough time for reading the text and providing brief content^59^ Incorporating useful examples designed for caregivers^59^ Calls or email conversations help motivate caregivers to complete text reading^59^ A clear orientation is important for engagement^59^ Need more therapeutic input for text reading^59^ Need more interactive components for group-based intervention^55^

Abbreviation: ACT, acceptance and commitment therapy.

## Discussion

This comprehensive review of RCTs identified associations between ACT intervention characteristics accounting for other study-level confounders and its efficacy in improving the psychological health of informal caregivers. Extant evidence derived from 29 RCTs revealed that ACT was associated with sustained improvements in experiential avoidance, cognitive fusion, depressive symptoms, anxiety symptoms, and stress symptoms persisting 1.5 to 6 months after the intervention. Sensitivity analyses and undetected publication bias showed the robustness of the evidence obtained from these results. This review highlights that intervention characteristics (eg, delivery format, face-to-face modality, and the number of sessions) and study and participant characteristics (eg, risk of bias, caregivers’ age, health context for caregiving, and attrition rate) are relevant to the efficacy of ACT, which is a prospective and viable psychological therapy that is well-accepted by informal caregivers.

### Overall Efficacy

Converging with the substantial evidence for the therapeutic effects of ACT on psychological health outcomes among informal caregivers,^24,25^ this review identified moderate to large effect sizes of ACT on depressive, anxiety, and stress symptoms by adding 19 more RCTs than the number included in the prior reviews of which we are aware. This finding is comparable with the effect sizes of general medicines and psychiatric drugs reported in a frequently cited meta-analyses overview of psychological health,^73^ even though the longer-term effect of ACT beyond 6 months is yet to be established. This result is encouraging, as 75% of people with psychological distress prefer nonpharmacological treatment with minimal adverse effects.^74,75,76^ In addition, the current study identified positive effects of ACT on psychological flexibility of caregivers, including experiential avoidance, value-based living, mindfulness, and cognitive fusion. These outcomes suggest that ACT may cultivate psychological flexibility by improving the acceptance of emotions, clarifying a values-oriented life, promoting living in the present moment, distancing from negative feelings, and eventually, mediating the effects of the intervention on depressive, anxiety, and stress symptoms. However, ACT did not demonstrate a longer-term effect on value-based living, defined as the continuous embodiment and expression of chosen purposeful and meaningful action. As this construct is more about the existential aspect of being a caregiver, the stress-coping paradigm of the reviewed ACT may imply the inadequacy of the intervention content in fostering the positive aspects of caregiving (PAC), such as a sense of goal accomplishment, enhanced family dynamics, and finding meaning in life. A review on PAC indeed reported its role in improving the existential well-being of caregivers.^77^

### Intervention Characteristics

The reviewed studies showed that using an in-person method to deliver ACT was associated with higher ACTs’ efficacy than non–face-to-face modalities. The benefit may imply the increased relevance of an in-person encounter to optimize the caregivers’ engagement through therapeutic counseling techniques such as confrontation, reflection, and behavioral activation.^57^ The physical context also allows for sincere therapeutic interaction, enabling sensitive counseling and feedbacks, empathetic care, and partnership relationships to enrich the implementation of ACT. However, caregivers’ high role demands may limit applicability of ACT in a face-to-face modality due to accessibility issues.^25,78,79^ Although in-person interventions showed promising outcomes, further studies are needed to expand the evidence base for innovative interventions addressing accessibility obstacles, such as blending remote and face-to-face modalities.

This review identified a significant association of a mixed delivery format with ACT’s efficacy. This format may underline the crucial interplay between personal introspection and group interaction, connecting caregivers’ personal psychological processes to their engagement with external realities.^80^ Incorporating a group format facilitates shared experience, peer learning and support, and collective commitment to change.^81^ Furthermore, this review highlighted that ACT efficacy was associated with higher intensity (eg, increased number of sessions) and face-to-face modality in ACT delivery. This suggests that greater exposure to ACT principles and practices can yield more significant results. This aligns with the narrative synthesis results of caregivers’ feedback, which showed that they have difficulties with certain ACT aspects (eg, experiential exercise), emphasizing the necessity for sufficient intervention exposure.

Although the limited coverage of different health professionals among the reviewed studies did not allow an investigation of the effect of interveners’ professional backgrounds on outcomes, individual studies revealed that ACT can be delivered effectively by trained health professionals (eg, nurses) and students (eg, psychological and nursing students) as well as by a self-help approach. Additionally, peer-led interventions, leveraging lived experiences and empathy, are emerging as a promising approach for delivering ACT.^48^ These can also help diminish associated stigma. However, ensuring quality through adequate training and supervision is critical when using such task-shifting approaches. Health promoters with different backgrounds may effectively scale up ACT interventions to improve the psychological health of caregivers, especially in low- and middle-income countries with a scarcity of qualified psychologists.^82^

### Study and Participant Characteristics

Univariable analysis found that ACT’s efficacy significantly varied by caregivers’ age and health context for caregiving. Younger informal caregivers in particular benefitted from ACT, indicating that they may be more responsive to the intervention because they have a higher education level and are more receptive to learning new knowledge.^83^ This review also identified that caregiving context, characterized by the diverse experiences and unique demands associated with different diseases of care recipients^3^ were associated the efficacy of ACT. Particularly, caregivers of children with chronic health conditions and adults with CNS diseases demonstrated greater benefits from ACT. This could potentially be due to their adaptability to ACT techniques, prolonged caregiving durations, and ACT’s role in enhancing psychological flexibility in the face of disease-specific challenges.

### Acceptability of ACT

Overall, ACT was acceptable among informal caregivers, with similar attrition rates for other psychotherapies (ranging from 17%-20%).^84^ As the attrition rate is significantly associated with efficacy of ACT for experiential avoidance and stress symptoms, it is important to examine how to optimize the program design to best fit the caregivers’ preferences. Among the reviewed studies, codesign, as an important approach that actively involved the end-users in meeting their needs for psychological services,^85^ has seldom been used to guide the ACT program development. This may be an important perspective to optimize ACT design and improve its acceptability among caregivers.

### Implications

Our findings suggest that ACT is acceptable and effective in the caregiving context. However, high-quality trials should expand the research base to include caregivers with more challenging encounters in role fulfillment and to investigate the longer-term efficacy of ACT and the underlying mechanism explaining the psychological benefits of ACT. The RCTs included in this review applied 4 to 6 psychological flexibility processes together, due to the interconnected nature of these processes.^22^ The combined application prevents the examination of single and different combinations of ACT processes. Future research should examine how each of the 6 psychological flexibility processes individually contributes to improving caregivers’ psychological health, and how these processes interact and influence each other on a session-by-session basis. This understanding can inform the development of tailored ACT interventions, optimizing the sequence or combination of processes for maximum therapeutic efficacy. Additionally, the current review indicated that nonspecialists could master the techniques of administering ACT (eg, through workshops and self-learning).^58^ Therefore, future studies could investigate effective enabling mechanisms for trainers to increase the availability of this important therapy for supporting psychological recovery and adaptation to challenging situations, which is feasible since no official certification is needed for an ACT intervener.

### Limitations

The findings of this review need to be interpreted in the light of several limitations. First, the effectiveness of ACT in improving caregivers’ psychological health is still questionable when compared with an active psychological intervention (eg, cognitive behavioral therapy). Second, the effective ACT intervention characteristics for low-income countries with heavy caregiver burdens and for other psychological flexibility processes (ie, cognitive defusion, mindfulness, self-as-context, and commitment action) remain unknown. Third, the effect sizes of some subgroups also rendered the results uncertain because of an insufficient number of studies and unexplained heterogeneity. Despite an extensive multivariable metaregression analysis considering all study-level characteristics to capture potential confounder interactions in the clinical setting, the limited number of included studies necessitates caution in result interpretation due to the potential risk of overfitting. Finally, there were inadequate descriptions of how dropouts and missing data were managed with a statement on whether per-protocol or intention-to-treat analyses were applied.

## Conclusions

This review synthesized the available empirical evidence base, and the findings suggest that ACT has beneficial effects on the psychological health of informal caregivers of people with chronic health conditions. In accordance with WHO-recommended task sharing, it is feasible and beneficial to engage nonpsychological specialists (eg, nurses, social workers, and students) in the delivery of ACT for informal caregivers. However, the existing evidence does not explain the underlying mechanisms. The lack of evidence regarding engagement experience emphasizes the need for a vigorous process evaluation. Effective population-level psychotherapy is gaining prominence, and this review should serve as further impetus for the widespread adoption of effective ACT for informal caregivers to help them manage various forms of psychological distress.

## Notes

Supplement 1. **eMethods 1.** Eligibility Criteria **eMethods 2.** Study Selection **eMethods 3.** Data Extraction and Acquisition **eMethods 4.** Statistical Analysis **eResults 1.** Characteristics of Study Design and Participants of Included Studies **eResults 2.** Interventions Characteristics of Included Studies **eTable 1.** Search Strategies for Each Database **eTable 2.** Characteristics of Study Design and Participants of Included Studies **eTable 3.** Interventions Characteristics of Included Studies **eTable 4.** Overview of Study Characteristics of Included Studies **eTable 5.** Sensitivity Analysis for Psychological Flexibility and Other Psychological Health Outcomes **eTable 6.** Subgroup Analysis for Psychological Health Outcomes at Postintervention **eTable 7.** Subgroup Analysis for Psychological Health Outcomes at Follow-Up **eTable 8.** Summary of Univariable Metaregression Analyses **eFigure 1.** Map of Included Studies by Country **eFigure 2.** Risk of Bias of Included Studies **eFigure 3.** Forest Plots for Experimental Avoidance at Postintervention and 1- to 3-Month and 4- to 6-Month Follow-Ups **eFigure 4.** Forest Plots for Value-Based Living at Postintervention and 1- to 3-Month and 4- to 6-Month Follow-Ups **eFigure 5.** Forest Plots for Mindfulness at Postintervention and 1- to 3-Month and 4- to 6-Month Follow-Ups **eFigure 6.** Forest Plots for Cognitive Confusion at Postintervention and 1- to 3-Month and 4- to 6-Month Follow-Ups **eFigure 7.** Forest Plots for Depressive Symptoms at Postintervention and 1- to 3-Month and 4- to 6-Month Follow-Ups **eFigure 8.** Forest Plots for Anxiety Symptoms at Postintervention and 1- to 3-Month and 4- to 6-Month Follow-Ups **eFigure 9.** Forest Plots for Stress Symptoms at Postintervention and 1- to 3-Month and 4- to 6-Month Follow-Ups **eFigure 10.** Funnel Plots Depicting Standard Error of Effect Sizes for Psychological Flexibility **eFigure 11.** Funnel Plots Depicting Standard Error of Effect Sizes for Other Psychological Health Outcomes
eReferences.

Supplement 2.
Data Sharing Statement

## References

[1] World Health Organization. Integrated care for older people: guidelines on community-level interventions to manage declines in intrinsic capacity. Accessed May 1, 2023. https://www.who.int/publications/i/item/9789241550109 29608259

[2] Vos T, Lim SS, Abbafati C, ; GBD 2019 Diseases and Injuries Collaborators. Global burden of 369 diseases and injuries in 204 countries and territories, 1990-2019: a systematic analysis for the Global Burden of Disease Study 2019. Lancet. 2020;396(10258):1204-1222. doi:10.1016/S0140-6736(20)30925-9 33069326 PMC7567026

[3] Verbakel E, Metzelthin SF, Kempen GIJM. Caregiving to older adults: determinants of informal caregivers’ subjective well-being and formal and informal support as alleviating conditions. J Gerontol B Psychol Sci Soc Sci. 2018;73(6):1099-1111. 27130169 10.1093/geronb/gbw047

[4] Dunbar SB, Khavjou OA, Bakas T, ; American Heart Association. Projected costs of informal caregiving for cardiovascular disease: 2015 to 2035: a policy statement from the American Heart Association. Circulation. 2018;137(19):e558-e577. doi:10.1161/CIR.0000000000000570 29632217

[5] Broese van Groenou MI, De Boer A. Providing informal care in a changing society. Eur J Ageing. 2016;13(3):271-279. doi:10.1007/s10433-016-0370-7 27610055 PMC4992501

[6] Janson P, Willeke K, Zaibert L, . Mortality, morbidity and health-related outcomes in informal caregivers compared to non-caregivers: a systematic review. Int J Environ Res Public Health. 2022;19(10):5864. doi:10.3390/ijerph19105864 35627399 PMC9141545

[7] Wister A, Li L, Mitchell B, ; Canadian Longitudinal Study on Aging (CLSA) Team. Levels of depression and anxiety among informal caregivers during the COVID-19 pandemic: a study based on the Canadian Longitudinal Study on Aging. J Gerontol B Psychol Sci Soc Sci. 2022;77(9):1740-1757. doi:10.1093/geronb/gbac035 35150268 PMC8903401

[8] Stratmann M, Forsell Y, Möller J, Liang Y. Informal care and the impact on depression and anxiety among Swedish adults: a population-based cohort study. BMC Public Health. 2021;21(1):1263. doi:10.1186/s12889-021-11246-1 34187429 PMC8243546

[9] Zarit SH, Zarit JM. Family caregiving. In: Bensadon BA, ed. *Psychology and Geriatrics*. Academic Press; 2015:21-43.

[10] Sallim AB, Sayampanathan AA, Cuttilan A, Ho R. Prevalence of mental health disorders among caregivers of patients with Alzheimer disease. J Am Med Dir Assoc. 2015;16(12):1034-1041. doi:10.1016/j.jamda.2015.09.007 26593303

[11] Loh AZ, Tan JS, Zhang MW, Ho RC. The global prevalence of anxiety and depressive symptoms among caregivers of stroke survivors. J Am Med Dir Assoc. 2017;18(2):111-116. doi:10.1016/j.jamda.2016.08.014 27742585

[12] Cohn LN, Pechlivanoglou P, Lee Y, . Health outcomes of parents of children with chronic illness: a systematic review and meta-analysis. J Pediatr. 2020;218:166-177.e2. doi:10.1016/j.jpeds.2019.10.068 31916997

[13] Litzelman K, Kent EE, Mollica M, Rowland JH. How does caregiver well-being relate to perceived quality of care in patients with cancer? exploring associations and pathways. J Clin Oncol. 2016;34(29):3554-3561. doi:10.1200/JCO.2016.67.3434 27573657 PMC5074348

[14] Angioli R, Capriglione S, Aloisi A, . Economic impact among family caregivers of patients with advanced ovarian cancer. Int J Gynecol Cancer. 2015;25(8):1541-1546. doi:10.1097/IGC.0000000000000512 26270119

[15] Goren A, Gilloteau I, Lees M, DaCosta Dibonaventura M. Quantifying the burden of informal caregiving for patients with cancer in Europe. Support Care Cancer. 2014;22(6):1637-1646. doi:10.1007/s00520-014-2122-6 24496758

[16] Ademosu T, Ebuenyi I, Hoekstra RA, Prince M, Salisbury T. Burden, impact, and needs of caregivers of children living with mental health or neurodevelopmental conditions in low-income and middle-income countries: a scoping review. Lancet Psychiatry. 2021;8(10):919-928. doi:10.1016/S2215-0366(21)00207-8 34537102

[17] Chevance A, Ravaud P, Tomlinson A, . Identifying outcomes for depression that matter to patients, informal caregivers, and health-care professionals: qualitative content analysis of a large international online survey. Lancet Psychiatry. 2020;7(8):692-702. doi:10.1016/S2215-0366(20)30191-7 32711710

[18] Solimando L, Fasulo M, Cavallero S, . Suicide risk in caregivers of people with dementia: a systematic review and meta-analysis. Aging Clin Exp Res. 2022;34(10):2255-2260. doi:10.1007/s40520-022-02160-6 35696056 PMC9637612

[19] Lappalainen P, Keinonen K, Pakkala I, Lappalainen R, Nikander R. The role of thought suppression and psychological inflexibility in older family caregivers’ psychological symptoms and quality of life. J Contextual Behav Sci. 2021;20:129-136. doi:10.1016/j.jcbs.2021.04.005

[20] Ng CKM, Leung DKY, Cai X, Wong GHY. Perceived help-seeking difficulty, barriers, delay, and burden in carers of people with suspected dementia. Int J Environ Res Public Health. 2021;18(6):2956. doi:10.3390/ijerph18062956 33805808 PMC7999253

[21] Wang T, Molassiotis A, Chung BPM, Tan JY. Unmet care needs of advanced cancer patients and their informal caregivers: a systematic review. BMC Palliat Care. 2018;17(1):96. doi:10.1186/s12904-018-0346-9 30037346 PMC6057056

[22] Hayes SC, Luoma JB, Bond FW, Masuda A, Lillis J. Acceptance and commitment therapy: model, processes and outcomes. Behav Res Ther. 2006;44(1):1-25. doi:10.1016/j.brat.2005.06.006 16300724

[23] Hayes SC, Wilson KG. Acceptance and commitment therapy: altering the verbal support for experiential avoidance. Behav Anal. 1994;17(2):289-303. doi:10.1007/BF03392677 22478193 PMC2733476

[24] Han A, Yuen HK, Lee HY, Zhou X. Effects of acceptance and commitment therapy on process measures of family caregivers: A systematic review and meta-analysis. J Contextual Behav Sci. 2020;18:201-213. doi:10.1016/j.jcbs.2020.10.004

[25] Han A, Yuen HK, Jenkins J. Acceptance and commitment therapy for family caregivers: a systematic review and meta-analysis. J Health Psychol. 2021;26(1):82-102. doi:10.1177/1359105320941217 32659142

[26] Rickardsson N, Scotland J, Poveda B, Gillanders D. Caring for someone with an acquired brain injury: the role of psychological flexibility. J Contextual Behav Sci. 2022;23:151-164. doi:10.1016/j.jcbs.2022.01.005

[27] Jansen JE, Haahr UH, Lyse HG, Pedersen MB, Trauelsen AM, Simonsen E. Psychological flexibility as a buffer against caregiver distress in families with psychosis. Front Psychol. 2017;8:1625. doi:10.3389/fpsyg.2017.01625 29046649 PMC5632725

[28] Ruiz FJ. Acceptance and commitment therapy versus traditional cognitive behavioral therapy: a systematic review and meta-analysis of current empirical evidence. Int J Psychol Psychol Ther. 2012;12(3):333-357.

[29] Society of Clinical Psychology of the American Psychological Association. Psychological treatment. Accessed May 1, 2023. https://div12.org/treatments/

[30] Higgins J, Thomas J, Chandler J, , eds. Cochrane Handbook for Systematic Reviews of Interventions, Version 6.2 (Updated February 2021). Accessed May 1, 2023. https://training.cochrane.org/handbook

[31] Page MJ, McKenzie JE, Bossuyt PM, . The PRISMA 2020 statement: an updated guideline for reporting systematic reviews. Int J Surg. 2021;88:105906. doi:10.1016/j.ijsu.2021.105906 33789826

[32] Lawn S, Schoo A. Supporting self-management of chronic health conditions: common approaches. Patient Educ Couns. 2010;80(2):205-211. doi:10.1016/j.pec.2009.10.006 19931372

[33] Higgins JPT, Savović J, Page MJ, Elbers RG, Sterne JAC. Chapter 8: Assessing risk of bias in a randomized trial. Cochrane Handbook for Systematic Reviews of Interventions version 6.3 (updated February 2022). Accessed May 1, 2023. https://training.cochrane.org/handbook/current/chapter-08

[34] Chambless DL, Hollon SD. Defining empirically supported therapies. J Consult Clin Psychol. 1998;66(1):7-18. doi:10.1037/0022-006X.66.1.7 9489259

[35] Borenstein M, Hedges LV, Higgins JP, Rothstein HR. Introduction to Meta-Analysis. John Wiley & Sons; 2021. doi:10.1002/9781119558378

[36] Rabiee AR, Lean IJ, Stevenson MA, Socha MT. Effects of feeding organic trace minerals on milk production and reproductive performance in lactating dairy cows: a meta-analysis. J Dairy Sci. 2010;93(9):4239-4251. doi:10.3168/jds.2010-3058 20723697

[37] IntHout J, Ioannidis JP, Rovers MM, Goeman JJ. Plea for routinely presenting prediction intervals in meta-analysis. BMJ Open. 2016;6(7):e010247. doi:10.1136/bmjopen-2015-010247 27406637 PMC4947751

[38] Rothstein HR, Sutton AJ, Borenstein M. Publication bias in meta-analysis. In: Rothstein HR, Sutton AJ, Borenstein M, eds. *Publication Bias in Meta-Analysis: Prevention, Assessment and Adjustments*. Wiley. 2005:1-7.

[39] Copas J, Shi JQ. Meta-analysis, funnel plots and sensitivity analysis. Biostatistics. 2000;1(3):247-262. doi:10.1093/biostatistics/1.3.247 12933507

[40] Chowdhury MZI, Turin TC. Variable selection strategies and its importance in clinical prediction modelling. Fam Med Community Health. 2020;8(1):e000262. doi:10.1136/fmch-2019-000262 32148735 PMC7032893

[41] Higgins JP, Thompson SG. Controlling the risk of spurious findings from meta-regression. Stat Med. 2004;23(11):1663-1682. doi:10.1002/sim.1752 15160401

[42] Lin L, Xu C. Arcsine-based transformations for meta-analysis of proportions: pros, cons, and alternatives. Health Sci Rep. 2020;3(3):e178. doi:10.1002/hsr2.178 32728636 PMC7384291

[43] Lin L, Chu H. Meta-analysis of proportions using generalized linear mixed models. Epidemiology. 2020;31(5):713-717. doi:10.1097/EDE.0000000000001232 32657954 PMC7398826

[44] Marino F, Failla C, Chilà P, . The effect of acceptance and commitment therapy for improving psychological well-being in parents of individuals with autism spectrum disorders: a randomized controlled trial. Brain Sci. 2021;11(7):880. doi:10.3390/brainsci11070880 34209171 PMC8301771

[45] Sairanen E, Lappalainen R, Lappalainen P, . Effectiveness of a web-based acceptance and commitment therapy intervention for wellbeing of parents whose children have chronic conditions: a randomized controlled trial. J Contextual Behav Sci. 2019;13:94-102. doi:10.1016/j.jcbs.2019.07.004

[46] Yuan B. A Study on Psychological Intervention of Acceptance and Commitment Therapy for Parents of Children with Autism at First Diagnosis. Master’s thesis. Nanchang University; 2021. Accessed May 1, 2023. https://d.wanfangdata.com.cn/thesis/ChJUaGVzaXNOZXdTMjAyMzAxMTISCUQwMjQzNjk2NBoIOGN3YXN3NGM%3D

[47] Çiçek Gümüş E, Öncel S. Effects of acceptance and commitment therapy-based interventions on the mental states of parents with special needs children: Randomized controlled trial. Curr Psychol. 2023;42:19429-19442.

[48] Jolley S, Johns LC, O’Donoghue E, . Group acceptance and commitment therapy for patients and caregivers in psychosis services: feasibility of training and a preliminary randomized controlled evaluation. Br J Clin Psychol. 2020;59(4):524-551. doi:10.1111/bjc.12265 32944971

[49] Jin X, Li H, Chong YY, Mann KF, Yao W, Wong CL. Feasibility and preliminary effects of acceptance and commitment therapy on reducing psychological distress and improving the quality of life of the parents of children with cancer: a pilot randomised controlled trial. Psychooncology. 2023;32(1):165-169. doi:10.1002/pon.5941 35460318

[50] Mosher CE, Secinti E, Wu W, . Acceptance and commitment therapy for patient fatigue interference and caregiver burden in advanced gastrointestinal cancer: results of a pilot randomized trial. Palliat Med. 2022;36(7):1104-1117. doi:10.1177/02692163221099610 35637615 PMC9396957

[51] Köhle N, Drossaert CHC, Ten Klooster PM, . Web-based self-help intervention for partners of cancer patients based on acceptance and commitment therapy and self-compassion training: a randomized controlled trial with automated versus personal feedback. Support Care Cancer. 2021;29(9):5115-5125. doi:10.1007/s00520-021-06051-w 33608762 PMC8295082

[52] De Wit J, Beelen A, Drossaert CHC, . Blended psychosocial support for partners of patients with ALS and PMA: results of a randomized controlled trial. Amyotroph Lateral Scler Frontotemporal Degener. 2020;21(5-6):344-354. doi:10.1080/21678421.2020.1757114 32362155

[53] Chong YY, Mak YW, Leung SP, Lam SY, Loke AY. Acceptance and commitment therapy for parental management of childhood asthma: an RCT. Pediatrics. 2019;143(2):e20181723. doi:10.1542/peds.2018-1723 30659063

[54] Mosher CE, Secinti E, Hirsh AT, . Acceptance and commitment therapy for symptom interference in advanced lung cancer and caregiver distress: a pilot randomized trial. J Pain Symptom Manage. 2019;58(4):632-644. doi:10.1016/j.jpainsymman.2019.06.021 31255586 PMC6754796

[55] Drouillard BE. Supporting Treatment Selection in Parents of Children With Autism Spectrum Disorder: An Educational Workshop With Acceptance and Commitment Training. Dissertation. University of Windsor; 2020. Accessed May 1, 2023. https://www.proquest.com/docview/2322824738?pq-origsite=gscholar&fromopenview=true

[56] Davis EL, Deane FP, Lyons GC, Barclay GD, Bourne J, Connolly V. Feasibility randomised controlled trial of a self-help acceptance and commitment therapy intervention for grief and psychological distress in carers of palliative care patients. J Health Psychol. 2020;25(3):322-339. doi:10.1177/1359105317715091 28810477

[57] Losada A, Márquez-González M, Romero-Moreno R, . Cognitive-behavioral therapy (CBT) versus acceptance and commitment therapy (ACT) for dementia family caregivers with significant depressive symptoms: results of a randomized clinical trial. J Consult Clin Psychol. 2015;83(4):760-772. doi:10.1037/ccp0000028 26075381

[58] Wang Z. Application Study of Acceptance and Commitment Therapy in Psychological Intervention of Main Caregivers for Children With Cerebral Palsy. Master’s thesis. Anhui Medical University; 2020. Accessed May 1, 2023. https://d.wanfangdata.com.cn/thesis/ChJUaGVzaXNOZXdTMjAyMzAxMTISCUQwMjI3OTU0ORoIZXB5M25kazk%3D

[59] Potter KJ, Golijana-Moghaddam N, Evangelou N, et al. Self-help acceptance and commitment therapy for carers of people with multiple sclerosis: a feasibility randomized controlled trial. J Clin Psychol Med Settings. 2021;28(2):279-294. doi:10.1007/s10880-020-09711-x 32144616 PMC8192317

[60] Márquez-González M, Romero-Moreno R, Cabrera I, Olmos R, Pérez-Miguel A, Losada A. Tailored versus manualized interventions for dementia caregivers: the functional analysis-guided modular intervention. Psychol Aging. 2020;35(1):41-54. doi:10.1037/pag0000412 31985248

[61] Lloyd A. The Use of Acceptance and Commitment Therapy to Address Psychological Distress Experienced by Caregivers: A Randomized Controlled Feasibility Trial. Dissertation. University of Glasgow; 2016. Accessed November 2, 2023. https://theses.gla.ac.uk/7590/1/2016LloydDClinPsy.pdf

[62] Whittingham K, Sanders MR, McKinlay L, Boyd RN. Parenting intervention combined with acceptance and commitment therapy: a trial with families of children with cerebral palsy. J Pediatr Psychol. 2016;41(5):531-542. doi:10.1093/jpepsy/jsv118 26702629 PMC4888113

[63] Whittingham K, Sheffield J, Mak C, Wright A, Boyd RN. Parenting acceptance and commitment therapy: an RCT of an online course with families of children with CP. Behav Res Ther. 2022;155:104129. doi:10.1016/j.brat.2022.104129 35662680

[64] Brown FL, Whittingham K, Boyd RN, McKinlay L, Sofronoff K. Does Stepping Stones Triple P plus acceptance and commitment therapy improve parent, couple, and family adjustment following paediatric acquired brain injury? a randomised controlled trial. Behav Res Ther. 2015;73:58-66. doi:10.1016/j.brat.2015.07.001 26255172

[65] Han A, Yuen HK, Jenkins J. The feasibility and preliminary effects of a pilot randomized controlled trial: videoconferencing acceptance and commitment therapy in distressed family caregivers of people with dementia. J Health Psychol. 2023;28(6):554-567. doi:10.1177/13591053221141131 36591636 PMC10119897

[66] Hahs AD, Dixon MR, Paliliunas D. Randomized controlled trial of a brief acceptance and commitment training for parents of individuals diagnosed with autism spectrum disorders. J Contextual Behav Sci. 2019;12:154-159. doi:10.1016/j.jcbs.2018.03.002

[67] Hadavand M, Zanjani Z, Omidi A, Atoof F, Fakharian E. Acceptance and commitment: an intervention for improving family function and emotional problems in informal caregivers of people with severe traumatic brain injury: a randomized clinical trial. Arch Trauma Res. 2022;11(2):90-96. doi:10.4103/atr.atr_4_22

[68] Xiuxian X, Haiying H, Zhimin L. Effects of acceptance and commitment therapy on anxiety, depression, and posttraumatic growth of parents of children with leukemia. Chinese Gen Pract Nurs. 2020;18(25):3318-3320. doi:10.12104/j.issn.1674-4748.2020.25.015

[69] Jing X. Study of Acceptance and Commitment Therapy in Family Function of the First Episode Schizophrenia Patients. Master’s thesis. Nanchang University; 2020. Accessed May 1, 2023. https://d.wanfangdata.com.cn/thesis/ChJUaGVzaXNOZXdTMjAyMzAxMTISCUQwMjIwNDM4NxoIejVseXhxa3c%3D

[70] Xiao N. *A Study on the Application of Acceptance and Commitment Therapy in Psychological Intervention for Parents of Children With Leukemia*. Master’s thesis. University of South China; 2018. Accessed November 6, 2023. https://d.wanfangdata.com.cn/thesis/ChJUaGVzaXNOZXdTMjAyMzA5MDESCUQwMTY4ODQwNhoIdzhsa3c2M3I%3D

[71] Kanstrup M, Wicksell RK, Kemani M, Wiwe Lipsker C, Lekander M, Holmström L. A clinical pilot study of individual and group treatment for adolescents with chronic pain and their parents: effects of acceptance and commitment therapy on functioning. Children (Basel). 2016;3(4):30. doi:10.3390/children3040030 27854323 PMC5184805

[72] Lappalainen P, Pakkala I, Strömmer J, Sairanen E, Kaipainen K, Lappalainen R. Supporting parents of children with chronic conditions: a randomized controlled trial of web-based and self-help ACT interventions. Internet Interv. 2021;24:100382. doi:10.1016/j.invent.2021.100382 33816128 PMC8010620

[73] Leucht S, Hierl S, Kissling W, Dold M, Davis JM. Putting the efficacy of psychiatric and general medicine medication into perspective: review of meta-analyses. Br J Psychiatry. 2012;200(2):97-106. doi:10.1192/bjp.bp.111.096594 22297588

[74] Holmes EA, Ghaderi A, Harmer CJ, . The Lancet Psychiatry Commission on psychological treatments research in tomorrow’s science. Lancet Psychiatry. 2018;5(3):237-286. doi:10.1016/S2215-0366(17)30513-8 29482764

[75] Croatto G, Vancampfort D, Miola A, . The impact of pharmacological and non-pharmacological interventions on physical health outcomes in people with mood disorders across the lifespan: an umbrella review of the evidence from randomised controlled trials. Mol Psychiatry. 2023;28(1):369-390. doi:10.1038/s41380-022-01770-w 36138129 PMC9493151

[76] Muttoni S, Ardissino M, John C. Classical psychedelics for the treatment of depression and anxiety: a systematic review. J Affect Disord. 2019;258:11-24. doi:10.1016/j.jad.2019.07.076 31382100

[77] Yu DSF, Cheng ST, Wang J. Unravelling positive aspects of caregiving in dementia: an integrative review of research literature. Int J Nurs Stud. 2018;79:1-26. doi:10.1016/j.ijnurstu.2017.10.008 29128685

[78] Prince M, Brodaty H, Uwakwe R, . Strain and its correlates among carers of people with dementia in low-income and middle-income countries: a 10/66 Dementia Research Group population-based survey. Int J Geriatr Psychiatry. 2012;27(7):670-682. doi:10.1002/gps.2727 22460403 PMC3504977

[79] Bai Z, Luo S, Zhang L, Wu S, Chi I. Acceptance and commitment therapy (ACT) to reduce depression: a systematic review and meta-analysis. J Affect Disord. 2020;260:728-737. doi:10.1016/j.jad.2019.09.040 31563072

[80] Swiller HI. Alexithymia: treatment utilizing combined individual and group psychotherapy. Int J Group Psychother. 1988;38(1):47-61. doi:10.1080/00207284.1988.11491084 3350615

[81] Mallinckrodt B. Attachment, Social Competencies, Social Support, and Interpersonal Process in Psychotherapy. Psychother Res. 2010;10(3):239-266. doi:10.1093/ptr/10.3.239

[82] van Ginneken N, Tharyan P, Lewin S, . Non-specialist health worker interventions for the care of mental, neurological and substance-abuse disorders in low- and middle-income countries. Cochrane Database Syst Rev. 2013;(11):CD009149. doi:10.1002/14651858.CD009149.pub2 24249541

[83] Satre DD, Knight BG, David S. Cognitive-behavioral interventions with older adults: Integrating clinical and gerontological research. *Prof Psychol Res Pract*. 2006;37(5):489-498. doi:10.1037/0735-7028.37.5.489

[84] Swift JK, Greenberg RP. Premature discontinuation in adult psychotherapy: a meta-analysis. J Consult Clin Psychol. 2012;80(4):547-559. doi:10.1037/a0028226 22506792

[85] O’Brien J, Fossey E, Palmer VJ. A scoping review of the use of co-design methods with culturally and linguistically diverse communities to improve or adapt mental health services. Health Soc Care Community. 2021;29(1):1-17. doi:10.1111/hsc.13105 32686881

