# Supplementary material for: Acceptance and Commitment Therapy Among Informal Caregivers of People With Chronic Health Conditions: A Systematic Review and Meta-Analysis
Source: JAMA Netw Open. 2023 Dec 5;6(12):e2346216. doi: 10.1001/jamanetworkopen.2023.46216 (PMC10698615; doi:10.1001/jamanetworkopen.2023.46216)
Supplement: Supplement 1. — eMethods 1. Eligibility Criteria eMethods 2. Study Selection eMethods 3. Data Extraction and Acquisition eMethods 4. Statistical Analysis eResults 1. Characteristics of Study Design and Participants of Included Studies eResults 2. Interventions Characteristics of Included Studies eTable 1. Search Strategies for Each Database eTable 2. Characteristics of Study Design and Participants of Included Studies eTable 3. Interventions Characteristics of Included Studies eTable 4. Overview of Study Characteristics of Included Studies eTable 5. Sensitivity Analysis for Psychological Flexibility and Other Psychological Health Outcomes eTable 6. Subgroup Analysis for Psychological Health Outcomes at Postintervention eTable 7. Subgroup Analysis for Psychological Health Outcomes at Follow-Up eTable 8. Summary of Univariable Metaregression Analyses eFigure 1. Map of Included Studies by Country eFigure 2. Risk of Bias of Included Studies eFigure 3. Forest Plots for Experimental Avoidance at Postintervention and 1- to 3-Month and 4- to 6-Month Follow-Ups eFigure 4. Forest Plots for Value-Based Living at Postintervention and 1- to 3-Month and 4- to 6-Month Follow-Ups eFigure 5. Forest Plots for Mindfulness at Postintervention and 1- to 3-Month and 4- to 6-Month Follow-Ups eFigure 6. Forest Plots for Cognitive Confusion at Postintervention and 1- to 3-Month and 4- to 6-Month Follow-Ups eFigure 7. Forest Plots for Depressive Symptoms at Postintervention and 1- to 3-Month and 4- to 6-Month Follow-Ups eFigure 8. Forest Plots for Anxiety Symptoms at Postintervention and 1- to 3-Month and 4- to 6-Month Follow-Ups eFigure 9. Forest Plots for Stress Symptoms at Postintervention and 1- to 3-Month and 4- to 6-Month Follow-Ups eFigure 10. Funnel Plots Depicting Standard Error of Effect Sizes for Psychological Flexibility eFigure 11. Funnel Plots Depicting Standard Error of Effect Sizes for Other Psychological Health Outcomes eReferences. [file jamanetwopen-e2346216-s001.pdf]

Supplemental Online Content

Ye F, Lee JJ, Xue D, Yu DS. Acceptance and commitment therapy among informal caregivers of people with chronic health conditions. *JAMA Netw Open.* 2023;6(12):e2346216. doi:10.1001/jamanetworkopen.2023.46216

eMethods 1. Eligibility Criteria

eMethods 2. Study Selection

eMethods 3. Data Extraction and Acquisition

eMethods 4. Statistical Analysis

eResults 1. Characteristics of Study Design and Participants of Included Studies

eResults 2. Interventions Characteristics of Included Studies

eTable 1. Search Strategies for Each Database

eTable 2. Characteristics of Study Design and Participants of Included Studies

eTable 3. Interventions Characteristics of Included Studies

eTable 4. Overview of Study Characteristics of Included Studies

eTable 5. Sensitivity Analysis for Psychological Flexibility and Other Psychological Health Outcomes

eTable 6. Subgroup Analysis for Psychological Health Outcomes at Postintervention

eTable 7. Subgroup Analysis for Psychological Health Outcomes at Follow-Up

eTable 8. Summary of Univariable Metaregression Analyses

eFigure 1. Map of Included Studies by Country

eFigure 2. Risk of Bias of Included Studies

eFigure 3. Forest Plots for Experimental Avoidance at Postintervention and 1- to 3-Month and 4- to 6-Month Follow-Ups

eFigure 4. Forest Plots for Value-Based Living at Postintervention and 1- to 3-Month and 4- to 6-Month Follow-Ups

eFigure 5. Forest Plots for Mindfulness at Postintervention and 1- to 3-Month and 4- to 6-Month Follow-Ups

eFigure 6. Forest Plots for Cognitive Confusion at Postintervention and 1- to 3-Month and 4- to 6-Month Follow-Ups

eFigure 7. Forest Plots for Depressive Symptoms at Postintervention and 1- to 3-Month and 4- to 6-Month Follow-Ups

eFigure 8. Forest Plots for Anxiety Symptoms at Postintervention and 1- to 3-Month and 4- to 6-Month Follow-Ups

eFigure 9. Forest Plots for Stress Symptoms at Postintervention and 1- to 3-Month

© 2023 Ye F et al. *JAMA Network Open.*

and 4- to 6-Month Follow-Ups

eFigure 10. Funnel Plots Depicting Standard Error of Effect Sizes for Psychological Flexibility

eFigure 11. Funnel Plots Depicting Standard Error of Effect Sizes for Other Psychological Health Outcomes

eReferences.

This supplemental material has been provided by the authors to give readers additional information about their work.

eMethods 1. Eligibility Criteria

The study protocol, review, ongoing study, conference abstract, and editorial letter were excluded. If repetitive studies had the same data from the same registered study, reviewers included the complete report with the longest follow-up period. The exclusion criteria of participants were informal caregivers who were (1) diagnosed with psychiatric disorders (eg, bipolar disorder); (2) caring for care recipients in long-term facilities. Acceptance and commitment therapy (ACT) was implemented for informal caregivers or dyads and delivered in any format. Single- or multi-component interventions were eligible. ACT was applied through any number of the 6 processes and was conducted alone or as one main part of interventions. Studies that solely used the ACT for care recipients were excluded. The comparison group received either an active control (eg, a different tension of the same intervention, a different type of therapy) or inactive control (eg, usual care, no treatment, standard care, or waiting list). Usual care was defined as the ordinary participation in activities offered by informal caregivers.

eMethods 2. Study Selection

Searched records were downloaded into Endnote 20.2 for screening. Duplicates were removed automatically and manually after inputting all the studies. Paired reviewers (F.Y.) and (D.D.X.) independently reviewed all titles and abstracts to retrieve potentially eligible studies based on selection criteria. Any discrepancies were settled by mutual discussion for titles and abstracts screening among 2 reviewers. The full texts were checked to determine the study's inclusion if descriptions in the title and abstract were insufficient. The potentially included studies were reviewed with a full-text screen for final inclusion. The reasons for the final exclusion were listed in eFigure 1. Study authors were contacted if a full-text report or crucial data were inaccessible.

eMethods 3. Data Extraction and Acquisition

Study characteristics were extracted by referencing the Cochrane Review template,[1] including author, published year, country, study setting, research topic, health context for caregiving (ie, health conditions of the care recipients), sample size, target participants, age, gender, outcomes, evaluation points of the outcomes, and attrition rate. Intervention characteristics include protocol, facilitators, fidelity check, the adaptation of the ACT content, delivery format and modality, the number of ACT processes, additional education content, duration of the intervention, the number of sessions, the time of each session, time of therapeutic encounter (ie, facilitator-participant interaction and contact time through face-to-face or telephone call), materials, and control group.

eMethods 4. Statistical Analysis

Intervention groups applying ACT and control groups without applying ACT were selected for multiple-arms randomized clinical trials (RCTs) by splitting the "shared" control group into two half numbers of sample sizes with equal means and standard deviations (SD).[2] Unavailability SD was determined by sample size, standard error, and 95% confidence interval (CI).[2] Effect sizes were calculated through pre-post mean changes and an estimated correlation coefficient ($r = 0.7$) suggested by Rosenthal.[3,4] Hedges *g* was used with the interpretation of small (0.2-0.5), moderate (> 0.5), and large (> 0.8), based on Cohen's criteria and Cochrane guideline.[5,6] Since the outcomes of included studies were evaluated using various assessment measurements, participants and interventions varied in ways that would affect the outcomes, and detectable heterogeneity was identified in this review, random-effects models were selected for meta-analysis. Cochran's Q test, which was based on chi-square distribution, was performed to verify the total variance for choosing the random effects models and determined the heterogeneity if $P<.1$ (variation in effect estimated that is not random).[7] The heterogeneity variance τ^2 was calculated using the restricted maximum likelihood estimator.[8] This review will also use statistical I^2 tests to assess the percentage of the overall variation between trials that is attributable to heterogeneity rather than random, with the interpretation of might not be important (0% to 40%), moderate (30% to 60%), substantial (50% to 90%), and considerable (75% to 100%).[2] The inverse variance approach was chosen to calculate the weights of the meta-analysis.[2] The meta-regression coefficient (β) represented the predicted changes in the moderators' effect size.[7] Predefined intervention-level characteristics included intervention content, number of ACT processes, delivery format, face-to-face modality, self-help, active control, intervention duration, the number of sessions, and time of therapeutic encounter. Study and participant characteristics included Chinese study, setting, risk of bias, caregivers' age, health context for caregiving, sample size, and attrition rate.

© 2023 Ye F et al. *JAMA Network Open.*

eResults 1. Characteristics of Study Design and Participants of Included Studies

The 29 RCTs were conducted in 11 countries: Australia,[9-12] Canada,[13] China,[14-20] Finland,[21] Italy,[22] Netherlands,[23,24] Spain,[25,26] Sweden,[27,28] Turkey,[29,30] United Kingdom,[31-33] and United States of America.[34-37] Participants were derived from the community (*k* = 21)[10,12-14,17,18,21-29,31,32,34-37] and the hospital (*k* = 8).[9,11,15,16,19,20,30,33] Informal caregivers of four populations of care recipients were concerned, including children with chronic conditions (autism spectrum disorders, cerebral palsy, asthma, leukemia, acquired brain injury, cancer and chronic pain, and special needs; *k* = 16),[10-15,17-22,27-29,35] adults with central nervous system (CNS) diseases (dementia, multiple sclerosis, amyotrophic lateral sclerosis, progressive muscular atrophy, and acquired brain injury; *k* = 6),[24-26,31,33,37] adults with life-limiting illnesses (cancer and patients in palliative care; *k* = 4),[9,23,34,36] adults with psychotic disorders (psychosis and schizophrenia, *k* = 2),[16,32] and people (might include adults and children) with severe traumatic brain injury (*k* = 1).[30] The studies showed the effect of ACT on experiential avoidance (*k* = 24),[9,11-19,22,23,26-37] value-based living (*k* = 9),[9,21,22,31-35,37] mindfulness (*k* = 7),[12,21,22,27,31,32,35] and cognitive fusion (*k* = 6),[13,14,16,27,35,37] depressive symptoms (*k* = 16),[10-12,14,15,17,20,21,25-29,35-37] anxiety symptoms (*k* = 15), [10-12,14,15,17,20,25-30,36,37] stress symptoms (*k* = 20).[9-12,17-19,21-24,27-29,31-34,36,37] The first post-intervention points were at immediately post-intervention (*k* = 23)[10-17,19,21-30,32,33,35,37] and 2 weeks to 4 weeks post-intervention (*k* = 5).[9,18,31,34,36] The follow-up was at 4 weeks to 24 weeks post-intervention (*k* =20).[9-11,13,15,17,18,20,23-27,30-32,34,36,37]

eResults 2. Interventions Characteristics of Included Studies

All studies developed their interventions based on the ACT model. Most of the studies (83%) reported ACT especially training/experience of the facilitators while without details about trained skills and performance feedback, such as the workshop, skill training, knowledge learning, supervision meetings, and role-play. In the minority of the studies (*k* = 11) that performed the fidelity checks, randomly selected audio or video recordings for checked were mainly adopted (*k* = 8),[11,17,30,31,33,34,36,37] followed by paper-and-pencil fidelity checklists based on observation (*k* = 3).[13,19,32] Most of the interventions were adapted to informal caregivers' contexts (62%), and involved additional educational support (59%). Almost half of the studies (55%) applied 6 processes of the ACT, followed by 5 processes (28%), and 4 processes (14%). Several materials were used to illustrate and understand the content of ACT interventions among 59% of eligible studies, including handouts, book text, notebook, worksheets, online materials (eg, text, video, picture, audio, message, feedback, discussion forum), and CD. Comparison groups comprised of education support (*k* = 14), [13-18,20,22,26,29,33,34,36,37] waiting list (*k* = 9),[10-12,23-25,27,30,32] usual care (*k* = 4),[9,19,31,35] and less intensive ACT (*k* = 2).[21,28] Two ACT intervention groups within 1 study were pooled into two records with a shared control group (*k* = 2).[23,31]

© 2023 Ye F et al. *JAMA Network Open.*

eTable 1. Search Strategies for Each Database

**PubMed**		
	Search queries	Results
#1	"Acceptance and Commitment Therapy"[MeSH Terms]	816
#2	(acceptance[Title/Abstract]) AND (commitment[Title/Abstract]) OR acceptance-based[Title/Abstract] OR "psychological flexib*"[Title/Abstract] OR "psychological inflexib*"[Title/Abstract]	3239
#3	#1 or #2	3282
#4	((((((Caregivers[MeSH Terms]) OR (Home Health Aides[MeSH Terms])) OR (Foster Home Care[MeSH Terms])) OR (Home nursing[MeSH Terms])) OR (Family[MeSH Terms])) OR (friends[MeSH Terms])) OR (family characteristic[MeSH Terms])	410,243
#5	(informal*[Title/Abstract] OR unpaid*[Title/Abstract] OR elder*[Title/Abstract] OR famil*[Title/Abstract] OR spous*[Title/Abstract] OR sibling[Title/Abstract] OR daughter[Title/Abstract] OR son[Title/Abstract] OR relativ*[Title/Abstract] OR dyad*[Title/Abstract] OR couple*[Title/Abstract] OR friend*[Title/Abstract] OR dependent*[Title/Abstract] OR parent*[Title/Abstract] OR kid[Title/Abstract] OR child*[Title/Abstract] OR partner*[Title/Abstract] OR carer*[Title/Abstract] OR caregiver*[Title/Abstract] OR communit*[Title/Abstract] OR home[Title/Abstract]) AND (care giv*[Title/Abstract] OR caregiv*[Title/Abstract] OR care*[Title/Abstract] OR caring[Title/Abstract] OR aide[Title/Abstract] OR support*[Title/Abstract] OR help*[Title/Abstract])	1,528,661
#6	#4 OR #5	1,786,180
#7	(randomized controlled trial[Publication Type]) OR (clinical trial[Publication Type])	961,747
#8	(Double-Blind Method[MeSH Terms]) OR (Single-Blind Method[MeSH Terms]) OR (Random Allocation[MeSH Terms]) OR (randomized controlled trial[MeSH Terms]) OR (controlled clinical trial[MeSH Terms])	487,547
#9	random*[Title/Abstract] OR blind*[Title/Abstract]	1,547,594
#10	(animals[MeSH Terms]) NOT (humans[MeSH Terms])	5,089,496
#11	(#7 OR #8 OR #9) NOT #10	1,929,214
#12	#3 AND #6 AND #11	241
**Embase**		
	Search queries	Results
#1	exp "acceptance and commitment therapy"/	2332
#2	((acceptance and commitment) or acceptance-based or "psychological flexib*" or "psychological inflexib*").mp	4731
#3	1 or 2	4731
#4	exp caregivers/ or exp home health aides/ or exp foster home Care/ or exp home nursing/ or exp family/ or exp friends/ or exp family characteristic/	2,696,916
#5	((informal* or unpaid* or elder* or famil* or spous* or sibling or daughter or son or relativ* or dyad* or couple* or friend* or dependent* or parent* or kid or child* or partner* or carer* or caregiver* or communit* or home) and (care giv* or caregiv* or care* or caring or support* or help* or aide)).mp.	2,886,865
#6	4 or 5	4,815,886
#7	exp randomized controlled trial/ or exp clinical trial/ or exp double-blind method/ or exp random allocation/ or exp single-blind method/	1,917,284
#8	(random* or blind*).mp.	2,436,377
#9	exp animals/ not exp humans/	5,886,050
#10	(7 or 8) not 9	3,113,548
#11	3 and 6 and 10	519
**APA PsycInfo**		
	Search queries	Results
#1	ab((acceptance and commitment) or acceptance-based or "psychological flexib*" or "psychological inflexib*") or ti((acceptance and commitment) or acceptance-based or "psychological flexib*" or "psychological inflexib*") or if((acceptance and commitment) or acceptance-based or "psychological flexib*" or "psychological inflexib*")	5159

(continued)

© 2023 Ye F et al. *JAMA Network Open.*

eTable 1. Search Strategies for Each Database (continued)

	Search queries	Results
**APA PsycInfo**		
	Search queries	Results
#2	ab((informal* or unpaid* or elder* or famil* or spous* or sibling or daughter or son or relativ* or dyad* or couple* or friend* or dependent* or parent* or kid or child* or partner* or carer* or caregiver* or communit* or home) and (care giv* or caregiv* or care* or caring or support* or help* or aide)) OR ti((informal* or unpaid* or elder* or famil* or spous* or sibling or daughter or son or relativ* or dyad* or couple* or friend* or dependent* or parent* or kid or child* or partner* or carer* or caregiver* or communit* or home) and (care giv* or caregiv* or care* or caring or support* or help* or aide)) OR if((informal* or unpaid* or elder* or famil* or spous* or sibling or daughter or son or relativ* or dyad* or couple* or friend* or dependent* or parent* or kid or child* or partner* or carer* or caregiver* or communit* or home) and (care giv* or caregiv* or care* or caring or support* or help* or aide))	631,593
#3	ab(random* or blind*) or ti(random* or blind*) or if(random* or blind*)	264,423
#4	1 and 2 and 3	166
**British Nursing Index**		
	Search queries	Results
#1	ab((acceptance and commitment) or acceptance-based or "psychological flexib*" or "psychological inflexib*") or ti((acceptance and commitment) or acceptance-based or "psychological flexib*" or "psychological inflexib*") or if((acceptance and commitment) or acceptance-based or "psychological flexib*" or "psychological inflexib*")	206
#2	ab((informal* or unpaid* or elder* or famil* or spous* or sibling or daughter or son or relativ* or dyad* or couple* or friend* or dependent* or parent* or kid or child* or partner* or carer* or caregiver* or communit* or home) and (care giv* or caregiv* or care* or caring or support* or help* or aide)) OR ti((informal* or unpaid* or elder* or famil* or spous* or sibling or daughter or son or relativ* or dyad* or couple* or friend* or dependent* or parent* or kid or child* or partner* or carer* or caregiver* or communit* or home) and (care giv* or caregiv* or care* or caring or support* or help* or aide)) OR if((informal* or unpaid* or elder* or famil* or spous* or sibling or daughter or son or relativ* or dyad* or couple* or friend* or dependent* or parent* or kid or child* or partner* or carer* or caregiver* or communit* or home) and (care giv* or caregiv* or care* or caring or support* or help* or aide))	149,149
#3	ab(random* or blind*) or ti(random* or blind*) or if(random* or blind*)	41,672
#4	1 and 2 and 3	10
**CINAHL Plus**		
	Search queries	Results
#1	AB((acceptance and commitment) or acceptance-based or "psychological flexib*" or "psychological inflexib*") or TI((acceptance and commitment) or acceptance-based or "psychological flexib*" or "psychological inflexib*") or MW((acceptance and commitment) or acceptance-based or "psychological flexib*" or "psychological inflexib*")	598
#2	AB((informal* or unpaid* or elder* or famil* or spous* or sibling or daughter or son or relativ* or dyad* or couple* or friend* or dependent* or parent* or kid or child* or partner* or carer* or caregiver* or communit* or home) and (care giv* or caregiv* or care* or caring or support* or help* or aide)) OR TI((informal* or unpaid* or elder* or famil* or spous* or sibling or daughter or son or relativ* or dyad* or couple* or friend* or dependent* or parent* or kid or child* or partner* or carer* or caregiver* or communit* or home) and (care giv* or caregiv* or care* or caring or support* or help* or aide)) OR MW((informal* or unpaid* or elder* or famil* or spous* or sibling or daughter or son or relativ* or dyad* or couple* or friend* or dependent* or parent* or kid or child* or partner* or carer* or caregiver* or communit* or home) and (care giv* or caregiv* or care* or caring or support* or help* or aide))	165,784
#3	AB(random* or blind*) or TI(random* or blind*) or MW(random* or blind*)	104,357
#4	1 and 2 and 3	32

(continued)

© 2023 Ye F et al. *JAMA Network Open.*

eTable 1. Search Strategies for Each Database (continued)

**Web of Science**		
	Search queries	Results
#1	TS = ((acceptance and commitment) or acceptance-based or "psychological flexib*" or "psychological inflexib*")	7925
#2	TS = ((informal* or unpaid* or elder* or famil* or spous* or sibling or daughter or son or relativ* or dyad* or couple* or friend* or dependent* or parent* or kid or child* or partner* or carer* or caregiver* or communit* or home) and (care giv* or caregiv* or care* or caring or support* or help* or aide))	2,374,784
#3	TS = (random* or blind*)	2,594,349
#4	1 and 2 and 3	360
**Cochrane Library**		
	Search queries	Results
#1	MeSH descriptor: [Acceptance and Commitment Therapy] explode all trees	356
#2	((acceptance and commitment) or acceptance-based or "psychological flexib*" or "psychological inflexib*"):ti,ab,kw	1863
#3	#1 or #2	1863
#4	MeSH descriptor: [caregivers] explode all trees	3141
#5	MeSH descriptor: [Home Health Aides] explode all trees	27
#6	MeSH descriptor: [Foster Home Care] explode all trees	177
#7	MeSH descriptor: [Family] explode all trees	12,627
#8	MeSH descriptor: [friends] explode all trees	188
#9	MeSH descriptor: [family characteristic] explode all trees	1607
#10	MeSH descriptor: [Home Nursing] explode all trees	323
#11	(Informal* or unpaid* or elder* or famil* or spous* or sibling or daughter or son or relativ* or dyad* or couple* or friend* or dependent* or parent* or kid or child* or partner* or carer* or caregiver* or communit* or home):ti,ab,kw AND (care giv* or caregiv* or care* or caring or support* or help* or aide):ti,ab,kw	178,805
#12	#4 or #5 or #6 or #7 or #8 or #9 and #10 or #11	184,030
#13	(random* or blind*):ti,ab,kw	1,278,165
#14	#3 and #12 and #13	370
	Trials	361

(continued)

© 2023 Ye F et al. *JAMA Network Open.*

eTable 1. Search Strategies for Each Database (continued)

China National Knowledge Infrastructure (CNKI)		
	Search queries in Chinese [translation in English]	Results
#1	SU%(接纳承诺 [acceptance and commitment] + 接受承诺 [synonyms for “acceptance and commitment”] + 心理灵活性 [psychological flexibility])	636
#2	SU%(非正式 [informal] + 无偿 [unpaid] + 居家 [home]+ 家庭 [family]+ 家属 [family member] + 亲属 [relative] + 配偶 [spouse] + 朋友 [friend] + 父母 [parent] + 子女 [child] + 兄弟 [brother] + 姐妹 [sister] + 亲戚 [synonyms for relative] + 亲人[synonyms for relative] + 社区 [community] + 照顾 [caregiving] + 照料 [synonyms for caregiving] + 看护[synonyms for caregiving] + 照看 [synonyms for caregiving] + 护理 [nursing] + 照护 [synonyms for caregiving] + 养老 [proving care for the elderly] + 支持 [support] + 帮助 [help] + 助手 [helper])	4,060,170
#3	1 and 2	230
**Wanfang Database**		
	Search queries in Chinese [translation in English]	Results
#1	主题:(接纳承诺 [acceptance and commitment] or 接受承诺[synonyms for “acceptance and commitment”] or 心理灵活性 [psychological flexibility])	3465
#2	主题:(非正式 [informal] or 无偿 [unpaid] or 居家 [home] or 家庭 [family] or 家属 [family member] or 亲属 [relative] or 配偶 [spouse] or 朋友 [friend] or 父母 [parent] or 子女 [child] or 兄弟 [brother] or 姐妹 [sister] or 亲戚 [synonyms for relative] or 亲人 [synonyms for relative] or 社区 [community] or 照顾 [caregiving] or 照料 [synonyms for caregiving] or 看护[synonyms for caregiving] or 照看 [synonyms for caregiving] or 护理 [nursing] or 照护 [synonyms for caregiving] or 养老 [proving care for the elderly] or 支持 [support] or 帮助 [help] or 助手 [helper])	7,576,048
#3	1 and 2	1073
**Weipu Database**		
	Search queries in Chinese [translation in English]	Results
#1	(M = (非正式 [informal] or 无偿 [unpaid] or 居家 [home] or 家庭 [family] or 家属 [family member] or 亲属 [relative] or 配偶 [spouse] or 朋友 [friend] or 父母 [parent] or 子女 [child] or 兄弟 [brother] or 姐妹 [sister] or 亲戚 [synonyms for relative] or 亲人 [synonyms for relative] or 社区 [community] or 照顾 [caregiving] or 照料 [synonyms for caregiving] or 看护[synonyms for caregiving] or 照看 [synonyms for caregiving] or 护理 [nursing] or 照护 [synonyms for caregiving] or 养老 [proving care for the elderly] or 支持 [support] or 帮助 [help] or 助手 [helper])) AND (M = (接纳承诺 [acceptance and commitment] or 接受承诺[synonyms for “acceptance and commitment”] or 心理灵活性 [psychological flexibility]) or R = (接纳承诺 [acceptance and commitment] or 接受承诺 [synonyms for “acceptance and commitment”] or 心理灵活性 [psychological flexibility]))	433

© 2023 Ye F et al. *JAMA Network Open.*

eTable 2. Characteristics of Study Design and Participants of Included Studies

Author (Year)/ Country	Study setting	Research topic	Health context for caregiving	Sample size[a]	Target participants/ Age (Mean ± SD, IG/CG)[a]	Female (IG/CG)[a]	Psychological flexibility-related outcomes (Measures)	Other psychological health outcomes (Measures)	Evaluation points of the outcomes	Attrition rate (IG/CG)[b]
Han *et al. (2023)*/ USA[37]	Community	ACT for distressed family caregivers	People with dementia	19	Informal caregivers/ 56.0±20.1 / 53.2±10.7	100%/ 100%	Experiential avoidance (AAQ-II) Cognitive fusion (CFQ) Value-based living (EMAS)	Depressive (DASS-21-D), Anxiety (DASS-21-A), Stress symptoms (DASS-21-S)	Pre- & Post-intervention, 1-month follow-up	0%/ 10%
Çiçek Gü müş & Öncel (2022)/ Turky[29]	Community	ACT for parents' mental states	Special needs children	60	Informal caregivers /NA/NA	100%/ 100%	Experiential avoidance (AAQ-II)	Depressive (DASS-21-D), Anxiety (DASS-21-A), Stress symptoms (DASS-21-S)	Pre- & Post-intervention, 3-month follow-up	0%/ 0%
Hadavand et al. (2022)/ Turky[30]	Hospital	ACT for caregivers' family function and emotional problems	Severe Traumatic Brain Injury	40	Informal caregivers/ 32.70±3.24 (all participants)	90%/ 100%	Experiential avoidance (EACQ-Active avoidant behaviors)	Anxiety symptoms (DASS-21-A)	Pre- & Post-intervention, 3-month & 6-month follow-up	0%/ 0%
Jin *et al.* (2022)/ Hong Kong[19]	Inpatient department	ACT for parents' psychological distress	Children with cancer	40	Informal caregivers/ 34.0±4.3/ 35.4 ±4.7	80%/ 80%	Experiential avoidance (AAQ-II)	Stress symptoms (DASS-21)	Pre- & Post-intervention	5%/ 5%
Mosher *et al.* (2022)/ USA[34]	Community[c]	ACT for patient fatigue interference and caregiver burden	Gastrointestinal cancer	40	Dyads/ 55.6±14.9/ 48.6±15.8	85%/ 65%	Experiential avoidance (AAQ-II), Value-based living (VQ)	Stress symptoms (ZBI)	Pre- & Post-intervention (2-week post-intervention), 3-month follow-up	25%/ 10%

(continued)

© 2023 Ye F et al. *JAMA Network Open.*

eTable 2. Characteristics of Study Design and Participants of Included Studies (continued)

Author (Year)/ Country	Study setting	Research topic	Health context for caregiving	Sample size[a]	Target participants/ Age (Mean ± SD, IG/CG)[a]	Female (IG/CG)[a]	Psychological flexibility-related outcomes (Measures)	Other psychological health outcomes (Measures)	Evaluation points of the outcomes	Attrition rate (IG/CG)[b]
Whittingham *et al.* (2022)/ Australia[12]	Community	ACT for parenting	Children with cerebral palsy	67	Informal caregivers /NA/NA	89%/ 100%	Experiential avoidance (AAQ-II) Mindfulness (IM-P)	Depressive (DASS-D), Anxiety (DASS-A), Stress symptoms (DASS-S)	Pre- & Post-intervention	19%/ 3%
Köhle *et al.* (2021)/ Netherlands[23]	Community	ACT and self-compassion training for partners' distress	Cancer	203	Informal caregivers/ PF: 57.0±9.9/ AF: 56.4±11.2/ CG: 54.2±11.0	PF: 70%/ AF: 71%/ CG: 70%	Experiential avoidance (AAQ-II)	Stress symptoms (HADS)	Pre- & Post-intervention, 6-month follow-up	PF: 28%/ AF: 44%/ CG:21%
Lappalainen *et al.* (2021)/ Finland[21]	Community	ACT for parents' burnout and distress	Children with chronic conditions	110	Informal caregivers/ 38.9±6.9/ 41.5±6.3	95%/ 93%	Value-based living (CompACT-VA), Mindfulness (FFMQ)	Depressive (PHQ-9), Stress symptoms (RAND-36-M)	Pre- & Post-intervention	26%/ 25%
Marino *et al.* (2021)/ Italy[22]	Community	ACT for improving psychological well-being in Parents	Children with ASD	40	Informal caregivers/ 40.6±5.34/ 42.0 ±5.7	50%/ 50%	Experiential avoidance (AAQ-II), Value-based living (VLQ), Mindfulness（MAAS）	Stress symptoms (PSI)	Pre- & Post-intervention	0%/ 0%

(continued)

© 2023 Ye F et al. *JAMA Network Open.*

eTable 2. Characteristics of Study Design and Participants of Included Studies (continued)

Author (Year)/ Country	Study setting	Research topic	Health context for caregiving	Sample size[a]	Target participants/ Age (Mean ± SD, IG/CG)[a]	Female (IG/CG)[a]	Psychological flexibility-related outcomes (Measures)	Other psychological health outcomes (Measures)	Evaluation points of the outcomes	Attrition rate (IG/CG)[b]
Potter *et al.* (2021)/ UK[31]	Community	ACT for caregivers' strain	Multiple Sclerosis	24	Informal caregivers/ SH: 53.5±12.6/ SH+: 50.2±7.0/ CG: 58.4±9.4	SH: 78%/ SH+: 57%/ CG: 100%	Experiential avoidance (AAQ-II), Value-based living (CompACT-VA), Mindfulness (ComACT-BA)	Stress symptoms (ZBI)	Pre- & Post-intervention (1-month post-intervention), 4-month follow-up	SH: 22%/ SH+: 29%/ CG: 0%
B. Yuan (2021)/ Mainland China[14]	Community	ACT For parents' mental health	Children with ASD at First Diagnosis	70	Informal caregivers/ 30.7±4.4/ 30.3±5.7	49%/ 54%	Experiential avoidance (AAQ-II), Cognitive fusion (CFQ)	Depressive (SDS), Anxiety symptoms (SAS)	Pre- & Post-intervention	0%/ 0%
Drouillard *et al.* (2020)/ Canada[13]	Community	ACT for supporting treatment selection in parents	Children with ASD	23	Informal caregivers/ 41.4±7.7/ 46.4±12.7	78% for all	Experiential avoidance (AAQ-II), Cognitive fusion (CFQ-A)	/	Pre- & Post-intervention, 3-month follow-up	38%/ 52%
De Wit *et al.* (2020)/ Netherlands[24]	Community	Blended psychosocial support for partners' distress	ALS and PMA	148	Informal caregivers/ 61.8±10.6/ 61.3±9.8	65%/ 65%	/	Stress symptoms (HADS)	Pre- & Post-intervention, 3-month follow-up	24%/ 19%
Davis *et al.* (2020)/ Australia[9]	Palliative care units in hospital	Self-help ACT for grief and psychological distress in caregivers	Palliative care patients	106	Informal caregivers/ 59.0±15.6/ 57.3±6.7	77%/ 65%	Experiential avoidance (AAQ-II), Value-based living (VLQ)	Stress symptoms (HADS)	Pre- & 1-month post-intervention, 6-month follow-up	51%/ 66%

(continued)

© 2023 Ye F et al. *JAMA Network Open.*

eTable 2. Characteristics of Study Design and Participants of Included Studies (continued)

Author (Year)/ Country	Study setting	Research topic	Health context for caregiving	Sample size[a]	Target participants/ Age (Mean ± SD, IG/CG)[a]	Female (IG/CG)[a]	Psychological flexibility-related outcomes (Measures)	Other psychological health outcomes (Measures)	Evaluation points of the outcomes	Attrition rate (IG/CG)[b]
Jolley *et al.* (2020)/ UK[32]	Community	ACT for patients and caregivers targeting positive wellbeing and distress	Psychosis	41	Patients and informal caregivers (no need to be dyads)/ 52.7±9.1/ 50.5±14.4	95%/ 84%	Experiential avoidance (AAQ-II), Value-based living (VQ), Mindfulness (SMQ)	Stress symptoms (CORE-10)	Pre- & Post-intervention, 2-month follow-up	27%/ 47%
Marquez-Gonzalez *et al.* (2020)/ Spain[25]	Community	Tailored versus manualized interventions for caregivers' distress	Dementia	45	Informal caregivers/ 56.9±14.8/ 60.5 ±11.2	95%/ 75%	/	Depressive symptoms (CES-D), Anxiety symptoms (POMS-A)	Pre- & Post-intervention, 6-month follow-up	24%/ 63%
Wang (2020)/ Mainland China[15]	Rehabilitation department in hospital	ACT for caregivers' mental health	Children with cerebral palsy	144	Informal caregivers/ 38.3±5.1/ 37.8±4.9	64%/ 65%	Experiential avoidance (AAQ-II)	Depressive symptoms (SDS), Anxiety symptoms (SAS)	Pre- & Post-intervention, 1-month & 3-month follow-up	0%/ 0%
X.X. Xu *et al*. (2020)/ Mainland China[20]	Inpatient in the hospital	ACT for parents' psychological health	Children with leukemia	89	Informal caregivers/ 35.3±4.0/ 36.3±4.1	63%/ 72%	/	Depressive symptoms (SDS), Anxiety symptoms (SAS)	Pre-intervention, 3-month follow-up	9%/ 2%

(continued)

© 2023 Ye F et al. *JAMA Network Open.*

eTable 2. Characteristics of Study Design and Participants of Included Studies (continued)

Author (Year)/ Country	Study setting	Research topic	Health context for caregiving	Sample size[a]	Target participants/ Age (Mean ± SD, IG/CG)[a]	Female (IG/CG)[a]	Psychological flexibility-related outcomes (Measures)	Other psychological health outcomes (Measures)	Evaluation points of the outcomes	Attrition rate (IG/CG)[b]
J. Xu (2020)/ Mainland China[16]	Inpatient or outpatient in the hospital	ACT in family function	The first episode schizophrenia	64	Dyads/ 44.1±4.9/ 41.3±6.9	63%/ 63%	Experiential avoidance (AAQ-II), Cognitive fusion (CFQ)	/	Pre- & Post-intervention	6%/ 6%
Chong *et al.* (2019)/ Hong Kong[17]	Community	ACT for parental management of childhood asthma and mental health	Children with Asthma	168	Informal caregivers/ 37.7±5.6/ 39.1 ±6.2	90%/ 86%	Experiential avoidance (AAQ-II)	Depressive (DASS-21-D), Anxiety (DASS-21-A), Stress symptoms (DASS-21-S)	Pre- & Post-intervention, 6-month follow-up	5%/ 4%
Hahs *et al.* (2019)/ USA[35]	Community	ACT for parents' mental health	Children with ASD	18	Informal caregivers/ 43.8±4.6/ 47.2±7.2	78%/ 67%	Experiential avoidance (AAQ-II), Cognitive fusion (CFQ), Mindfulness (MAAS), Value-based living (PVQ-II)	Depressive symptoms (BDI-II)	Pre- & Post-intervention	0%/ 0%
Mosher *et al.* (2019)/ USA[36]	Community	ACT for symptom interference and caregivers' distress	Advanced lung cancer	50	Dyads/ 61.6±11.5/ 52.4±18.1	76%/ 84%	Experiential avoidance (PA subscale)	Depressive (PROMIS-D), Anxiety (PROMIS-A), Stress symptoms (Thermometer)	Pre- & 2-week post-intervention, 6-week follow-up	20%/ 28%

(continued)

© 2023 Ye F et al. *JAMA Network Open.*

eTable 2. Characteristics of Study Design and Participants of Included Studies (continued)

Author (Year)/ Country	Study setting	Research topic	Health context for caregiving	Sample size[a]	Target participants/ Age (Mean ± SD, IG/CG)[a]	Female (IG/CG)[a]	Psychological flexibility-related outcomes (Measures)	Other psychological health outcomes (Measures)	Evaluation points of the outcomes	Attrition rate (IG/CG)[b]
Sairanen *et al.* (2019)/ Sweden[27]	Community	ACT for parents' mental health	Children with chronic health conditions	78	Informal caregivers/ 43.0±8.2/ 42.3±5.5	89%/ 73%	Experiential avoidance (AAQ-II), Cognitive fusion (CFQ), Mindfulness (FFMQ)	Depressive (DASS-D), Anxiety (DASS-A), Stress symptoms (DASS-S)	Pre-, mid- & Post-intervention, 4-month follow-up	31%/ 36%
Xiao (2018)/ Mainland China[18]	Department of hematology in the hospital	ACT for parents' mental health	Children with leukemia with chemotherapy	80	Informal caregivers/ <40 years old (82%)/ <40 years old (78%)	53%/ 67%	Experiential avoidance (AAQ-II)	Stress symptoms (PCL-C)	Pre- & 3-week post-intervention, 6-week follow-up	15%/ 10%
Kanstrup *et al.* (2016)/ Sweden[28]	Community	ACT for adolescents with chronic pain and supporting parents	Adolescents with chronic pain	28	Dyads/ 48.4±4.5/ 46.5±5.0/	83%/ 88%	Experiential avoidance (PPFQ) [d]	Depressive (HADS-D) [d], Anxiety (HADS-A) [d] Stress symptoms (HADS) [d]	Pre-, Mid- & Post-intervention	54% in all
Lloyd (2016)/ UK[33]	Rehabilitati on hospital	The use of ACT to address psychological stress experienced by caregivers	Adults with acquired brain injury	18	Informal caregivers/ 46.2±14.64/ 52.4 ±14.4	90%/ 88%	Experiential avoidance (AAQ-II), Value-based living (VQ),	Stress symptoms (GHQ-12)[d]	Pre- & Post-intervention	50%/ 38%

(continued)

© 2023 Ye F et al. *JAMA Network Open.*

eTable 2. Characteristics of Study Design and Participants of Included Studies (continued)

Author (Year)/ Country	Study setting	Research topic	Health context for caregiving	Sample size[a]	Target participants/ Age (Mean ± SD, IG/CG)[a]	Female (IG/CG)[a]	Psychological flexibility-related outcomes (Measures)	Other psychological health outcomes (Measures)	Evaluation points of the outcomes	Attrition rate (IG/CG)[b]
Whittingham *et al.* (2016)/ Australia[10]	Community	Parenting intervention combined With ACT for parents' mental health	Children With Cerebral Palsy	45	Informal caregivers/ 37.9±9.4/ 39.7±6.1	100%/ 90%	/	Depressive (DASS-42-D), Anxiety (DASS-42-A), Stress symptoms (DASS-42-S)	Pre- & Post-intervention, 6-month follow-up	9%/ 14%
Brown *et al.* (2015)/ Australia[11]	Paediatric rehabilitation in the hospital	Does Stepping Stones Triple P + ACT improve parent, couple, and family adjustment	Children with acquired brain injury	59	Informal caregivers/ 38.9±6.4/ 39.4±6.0	90%/ 90%	Experiential avoidance (AAABIQ)	Depressive (DASS-42-D), Anxiety (DASS-42-A), Stress symptoms (DASS-42-S)	Pre- & Post-intervention, 6-month follow-up	17%/ 7%
Losada *et al.* (2015)/ Spain[26]	Community	CBT versus ACT for informal caregivers' depressive symptoms	Dementia	93	Informal caregivers/ 61.7±15.3/ 62.3±12.9	82%/ 81%	Experiential avoidance (EACQ)	Depressive (CES-D), Anxiety symptoms (POMS-A)	Pre- & Post-intervention, 6-month follow-up	27%/ 35%

Abbreviations: AAABIQ, The Acceptance and Action for acquired brain injury Questionnaire; AAQ-II, the Acceptance and Action Questionnaire-II; AF, web-based ACT with automatic feedback; ALS, Amyotrophic Lateral Sclerosis; ASD, Autism Spectrum Disorders; BDI-II, the Beck Depression Inventory-II; CES-D, Center for Epidemiologic Studies Depression Scale; CFQ-A, the Cognitive Fusion Questionnaire for Autism; CG, control group; CompACT-BA, behavioral awareness of Comprehensive Assessment of Acceptance and Commitment therapy subscale; CompACT-VA, valued action of CompACT subscale; CompACT-OE, openness to experience or experience avoidance of CompACT subscale; CORE-10, Clinical outcomes in Routine Evaluation; DASS-21, the Depression, Anxiety, and Stress Scale-21 items; EACQ, Experiential Avoidance in Caregiving; EMAS, the Engagement in Meaningful Activities; FFMQ, the Five Facet Mindfulness Questionnaire; HADS, the Hospital Anxiety and Depression Scale; IG, intervention group; IM-P: Interpersonal Mindfulness in Parenting Scale; MAAS, the Mindfulness Attention Awareness Scale; NA, not available; PA, Peaceful Acceptance subscale; PCL-C, Post-traumatic Stress Disorder (PTSD) Checklist-civilian Version; PF, web-based ACT with personal feedback; PHQ-9, Patient Health Questionnaire; PMA, Progressive Muscular Atrophy; POMS, the Tension-Anxiety subscale from the Profile of Mood States; PPFQ, Parent Psychological Flexibility Questionnaire; PROMIS, the Patient-Reported Outcomes Measurement Information System; PSI, the Parental Stress Index; PVQ-II, the Personal Values Questionnaire-II; RAND-36-M: mental health subscale of 36-Item Short-Form Health Survey covering psychological distress and well-being; SAS, Self-Rating Anxiety Scale; SDS, Self-Rating Depression Scale; SH, self-help; SH+, enhanced self-help; VLQ, the Valued Living Questionnaire; VQ, the Valuing Questionnaire; ZBI, Zarit Burden interview.

[a] Characteristics for informal caregivers of included intervention and control groups.
[b] Attrition rate regarding informal caregivers who complete the first post-intervention assessment.
[c] Including clinics, medical centers, and local community organizations.
[d] Using non-parametric test and median.

© 2023 Ye F et al. *JAMA Network Open.*

eTable 3. Interventions Characteristics of Included Studies

Studies	Sources of protocol development	Facilitators	Fidelity check	Adaptation of the ACT content	Delivery format and modality	ACT process (No.)[a]	Additional education	Duration/ Sessions/ Time of each session/ Encounter time[b]	Materials	Control group
Han *et al.* (2023)[37]	ACT protocols, ACT model, books	A professional counselor	Protocol developer review counselor's session logs	Based on preliminary study	Individual Video-conference	6	Dementia caregiving	8 weeks/ 8 sessions/ 60 mins/ 480 mins	Hard copies of ACT session worksheets, video	Dementia caregiving
Çiçek Gümüş & Öncel (2022)[29]	Literature, ACT protocols, books	Nurse	/	Pilot test among 2 parents	Individual face-to-face	6	Psycho-education	6 weeks/ 6 sessions/ 60 mins/ 360 mins	A4 Paper, worksheet, pencils, clipboard, rope, notebook	Psycho-education
Hadavand et al. (2022)[30]	Book	Clinical psychologist (weekly supervision from two clinical psychologist with PhD)	Audio-recorded sessions were reviewed weekly by supervisors	/	Group-based (5-7 participants/ group) face-to-face	/	/	10 weeks/ 10 sessions/ 60 mins/ 600 mins	/	Wait list
Jin *et al.* (2022)[19]	Training books and manuals	PhD nursing students (weekly meeting with supervisor)	Recordings were randomly reviewed by an ACT researcher	/	Individual face to face or synchronize d videos	6	/	4 weeks/ 4 sessions/ 45-60 mins/ 210 mins	Booklet, audio instructions, and a mindfulness log	Usual care

(continued)

© 2023 Ye F et al. *JAMA Network Open.*

eTable 3. Interventions Characteristics of Included Studies (continued)

Studies	Sources of protocol development	Facilitators	Fidelity check	Adaptation of the ACT content	Delivery format and modality	ACT process (No.)[a]	Additional education	Duration/ Sessions/ Time of each session/ Encounter time[b]	Materials	Control group
Mosher *et al.* (2022)[34]	Literatures, ACT model, and clinical experience	Clinicians and psychologist	Recordings were random reviewed by expertise	Adapting to dyad	Mixed individual and group-based telephone call	6	Education about fatigue management	6 weeks/ 6 sessions/ 50 mins/ 300 mins	Handouts for session topics	Education/ support by the social worker
Whittingham *et al.* (2022)[12]	Literatures and ACT model	Self-help (Average 10 mins of consultation with a psychologist)	Developed content for self-help	Focusing on parenting	Online course	6	/	10 weeks/ 5 sessions/ 60-120min/ 10 mins	Online text, video, activities, exercises, discussion board	Waitlist control
Köhle *et al.* (2021)[23]	Interviews, survey, ACT model, and self-compassion	Self-help with feedback and consultation (Through email)	Developed content for self-help	Participants co-created, adapting to participants ' need	IG1: Online SH + online written PF IG2: Online SH + online written AF	5 (without self-as-context)	Psycho-education	12 weeks/ 6 sessions+2 optionals/ 60-90 mins/ NA	Online text, audio, optional weekly message	Waitlist control and receiving relevant disease society websites
Lappalainen *et al.* (2021)[21]	Literatures	Self-help + Psychologist online meeting	/	Adapting to parents specially	Online SH + video conference	6	/	13 weeks/ 5 sessions/ NA/ 135 mins	Online text, video, picture, and audio exercise	SH booklet by email + online exercise
Marino *et al.* (2021)[22]	ACT matrix model	Expert therapist	/	Group homework was specifically designed	Group-based face-to face	5 (Without self-as-context)	Education about the behavioral characteristics of children, Verbal Aikido[d]	24 weeks/ 24 sessions/ 90 mins/ 2160 mins	/	Parent training for behavioral managem ent skills

(continued)

© 2023 Ye F et al. *JAMA Network Open.*

eTable 3. Interventions Characteristics of Included Studies (continued)

Studies	Sources of protocol development	Facilitators	Fidelity check	Adaptation of the ACT content	Delivery format and modality	ACT process (No.)[a]	Additional education	Duration/ Sessions/ Time of each session/ Encounter time[b]	Materials	Control group
Potter *et al.* (2021)[31]	Self-help ACT book[c]	Self-help with telephone support	Audio-recorded assessment	/	IG1: email<br>IG2: email + weekly telephone support	6	/	8 weeks/<br>8 sessions/<br>NA/<br>IG1:0 min,<br>IG2: 136 mins	Book texts	Usual care
Yuan (2021)[14]	Book for introduction of ACT[e]	Psychiatrist	/	/	Individual face-to-face	6	Education about ASD	7 weeks/<br>7 sessions/<br>80 mins/<br>560mins	/	Individual psycho-education
Drouillard *et al.* (2020)[13]	Literature and ACT model	Psychology PhD student	Treatment fidelity measures	Adapting to the experience of parenting	Group-based face-to-face	6	Education about ASD	One day/<br>one session/<br>390 mins/<br>390 mins	Values and goals worksheet	Group-based education and support
De Wit *et al.* (2020)[24]	Interviews and ACT model	Psychologist	Developed content for self-help	Adapting to caregivers' needs	Face-to-face + online SH + telephone support	5 (Without self-as-context)	Psycho-education, Communication skill	12 weeks/<br>6 sessions/<br>90 mins/<br>90 mins	Online text	Waitlist control
Davis *et al.* (2020)[9]	Caregivers' and experts' consultation and ACT model	Self-help With telephone support	Developed content for self-help	Adapting to caregivers' needs based on consultation and feedback	SH + telephone support	4 (Without self-as-context and cognitive defusion)	Psycho-education	4 weeks/<br>10 sessions/<br>NA/<br>5mins	Booklet and CD	Usual care and receiving psychosocial support by social workers

(continued)

© 2023 Ye F et al. *JAMA Network Open.*

eTable 3. Interventions Characteristics of Included Studies (continued)

Studies	Sources of protocol development	Facilitators	Fidelity check	Adaptation of the ACT content	Delivery format and modality	ACT process (No.)[a]	Additional education	Duration/ Sessions/ Time of each session/ Encounter time[b]	Materials	Control group
Jolley *et al.* (2020)[32]	Literatures and ACT model	Trained peers (service-user experts) and frontline staff	/	/	Group-based face-to-face	5 (Without self-as-context)	/	8 weeks/ 4 sessions +2 boosters/ 120 mins/ 480 mins	Video, audio exercises, worksheet, homework materials	Waitlist control
Marquez-Gonzalez *et al.* (2020)[25]	Literatures and ACT model	Clinical psychologist	/	Adapting to dementia caregiver	Individual face-to-face	4 (Without being present and self-as-context)	/	8 weeks / 8 sessions +3 boosters/ 90 mins/ 720 mins	/	Waitlist control
Wang (2020)[15]	Book for introduction of ACT [e]	Master Nursing student	/	/	Individual/ mini group-based face-to-face	6	Disease education	8 weeks/ 8 sessions/ 60-80 mins/ 660mins	Notebook, exercise tools	Usual care and education support
X.X. Xu *et al.* (2020)[20]	ACT model	Nurse	/	Adapting to parents' need	Individual face-to-face	6	Disease education	NA/ NA/ 40mins/ NA	/	Usual care and disease education
J. Xu (2020)[16]	ACT model	Master psychology student	/	/	Group-based face-to-face	5 (Without self-as-context)	Disease education	8 weeks/ 8 sessions/ 50 mins/ 400 mins	/	Disease education
Chong *et al.* (2019)[17]	Literatures, the interview, ACT model, and experts' consultation	Registered nurse	Video record	Adapting to parents' care experience	Group-based face-to-face	6	Disease education	4 weeks/ 4 sessions/ 120 mins/ 240 mins	Handout on ACT and asthma	Education by nurse + telephone call

(continued)

© 2023 Ye F et al. *JAMA Network Open.*

eTable 3. Interventions Characteristics of Included Studies (continued)

Studies	Sources of protocol development	Facilitators	Fidelity check	Adaptation of the ACT content	Delivery format and modality	ACT process (No.)[a]	Additional education	Duration/ Sessions/ Time of each session/ Encounter time[b]	Materials	Control group
Hahs *et al.* (2019)[35]	ACT model	Psychologist	/	/	Group-based face-to-face	6	/	2 weeks/ 2 sessions/ 120 mins/ 240 mins	/	Usual care
Mosher *et al.* (2019)[36]	ACT model	Master's level social worker	Audio record	Adapting to participants experiences	Telephone call	6	/	6 weeks/ 6 sessions/ 50 mins/ 300 mins	Handouts for session topic and CD	Education/ support by a PhD student
Sairanen *et al.* (2019)[27]	ACT model	Self-help	Developed content for self-help	Adapting to parents specially	Online SH + online written PF	6	/	10 weeks/ 5 sessions/ NA/ 0	Online text, video, picture, audio exercise, diary, discussion forum	Waitlist control
Xiao (2018) [18]	Book for introduction of ACT[e] and Experts' consultation	Master Nursing student	/	Adapting to parents' experience and pilot used	Individual face-to-face	6	Disease education	6 weeks/ 6 sessions/ 40-60mins/ 300 mins	/	Education
Kanstrup *et al.* (2016)[28]	Literatures and ACT model	Inter-disciplinary (psychologist, physician, physiotherapist, and clinic specialist)	/	Adapting to pain interference	Individual and dyads face-to-face	4 (Without being present and self-as-context)	Pain education	9 weeks/ 4 sessions/ 45 mins/ 180 mins	/	Group-based face-to-face ACT

(continued)

© 2023 Ye F et al. *JAMA Network Open.*

eTable 3. Interventions Characteristics of Included Studies (continued)

Studies	Sources of protocol development	Facilitators	Fidelity check	Adaptation of the ACT content	Delivery format and modality	ACT process (No.)[a]	Additional education	Duration/ Sessions/ Time of each session/ Encounter time[b]	Materials	Control group
Lloyd (2016)[33]	Literatures and ACT model	Clinical psychology PhD student	Audio record	/	Group-based face-to-face	5 (Without Self-as-context)	/	4 weeks/ 3 sessions/ 120 mins/ 360 mins	Handout	Enhanced treatment as usual
Whittingham *et al.* (2016)[10]	Literatures and ACT model	Psychologist	/	/	Group-based face-to-face + telephone support	5 (Without Self-as-context)	Disease education and teaching skill	One weeks/ 8 sessions/ 60 mins for ACT/ 270mins	/	Waitlist control
Brown *et al.* (2015)[11]	Literatures and ACT model	Psychologist	Session checklists , video- or audio-recored sessions	/	Group-based face-to-face + telephone support	5 (Without Self-as-context)	Education (eg, knowledge, skills, and communication)	10 weeks/ 8 sessions/ 120 min/ 1050 mins	/	Waitlist control
Losada *et al.* (2015)[26]	Literatures, caregivers' experience, and ACT model	Clinical psychologist	/	Adapting to caregivers	Individual face-to-face	4 (Without being present and self-as-context)	/	8 weeks/ 8 sessions/ 90 mins/ 720 mins	/	Booklet and psycho-education

Abbreviations: AF, automatic feedback; IG, intervention group; NA, not applicable; PF, personal feedback; SH, self-help; SH+, enhanced self-help.
[a] Identifiable process.
[b] Duration did not count the booster session during the follow-up; Time of therapeutic encounter is facilitator-participant interaction and contact time through in-person/virtual face-to-face or telephone call.
[c] "Get out of your mind and into your life: The new Acceptance and Commitment Therapy"[38]
[d] Verbal Aikido is a way of conflict management and communication skill.
[e] "ACT made simple: An easy-to-read primer on acceptance and commitment therapy"[39]

© 2023 Ye F et al. *JAMA Network Open.*

eTable 4. Overview of Study Characteristics of Included Studies

Author (year)	Characteristics														Outcome (PI and FU)						
			Caregiver		Care recipient		ACT content		Intervention delivery						Psychological flexibility				Others		
	Hospital	Sample size≥62	≤ 47 years old	Female (≥74%)	Adult	Children	ACT + additional education	6 ACT processes	Individual or mixed	Face-to-face	Non-self-help	Intervention duration≥8 weeks	≥6 Sessions	Encounter time [a] ≥ 300 mins	Experimental avoidance	Valued-based living	Mindfulness	Cognitive fusion	Depressive symptoms	Anxiety symptoms	Stress symptoms
Wang (2020)	√	√	√			√	√	√	√	√	√	√	√	√	$\downarrow^{L}$<br>$\downarrow^{L}$				$\downarrow^{L}$<br>$\downarrow^{L}$	$\downarrow^{L}$<br>$\downarrow^{L}$	
Çiçek Gümüs & Öncel (2022)			/	√		√	√	√	√	√	√		√	√	$\downarrow^{L}$<br>$\downarrow^{L}$				$\downarrow^{L}$<br>$\downarrow^{L}$	$\downarrow^{L}\downarrow^{L}$	$\downarrow^{L}$<br>$\downarrow^{L}$
Marino *et al.* (2021)			√			√	√			√	√	√	√	√	$\downarrow^{L}$<br>/	$\uparrow^{L}$<br>/	$\uparrow^{L}$<br>/				$\downarrow^{L}$<br>/
Yuan (2021)		√	√			√	√	√	√	√	√		√	√	$\downarrow^{L}$<br>/			$\downarrow^{L}$<br>/	$\downarrow^{L}$<br>/	$\downarrow^{L}$ /	
Marquez-Gonzalez *et al.* (2020)				√	√				√	√	√	√	√	√					$\downarrow^{L}$<br>$\downarrow^{L}$	$\downarrow^{L}\downarrow^{L}$	
Losada *et al.* (2015)		√		√	√				√	√	√	√	√	√	$\downarrow^{L}$<br>$\downarrow^{M}$				$\downarrow^{L}$<br>N	$\downarrow^{L}\downarrow$<br>M	
Xiao (2018)	√	√	√			√	√	√	√	√	√		√	√	$\downarrow^{L}$<br>$\downarrow^{L}$						N<br>$\downarrow^{L}$
Chong *et al.* (2019)		√	√	√		√	√	√		√	√				$\downarrow^{L}$<br>$\downarrow^{L}$				N<br>$\downarrow^{S}$	N<br>$\downarrow^{M}$	$\downarrow^{M}$<br>$\downarrow^{M}$
Hadavand et al. (2022)	√		√	√	/	/		/		√	√	√	√	√	$\downarrow^{L}$<br>$\downarrow^{L}$					$\downarrow^{L}\downarrow^{L}$	
J. Xu (2020)	√	√	√		√		√		√	√	√	√	√	√	$\downarrow^{L}$<br>/			$\downarrow^{M}$<br>/			
Lappalainen *et al.* (2021)		√	√	√		√		√	√	√	√	√			$\downarrow^{L}$<br>/	$\uparrow^{M}$<br>/	$\uparrow^{M}$<br>/		$\downarrow^{M}$<br>/		$\downarrow^{M}$<br>/
Brown *et al.* (2015)	√		√	√		√	√			√	√	√	√	√	$\downarrow^{M}$<br>/				N<br>/	$\downarrow^{M}$ /	$\downarrow^{M}$<br>/

(continued)

© 2023 Ye F et al. *JAMA Network Open.*

eTable 4. Overview of study Characteristics of Included Studies (continued)

Author (year)	Characteristics														Outcome (PI and FU)						
			Caregiver		Care recipient		ACT content		Intervention delivery						Psychological flexibility				Others		
	Hospital	Sample size≥62	≤ 47 years old	Female (≥74%)	Adult	Children	ACT + additional education	6 ACT processes	Individual or mixed	Face-to-face	Non-self-hlep	Intervention duration≥8 weeks	≥6 Sessions	Encounter time ≥ 300 mins	Experimental avoidance	Valued-based living	Mindfulness	Cognitive fusion	Depressive symptoms	Anxiety symptoms	Stress symptoms
Whittingham *et al.* (2016)			√	√		√	√			√	√		√						↓[M] /	↓[M]<br>/	↓[M]<br>/
Köhle *et al.* (2021)		√			√		√		√			√	√		↓[M]<br>↓[L]						↓[S]<br>N
X.X. Xu *et al.* (2020)	√	√	√			√	√	√	√	√	√								/<br>↓[M]	/<br>↓[M]	
Sairanen *et al.* (2019)		√	√	√		√		√	√			√			N<br>N		N<br>↑[L]	N<br>↓[M]	N ↓[L]	N<br>↓[M]	N<br>↓[L]
Potter *et al.* (2021)				√	√			√	√			√	√		N<br>↓[L]	↑[L]<br>↑[L]	N<br>N				N<br>N
Kanstrup *et al.* (2016)				√		√	√		√	√	√	√			↓[L]<br>/				N<br>/	N<br>/	N<br>/
Drouillard (2020)			√	√		√	√	√		√	√			√	N<br>N			N<br>↓[L]			
Hahs *et al.* (2019)			√			√		√		√	\				N<br>/	↑[L]<br>/	N<br>/	N<br>/	N<br>/		
Mosher *et al.* (2019)				√	√			√	√		√		√	√	N<br>N				N N	N<br>N	↓[M]<br>/
Han *et al.* (2023)				√	√		√	√	√		√	√	√	√	N<br>N	N<br>N		N<br>N	N<br>N	N<br>N	N<br>N
Davis *et al.* (2020)	√	√			√		√		√				√		N<br>N	N<br>N					N<br>N

(continued)

© 2023 Ye F et al. *JAMA Network Open.*

eTable 4. Overview of study Characteristics of Included Studies (continued)

Author (year)	Characteristics														Outcome (PI and FU)						
			Caregiver		Care recipient		ACT content		Intervention delivery						Psychological flexibility				Others		
	Hospital (setting)	Sample size≥62	≤ 47 years old	Female (≥74%)	Adult	Children	ACT + additional education	6 ACT processes	Individual or mixed	Face-to-face	Non-self-help	Intervention duration≥8 weeks	≥6 Sessions	Encounter time ≥ 300 mins	Experimental avoidance	Valued-based living	Mindfulness	Cognitive fusion	Depressive symptoms	Anxiety symptoms	Stress symptoms
Whittingham *et al.* (2022)		√	√	√		√		√	√			√			N /		N /		N /	N /	N /
Jin *et al.* (2022)	√		√	√		√		√	√	√	√				N /						N /
Mosher *et al.* (2022)				√	√		√	√	√		√		√	√	N N	N N					N N
Jolley *et al.* (2020)				√	√					√	√	√		√	N N	N N	N N				N N
De Wit *et al.* (2020)		√			√		√		√	√	√	√	√								N N
Lloyd (2016)				√	√					√	√			√	N /	N /					

Abbreviations: PI, post-intervention; FU, follow-up; ↓, significantly decreased; ↑, significantly increased; [L], a large effect size; [M], a moderate effect size; [S], a small effect size; [N], non-significant results.
[a] Time of therapeutic encounter is facilitator-participant interaction and contact time through in-person/virtual face-to-face or telephone call.

© 2023 Ye F et al. *JAMA Network Open.*

eTable 5. Sensitivity Analysis for Psychological Flexibility and Other Psychological Health Outcomes

Outcomes		Overall		Sensitivity analysis with the leave-one-out method							
				The Largest sample size (Excluding Ref)		The smallest sample size (Excluding Ref)		The largest effect size (Excluding Ref)		The smallest effect size (Excluding Ref)	
	No. of Studies	SMD (95% CI)	I^2, %	SMD (95% CI)	I^2, %	SMD (95% CI)	I^2, %	SMD (95% CI)	I^2, %	SMD (95% CI)	I^2, %
**Psychological flexibility**											
Experiential avoidance											
Post-intervention	23	-1.04 (-1.65, -0.44)	90	-1.06 (-1.69, -0.42)[17]	91	-1.13 (-1.73, -0.52)[33]	90	-0.83 (-1.27, -0.39)[29]	87	-1.13 (-1.73, -0.52)[33]	90
Follow-up	15	-1.17 (-1.85, -0.49)	89	-1.17 (-1.90, -0.43)[17]	90	-1.19 (-1.92, -0.47)[31]	90	-0.86 (-1.20, -0.52)[29]	80	-1.26 (-1.96, -0.56)[34]	89
Value-based living											
Post-intervention	9	0.98 (0.25, 1.71)	82	1.01 (0.17, 1.85)[21]	84	1.03 (0.22, 1.84)[33]	84	0.56 (0.21, 0.91)[22]	32	1.12 (0.34, 1.89)[34]	81
Follow-up	5	0.27 (-0.29, 0.84)	60	0.44 (-0.22, 1.11)[9]	62	0.09 (-0.40, 0.59)[31]	48	0.09 (-0.40, 0.59)[31]	48	0.46 (-0.12, 1.03)[34]	50
Mindfulness											
Post-intervention	7	1.19 (-0.23, 2.61)	88	1.27 (-0.40, 2.94)[21]	90	1.24 (-0.42, 2.89)[31]	90	0.46 (0.21, 0.70)[22]	0	1.38 (-0.24, 2.30)[32]	89
Follow-up	3	0.90 (0.30, 1.51)	44	0.63 (-0.08, 1.33)[27]	10	0.87 (0.13, 1.61)[31]	63	0.63 (-0.08, 1.33)[27]	10	1.23 (0.79, 1.67)[32]	0
Cognitive fusion											
Post-intervention	6	-0.61 (-1.06, -0.15)	70	-0.80 (-1.22, -0.37)[27]	41	-0.58 (-1.10, -0.07)[35]	75	-0.39 (-0.75, -0.02)[14]	19	-0.80 (-1.22, -0.37)[27]	41
Follow-up	3	-0.68 (-1.05, -0.30)	0	-0.57 (-1.18, 0.07)[27]	0	-0.77 (-1.18, -0.36)[37]	0	-0.77 (-1.18, -0.36)[37]	0	-0.64 (-1.06, -0.22)[13]	0

(continued)

© 2023 Ye F et al. *JAMA Network Open.*

eTable 5. Sensitivity Analysis for Psychological Flexibility and Other Psychological Health Outcomes (continued)

Outcomes	Overall			Sensitivity analysis with the leave-one-out method							
				The Largest sample size (Excluding Ref)		The smallest sample size (Excluding Ref)		The largest effect size (Excluding Ref)		The smallest effect size (Excluding Ref)	
	No. of Studies	SMD (95% CI)	I^2, %	SMD (95% CI)	I^2, %	SMD (95% CI)	I^2, %	SMD (95% CI)	I^2, %	SMD (95% CI)	I^2, %
**Other psychological health outcomes**											
Depressive symptoms											
Post-intervention	14	-1.14 (-1.83, -0.45)	93	-1.22 (-1.95, -0.49)[17]	93	-1.16 (-1.90, -0.42)[35]	94	-0.87 (-1.36, -0.38)[29]	91	-1.23 (-1.95, -0.52)[36]	94
Follow-up	9	-1.29 (-2.33, -0.24)	95	-1.39 (-2.57, -0.22)[17]	95	-1.31 (-2.49, -0.12)[25]	96	-0.87 (-1.58, -0.15)[29]	93	-1.46 (-2.59, -0.33)[36]	95
Anxiety symptoms											
Post-intervention	13	-1.02 (-1.57, -0.48)	90	-1.09 (-1.67, -0.51)[17]	90	-1.07 (-1.66, -0.49)[37]	91	-0.85 (-1.31, -0.39)[30]	89	-1.11 (-1.68, -0.55)[36]	90
Follow-up	10	-1.09 (-1.75, -0.44)	90	-1.14 (-1.88, -0.41)[17]	90	-1.11 (-1.84, -0.38)[25]	91	-0.89 (-1.45, -0.32)[30]	88	-1.21 (-1.89, -0.54)[37]	90
Stress symptoms											
Post-intervention	18	-0.55 (-0.98, -0.12)	82	-0.55 (-1.01, -0.10)[17]	83	-0.54 (-0.99, -0.09)[31]	83	-0.34 (-0.49, -0.19) [29]	36	-0.60 (-1.04, -0.16)[9]	81
Follow-up	12	-0.85 (-1.62, -0.08)	88	-0.88 (-1.72, -0.04)[17]	89	-0.83 (-1.66, -0.01)[31]	89	-0.45 (-0.66, -0.24) [29]	44	-0.94 (-1.76, -0.13)[37]	88

© 2023 Ye F et al. *JAMA Network Open.*

eTable 6. Subgroup Analysis for Psychological Health Outcomes at Postintervention

Design	Subgroup	No. of studies	Sample size	SMD (95% CI)	Heterogeneity			Subgroup differences	
					I^2, %	Q	*P*-value	Q	*P*-value
**Experiential avoidance**									
Chinese study	Yes	6	552	-1.30 (-1.81, -0.78)	85	33.59	< .01	0.44	.51
	No	17	870	-0.97 (-1.78, -0.16)	90	177.6	< .01		
Setting	Community [a]	15	994	-1.10 (-1.96, -0.25)	90	166.10	< .01	0.11	.95
	Hospital [b]	6	408	-1.06 (-1.66, -0.46)	87	37.82	< .01		
	Mixed setting	2	70	-0.52 (-3.94, 2.89)	95	18.96	< .01		
Risk of bias	Low	5	485	-0.43 (-0.73, 0.12)	56	11.24	.05	4.56	.10
	Some concerns	13	699	-1.25 (-1.98, -0.52)	90	126.67	< .01		
	High	5	238	-1.32 (-3.78, 1.15)	95	86.43	< .01		
Health context for caregiving	Children with chronic conditions	12	833	-1.59 (-2.76, -0.41)	94	173.42	< .01	6.39	.09
	Adults with CNS diseases	4	117	-0.42 (-1.09, 0.26)	57	9.29	.05		
	Adults with life-limiting diseases	4	348	-0.26 (-0.56, 0.04)	43	7.01	.14		
	Adults with the psychotic disorders	2	84	-1.43 (-2.99, 0.14)	89	8.79	< .01		
Intervention content	ACT	10	411	-0.52 (-0.96, -0.08)	73	37.26	< .01	3.01	.08
	ACT + disease/caregiving education	13	1011	-1.50 (-2.52, -0.48)	93	187.94	< .01		
No. of ACT processes	6	14	867	-0.92 (-1.65, -0.18)	91	151.21	< .01	0.71	.70
	5	6	396	-1.33 (-2.96, 0.31)	92	75.45	< .01		
	4	2	119	-0.61 (-1.40, 0.19)	77	4.38	.04		
Delivery format	Individual-based	13	856	-0.98 (-1.70, -0.26)	89	131.16	< .01	0.13	.94
	Group-based	8	382	-1.24 (-2.66, 0.18)	90	67.24	< .01		
	Mixed	2	184	-0.83 (-2.97, 1.3)	97	33.44	< .01		
Face-to-face	Yes	16	909	-1.51 (-2.42, -0.60)	91	167.20	< .01	7.21	.01
	No	7	513	-0.23 (-0.45, -0.01)	23	10.40	.24		
Self-help	Yes	5	423	-0.32 (-0.56, -0.08)	10	6.69	.35	5.15	.02
	No	18	999	-1.32 (-2.16, -0.49)	92	201.59	< .01		
Active control	Yes [c]	13	863	-1.56 (-2.67, -0.45)	94	189.09	< .01	2.91	.09
	No [d]	10	559	-0.55 (-0.90, -0.19)	66	32.47	< .01		

(continued)

© 2023 Ye F et al. *JAMA Network Open.*

eTable 6. Subgroup Analysis for Psychological Health Outcomes at Post-intervention (Continued)

Design	Subgroup	No. of studies	Sample size	SMD (95% CI)	Heterogeneity			Subgroup differences	
					I^2, %	Q	*P*-value	Q	*P*-value
**Depressive symptoms**									
Chinese study	Yes	3	382	-1.55 (-3.19, 0.09)	98	98.16	< .01	0.33	.57
	No	11	563	-1.02 (-1.80, -0.24)	89	93	< .01		
Setting	Community [a]	12	742	-1.04 (-1.75, -0.33)	90	106.47	< .01	0.19	.66
	Hospital [b]	2	203	-1.67 (-4.42, 1.08)	98	60.59	< .01		
Risk of bias	Low	4	282	-0.24 (-0.48, -0.01)	10	3.33	.34	7.93	.02
	Some concerns	8	529	-1.23 (-1.92, -0.54)	93	96.82	< .01		
	High	2	134	-2.55 (-7.03, 1.93)	98	63.09	< .01		
Health context for caregiving	Children with chronic conditions	10	787	-1.21 (-2.13, -0.30)	95	178.76	< .01	10.78	.005
	Adults with CNS diseases	3	108	-1.33 (-2.08, -0.57)	59	4.84	.09		
	Adults with life-limiting diseases	1	50	0.07 (-0.49, 0.62)	NA	NA	NA		
Intervention content	ACT	7	380	-0.70 (-1.20, -0.20)	77	25.48	< .01	1.47	.23
	ACT + disease/caregiving education	7	565	-1.54 (-2.82, -0.27)	96	162.59	< .01		
No. of ACT processes	6	10	752	-1.18 (-2.12, -0.23)	95	184.09	< .01	11.43	.003
	5	2	104	-0.42 (-0.81, -0.03)	0	0.84	.36		
	4	2	89	-1.59 (-2.16, -1.02)	18	1.21	.27		
Delivery format	Individual-based	9	511	-1.21 (-2.16, -0.27)	92	95.54	< .01	99.73	< .001
	Group-based	4	290	-0.33 (-0.56, -0.09)	6	3.18	.36		
	Mixed	1	144	-3.08 (-3.56, -2.59)	NA	NA	NA		
Face-to-face	Yes	11	754	-1.42 (-2.23, -0.60)	94	173.58	< .01	7.72	.01
	No	3	191	-0.19 (-0.48, 0.09)	0	1.15	.56		
Self-help	Yes	3	223	-0.37 (-0.56, -0.08)	0	0.66	.72	4.85	.03
	No	11	722	-1.37 (-2.21, -0.52)	95	183.17	< .01		
Active control	Yes [c]	8	657	-1.47 (-2.60, -0.33)	96	174.49	< .01	1.97	.16
	No [d]	6	288	-0.60 (-1.02, -0.18)	59	12.20	.03		

(continued)

© 2023 Ye F et al. *JAMA Network Open.*

eTable 6. Subgroup Analysis for Psychological Health Outcomes at Post-intervention (Continued)

Design	Subgroup	No. of studies	Sample size	SMD (95% CI)	Heterogeneity			Subgroup differences	
					I^2, %	Q	*P*-value	Q	*P*-value
**Anxiety symptoms**									
Chinese study	Yes	3	382	-1.10 (-1.99, -0.20)	95	38.65	< .01	0.02	.88
	No	10	503	-1.01 (-1.70, -0.31)	89	82.62	< .01		
Setting	Community [a]	10	642	-0.78 (-1.30, -0.26)	86	66.18	< .01	1.72	.19
	Hospital [b]	3	243	-1.86 (-3.39, -0.33)	93	26.56	< .01		
Risk of bias	Low	3	232	-0.36 (-0.65, -0.07)	0	1.63	.44	4.83	.09
	Some concerns	8	519	1.16 (-1.85, -0.47)	89	64.19	< .01		
	High	2	134	-1.37 (-4.05, 1.30)	98	39.63	< .01		
Health context for caregiving	Children with chronic conditions	8	687	-0.93 (-1.56, -0.30)	92	85.23	< .01	9.00	.01
	Adults with CNS diseases	3	108	-0.97 (-1.37, -0.56)	25	2.66	.26		
	Adults with life-limiting diseases	1	50	0.04 (-0.51, 0.60)	NA	NA	NA		
Intervention content	ACT	6	320	-0.95 (-1.94, 0.04)	90	48.69	< .01	0.06	.80
	ACT + disease/caregiving education	7	565	-1.10 (-1.75, -0.45)	91	66.94	< .01		
No. of ACT processes	6	8	652	-0.82 (-1.51, -0.14)	93	93.21	< .01	2.35	.31
	5	2	104	-0.64 (-1.04, -0.25)	0	0.05	.83		
	4	2	89	-1.11 (-1.57, -0.66)	0	0.05	.42		
Delivery format	Individual-based	8	429	-0.87 (-1.52, -0.22)	88	60.10	< .01	5.47	.06
	Group-based	4	312	-1.17 (-2.52, 0.17)	91	34.89	< .01		
	Mixed	1	144	-1.75 (-2.14, -1.37)	NA	NA	NA		
Face-to-face	Yes	10	694	-1.32 (-1.93, -0.72)	90	89.78	< .01	13.29	< .001
	No	3	191	-0.08 (-0.36, 0.21)	0	0.61	.74		
Self-help	Yes	2	141	-0.12 (-0.45, 0.21)	0	0.37	.54	9.48	.002
	No	11	744	-1.20 (-1.79, -0.60)	90	103.16	< .01		
Active control	Yes [c]	7	575	-1.06 (-1.77, -0.34)	92	76.72	< .01	0.01	.93
	No [d]	6	310	-1.00 (-1.93, -0.07)	88	41.60	< .01		

(continued)

© 2023 Ye F et al. *JAMA Network Open.*

eTable 6. Subgroup Analysis for Psychological Health Outcomes at Post-intervention (Continued)

Design	Subgroup	No. of studies	Sample size	SMD (95% CI)	Heterogeneity			Subgroup differences	
					*I*², %	Q	*P*-value	Q	*P*-value
**Stress symptoms**									
Chinese study	Yes	3	278	-0.44 (-0.68, -0.21)	0	1.5	.47	0.26	.61
	No	15	992	-0.59 (-1.12, -0.07)	84	102.02	< .01		
Setting	Community [a]	14	1046	-0.65 (-1.19, -0.10)	84	93.71	< .01	1.35	.24
	Hospital [b]	4	224	-0.23 (-0.69, 0.24)	65	8.66	.03		
Risk of bias	Low	6	432	-0.25 (-0.50, -0.002)	34	9.10	.17	2.11	.35
	Some concerns	7	362	-0.50 (-0.82, -0.17)	50	13.94	.05		
	High	5	476	-1.18 (-3.06, 0.70)	95	78.20	< .01		
Health context for caregiving	Children with chronic conditions	10	705	-0.89 (-1.76, -0.02)	90	87.57	< .01	2.32	.51
	Adults with CNS diseases	3	191	-0.23 (-0.52, 0.05)	0	0.89	.83		
	Adults with life-limiting diseases	4	348	-0.21 (-0.54, - 0.12)	53	8.42	.08		
	Adults with the psychotic disorders	1	26	-0.07 (-0.86, 0.72)	NA	NA	NA		
Intervention content	ACT	7	363	-0.26 (-0.50, -0.02)	0	6.97	.43	1.47	.23
	ACT + disease/caregiving education	11	907	-0.74 (-1.48, -0.0004)	88	94.34	< .01		
No. of ACT processes	6	11	694	-0.68 (-1.43, -0.06)	87	85.43	< .01	0.78	.68
	5	5	476	-0.45 (-0.72, -0.18)	49	9.75	.08		
	4	2	100	-0.12 (-1.13, 0.88)	84	6.09	.01		
Delivery format	Individual-based	12	892	-0.58 (-1.22, 0.06)	86	91.66	< .01	3.65	.16
	Group-based	5	338	-0.61 (-0.83, -0.39)	31	5.83	.21		
	Mixed	1	40	0.03 (-0.59, 0.65)	NA	NA	NA		
Face-to-face	Yes	10	675	-0.88 (-1.76, -0.003)	89	83.7	< .01	2.02	.16
	No	8	595	-0.23 (-0.44, -0.01)	31	13.12	.16		
Self-help	Yes	7	653	-0.21 (-0.39, -0.02)	23	10.45	.24	2.28	.13
	No	11	617	-0.84 (-1.64, -0.04)	88	82.30	< .01		
Active control	Yes [c]	9	584	-0.88 (-1.90, 0.14)	91	89.79	< .01	1.33	*.25*
	No [d]	9	686	-0.28 (-0.43, -0.12)	0	8.77	.55		

Abbreviations: CNS, central nervous system; NA, not applicable.
[a] Including online program and outpatient clinic.
[b] Including inpatient care-recipient.
[c] Including psycho-intervention, psycho-education, disease education.
[d] Including usual care and waitlist control.

© 2023 Ye F et al. *JAMA Network Open.*

eTable 7. Subgroup Analysis for Psychological Health Outcomes at Follow-Up

Design	Subgroup	No. of studies	Sample size	SMD (95% CI)	Heterogeneity			Subgroup differences	
					I^2, %	Q	*P*-value	Q	*P*-value
**Experiential avoidance**									
Chinese study	Yes	3	382	-1.48 (-1.83, -1.13)	56	4.52	.10	0.64	.42
	No	12	597	-1.10 (-1.96, -0.24)	89	111.28	< .01		
Setting	Community [a]	11	670	-1.11 (-2.02, -0.19)	90	106.55	< .01	0.21	.65
	Hospital [b]	4	309	-1.38 (-2.08, -0.67)	85	19.72	< .01		
Risk of bias	Low	5	419	-0.69 (-1.22, -0.17)	81	20.50	< .01	1.53	.46
	Some concerns	6	328	-1.00 (-1.61, -0.40)	85	38.79	< .01		
	High	4	232	-2.22 (-4.96, 0.52)	96	77.6	< .01		
Health context for caregiving	Children with chronic conditions	6	539	-1.93 (-3.66, -0.19)	94	85.83	< .01	3.91	.27
	Adults with CNS diseases	3	90	-0.84 (-1.28, -0.40)	0	0.66	.88		
	Adults with life-limiting diseases	4	282	-0.31 (-0.91, 0.29)	85	19.78	< .01		
	Adults with the psychotic disorders	1	28	-0.74 (-1.52, 0.04)	NA	NA	NA		
Intervention content	ACT	6	263	-0.77 (-1.30, -0.24)	71	21.01	< .01	1.09	.30
	ACT + disease/caregiving education	9	716	-1.46 (-2.66, -0.27)	92	104.69	< .01		
No. of ACT processes	6	10	672	-1.28 (-2.30, -0.27)	92	126.52	< .01	4.13	.13
	5	2	165	-1.02 (-1.35, -0.69)	0	0.60	.44		
	4	2	102	-0.53 (-0.93, -0.13)	0	0.89	.35		
Delivery format	Individual-based	9	536	-1.29 (-2.38, -0.19)	91	98.00	< .01	0.18	.92
	Group-based	4	259	-1.15 (-1.70, -0.60)	60	7.52	.06		
	Mixed	2	184	-0.81 (-2.75, 1.12)	96	27.97	< .01		
Face-to-face	Yes	9	599	-1.71 (-2.82, -0.59)	90	78.40	<.01	4.34	.04
	No	6	380	-0.44 (-0.86, -0.02)	72	21.76	< .01		
Self-help	Yes	4	290	-0.71 (-1.12, -0.30)	53	8.43	.08	1.41	.23
	No	11	689	-1.36 (-2.36, -0.36)	92	123.97	< .01		
Active control	Yes [c]	10	676	-1.27 (-2.38, -0.15)	93	125.38	< .01	0.17	.68
	No [d]	5	303	-1.01 (-1.50, -0.52)	63	13.65	.02		

(continued)

© 2023 Ye F et al. *JAMA Network Open.*

eTable 7. Subgroup Analysis for Psychological Health Outcomes at Follow-Up (Continued)

Design	Subgroup	No. of studies	Sample size	SMD (95% CI)	Heterogeneity			Subgroup differences	
					I^2, %	Q	*P*-value	Q	*P*-value
**Anxiety symptoms**									
Chinese study	Yes	3	396	-1.04 (-1.76, -0.32)	91	21.58	< .01	0.02	.89
	No	7	312	-1.12 (-1.76, -0.32)	91	65.05	< .01		
Setting	Community [a]	7	440	-0.78 (-1.47, -0.01)	86	41.85	< .01	1.69	.19
	Hospital [b]	3	268	-1.82 (-3.21, -0.42)	93	27.99	< .01		
Risk of bias	Low	2	60	-0.40 (-1.16, 0.37)	64	2.79	.09	20.51	< .001
	Some concerns	7	461	-1.08 (-1.82, -0.34)	89	55.01	< .01		
	High	1	60	-2.73 (-3.44, -2.01)	NA	NA	NA		
Health context for caregiving	Children with chronic conditions	5	530	-1.28 (-2.06, -0.50)	91	44.94	< .01	7.84	.02
	Adults with CNS diseases	3	88	-0.51 (-1.02, -0.002)	31	2.88	.24		
	Adults with life-limiting diseases	1	50	0.07 (-0.48, 0.63)	NA	NA	NA		
Intervention content	ACT	5	233	-1.04 (-2.08, 0.004)	88	33.41	< .01	0.03	.87
	ACT + disease/caregiving education	5	475	-1.15 (-2.09, -0.22)	92	49.30	< .01		
No. of ACT processes	6	7	599	-0.91 (-1.64, -0.19)	91	65.06	< .01	0.18	.67
	4	2	69	0.72 (-1.22, -0.22)	0	0.29	.59		
Delivery format	Individual-based	7	356	-0.78 (-1.47, -0.09)	86	41.92	< .01	6.19	.045
	Group-based	2	208	-1.89 (-4.33, 0.55)	96	23.42	< .01		
	Mixed	1	144	-1.77 (-2.16, -1.39)	NA	NA	NA		
Face-to-face	Yes	8	584	-1.29 (-2.06, -0.53)	90	69.85	< .01	3.10	.08
	No	2	124	-0.33 (-1.09, 0.43)	77	4.35	.04		
Self-help	Yes	1	74	-0.70 (-1.17, -0.23)	NA	NA	NA	0.96	.33
	No	9	634	-1.14 (-1.87, -0.40)	91	85.68	< .01		
Active control	Yes [c]	7	572	-0.91 (-1.64, -0.17)	91	65.12	< .01	0.61	.44
	No [d]	3	136	-1.58 (-3.11, -0.05)	90	20.88	< .01		

(continued)

© 2023 Ye F et al. *JAMA Network Open.*

eTable 7. Subgroup Analysis for Psychological Health Outcomes at Follow-Up (Continued)

Design	Subgroup	No. of studies	Sample size	SMD (95% CI)	Heterogeneity			Subgroup differences	
					I^2, %	Q	*P*-value	Q	*P*-value
**Stress symptoms**									
Chinese study	Yes	2	238	-0.78 (-1.25, -0.31)	61	2.58	.11	0.03	.87
	No	10	636	-0.87 (-1.80, 0.07)	89	91.59	< .01		
Setting	Community [a]	10	749	-0.89 (-1.82, 0.03)	89	91.95	< .01	0.10	.75
	Hospital [b]	2	125	-0.70 (-1.43, 0.03)	74	3.86	.05		
Risk of bias	Low	5	419	-0.35 (-0.58, -0.13)	19	4.91	.30	1.87	.39
	Some concerns	2	74	-0.57 (-1.29, 0.16)	32	2.93	.23		
	High	5	381	-1.63 (-3.61, 0.34)	95	83.83	< .01		
Health context for caregiving	Children with chronic conditions	4	372	-2.03 (-4.40, 0.35)	96	70.11	< .01	2.60	.46
	Adults with CNS diseases	3	191	-2.19 (-0.51, 0.07)	35	4.63	.20		
	Adults with life-limiting diseases	4	282	-0.26 (-0.49, -0.02)	0	0.84	.84		
	Adults with the psychotic disorders	1	29	-0.50 (-1.26, 0.25)	NA	NA	NA		
Intervention content	ACT	4	177	-0.61 (-1.01, -0.22)	17	4.84	.30	0.25	.61
	ACT + disease/caregiving education	8	697	-0.96 (-2.25, 0.33)	92	91.99	< .01		
No. of ACT processes	6	8	505	-1.11 (-2.25, 0.02)	91	87.43	< .01	2.02	.36
	5	3	314	-0.27 (-0.50, -0.05)	0	0.82	.66		
	4	1	55	-0.32 (-0.87, 0.24)	NA	NA	NA		
Delivery format	Individual-based	9	637	-1.01 (-2.03, 0.004)	91	94.88	< .01	3.24	.20
	Group-based	2	197	-0.57 (-0.85, -0.28)	0	0.03	.85		
	Mixed	1	40	-0.05 (-0.67, 0.57)	NA	NA	NA		
Face-to-face	Yes	6	494	-1.27 (-3.00, 0.46)	94.3	87.52	< .01	0.91	.34
	No	6	380	-0.42 (-0.67, -0.17)	24	7.94	.24		
Self-help	Yes	5	438	-0.45 (-0.72, -0.17)	35	7.71	.17	0.67	.41
	No	7	436	-1.08 (-2.58, 0.41)	93	86.35	< .01		
Active control	Yes [c]	7	462	-1.06 (-2.55, 0.44)	93	87.50	< .01	0.53	.47
	No [d]	5	412	-0.49 (-0.79, -0.19)	35	7.69	.17		

Abbreviations: NA, not applicable.

[a] Including online program and outpatient clinic.

[b] Including inpatient care-recipient.

[c] Including psycho-intervention, psycho-education, disease education.

[d] Including usual care and waitlist control.

© 2023 Ye F et al. *JAMA Network Open.*

eTable 8. Summary of Univariable Metaregression Analyses

Moderators	Post-intervention		Follow-up	
	β (SE) [95% CI]	*P*-value	β (SE) [95% CI]	*P*-value
Experiential avoidance				
Sample size [a]	-0.002 (0.008) [-0.018, 0.014]	.80	-0.002 (0.008) [-0.018, 0.013]	.78
Mean age [b]	0.052 (0.025) [0.003, 0.100]	.04	0.041 (0.017) [0.008, 0.075]	.02
Intervention duration	-0.183 (0.068) [-0.316, -0.050]	.01	-0.023 (0.138) [-0.294, 0.247]	.87
No. of sessions	-0.247 (0.064) [-0.373, -0.121]	<.001	-0.047 (0.159) [-0.358, 0.264]	.77
Time of therapeutic encounter [c]	-0.002 (0.001) [-0.004, -0.001]	.01	-0.0003 (0.004) [-0.007, 0.007]	.93
Attrition rate [d]	5.100 (1.660) [1.848, 8.353]	.002	4.393 (2.349) [0.727, 8.058]	.02
Depressive symptoms				
Sample size [a]	-0.002 (0.009) [-0.019, 0.015]	.79	NA	NA
Mean age [b]	0.002 (0.029) [-0.055, 0.060]	.94	NA	NA
Intervention duration	0.033 (0.116) [-0.194, 0.259]	.78	NA	NA
No. of sessions	-0.168 (0.195) [-0.550, 0.214]	.39	NA	NA
Time of therapeutic encounter [c]	-0.0004 (0.002) [-0.004, 0.003]	.84	NA	NA
Attrition rate [d]	2.342 (2.541) [-2.639, 7.323]	.36	NA	NA
Anxiety symptoms				
Sample size [a]	0.003 (0.007) [-0.012, 0.017]	.73	-0.000 (0.008) [-0.016, 0.016]	> .99
Mean age [b]	0.030 (0.026) [-0.021, 0.081]	.25	0.051 (0.025) [0.001, 0.101]	.04
Intervention duration	-0.043 (0.113) [-0.264, 0.178]	.70	-0.134 (0.211) [-0.548, 0.281]	.53
No. of sessions	-0.339 (0.148) [-0.629, -0.049]	.02	-0.230 (0.210) [-0.642, 0.182]	.27
Time of therapeutic encounter [c]	-0.001 (0.001) [-0.003, 0.002]	.63	-0.001 (0.003) [-0.006, 0.004]	.63
Attrition rate [d]	2.737 (1.899) [-0.985, 6.459]	.15	2.998 (2.022) [-0.967, 6.962]	.14
Stress symptoms				
Sample size [a]	0.003 (0.005) [-0.008, 0.013]	.64	0.003 (0.009) [-0.014, 0.020]	.75
Mean age [b]	0.015 (0.008) [-0.000, 0.030]	.05	0.020 (0.009) [0.004, 0.038]	.02
Intervention duration	-0.011 (0.048) [-0.104, 0.083]	.82	0.094 (0.159) [-0.218, 0.405]	.55
No. of sessions	-0.034 (0.054) [-0.139, 0.071]	.52	0.066 (0.249) [-0.422, 0.555]	.79
Time of therapeutic encounter [c]	-0.0002 (0.001) [-0.002, -0.001]	.77	-0.001 (0.002) [-0.006, 0.004]	.65
Attrition rate [d]	3.106 (1.390) [0.382, 5.829]	.03	3.514 (2.483) [-1.353, 8.381]	.16

SE, standard error; β, coefficient; [a] the total allocation sample size of the intervention group and control group; [b] the mean age of informal caregivers; [c] facilitator-participant interaction and contact time (median, 390 mins; range, 180-2160 mins) through in-person/virtual face-to-face or telephone call, excluding the self-help studies to make this comparable; [d] the attrition rate of the whole study, including the intervention group and control group.

© 2023 Ye F et al. *JAMA Network Open.*

eFigure 1. Map of Included Studies by Country

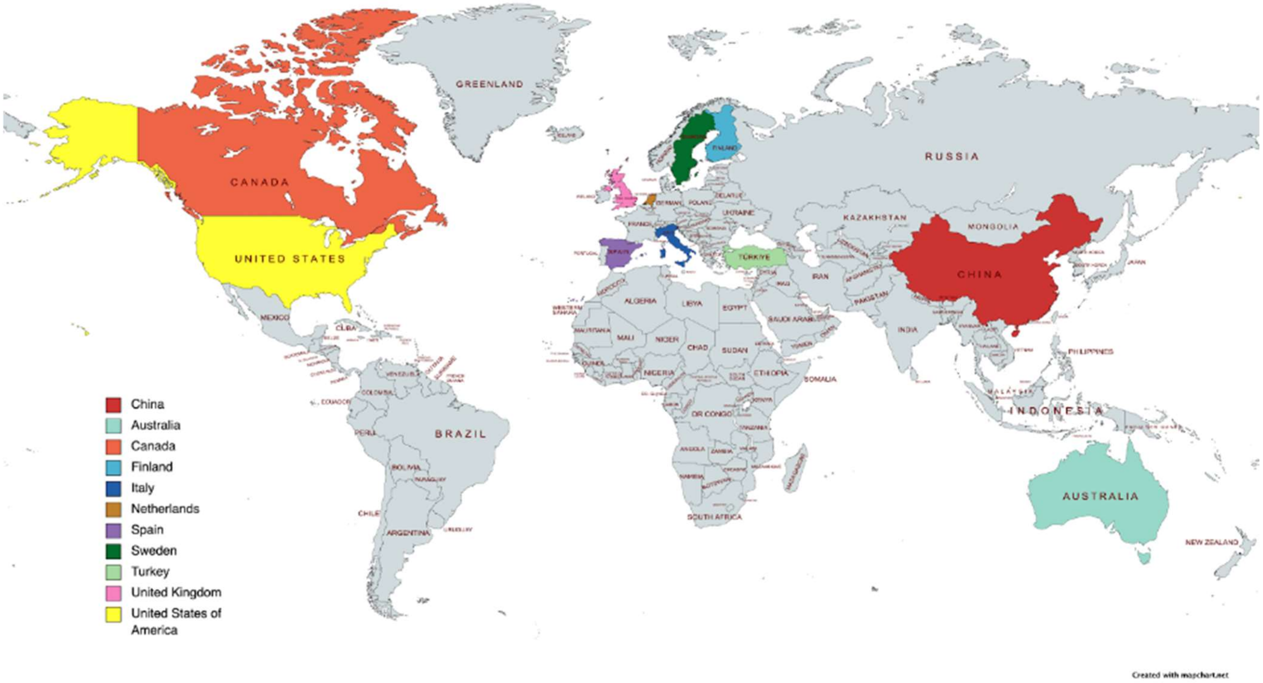

eFigure 2. Risk of Bias of Included Studies

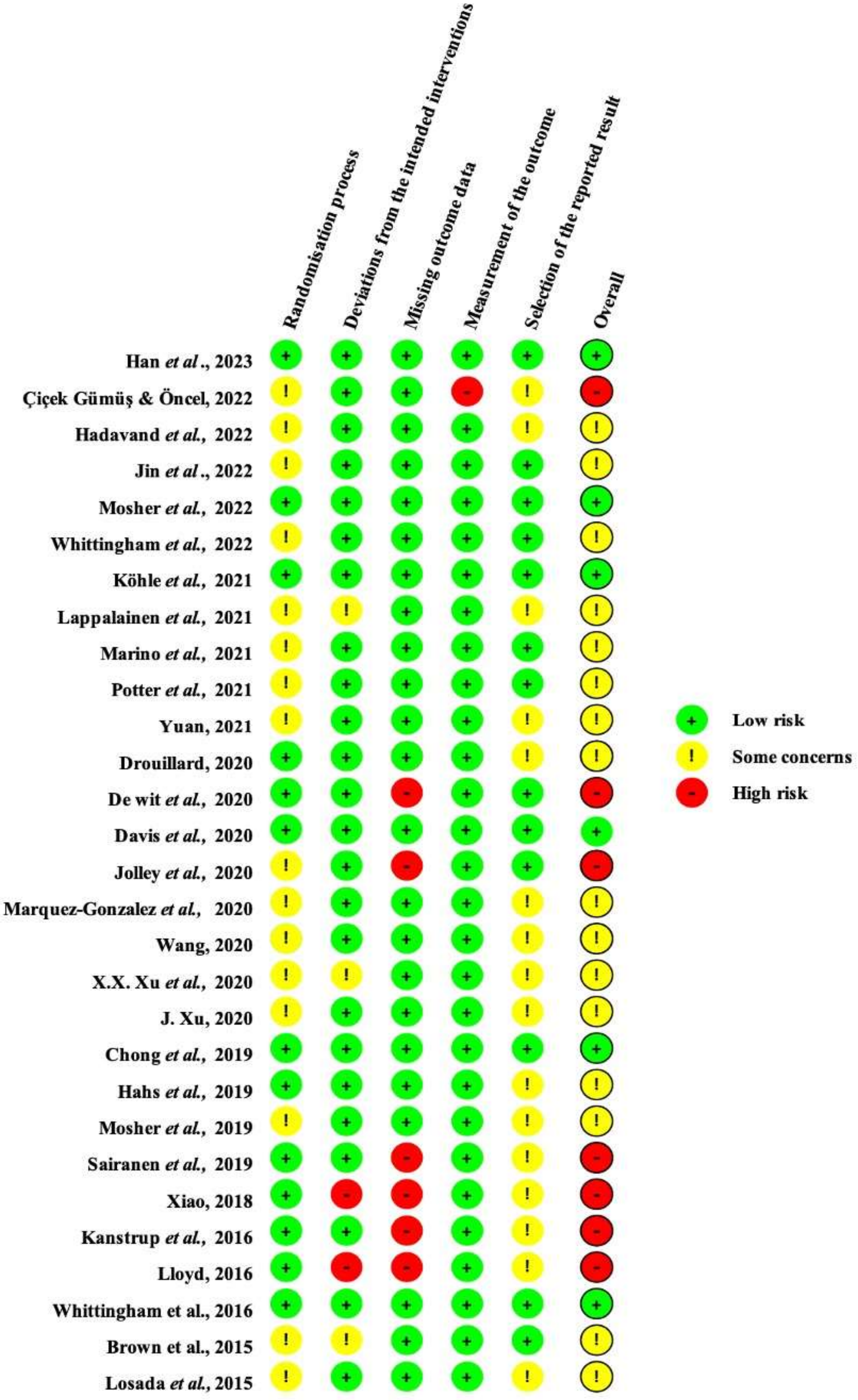

eFigure 3. Forest Plots for Experimental Avoidance at Postintervention and 1- to 3-Month and 4- to 6-Month Follow-Ups

Study	Intervention group Total	Intervention group Mean	Intervention group SD	Control group Total	Control group Mean	Control group SD	SMD	95%-CI	Weight
**Post–intervention**									
Lloyd, 2016	5	2.80	3.89	5	−3.20	4.55	1.28	[−0.15; 2.71]	3.4%
Mosher et al., 2022	20	−1.68	5.58	20	−3.45	7.18	0.27	[−0.35; 0.89]	4.1%
Whittingham et al., 2022	37	2.00	7.66	30	0.96	8.20	0.13	[−0.35; 0.61]	4.2%
Mosher et al., 2019	25	−0.32	2.66	25	−0.22	2.68	−0.04	[−0.59; 0.52]	4.2%
Drouillard, 2020	13	−0.14	8.68	10	0.87	7.60	−0.12	[−0.94; 0.71]	4.0%
Davis et al., 2020	35	−0.38	8.01	20	1.13	6.78	−0.20	[−0.75; 0.35]	4.2%
Sairanen et al., 2019	37	−2.86	7.24	37	−1.19	8.28	−0.21	[−0.67; 0.24]	4.2%
Han et al., 2023	9	−4.66	7.58	10	−1.50	9.03	−0.36	[−1.27; 0.55]	3.9%
Köhle et al., 2021 (Online + AF)	70	0.00	0.65	33	0.30	0.63	−0.46	[−0.88; −0.04]	4.2%
Potter et al., 2021 (Email)	9	−0.62	6.88	4	2.87	4.91	−0.51	[−1.71; 0.69]	3.6%
Jin et al., 2022	20	−6.02	5.77	20	−1.61	8.72	−0.58	[−1.22; 0.05]	4.1%
Jolley et al., 2020	14	−2.50	7.09	10	2.00	7.26	−0.61	[−1.44; 0.23]	4.0%
Köhle et al., 2021 (Online + PF)	67	−0.10	0.64	33	0.30	0.63	−0.62	[−1.05; −0.20]	4.2%
Potter et al., 2021 (Email + phone)	7	−1.91	6.11	4	2.87	4.91	−0.76	[−2.05; 0.53]	3.6%
Brown et al., 2015	30	−9.50	15.21	29	1.53	13.09	−0.77	[−1.30; −0.24]	4.2%
Chong et al., 2019	84	−4.77	5.84	84	0.56	7.15	−0.81	[−1.13; −0.50]	4.3%
Xiao, 2018	34	−2.42	2.87	36	0.22	3.22	−0.85	[−1.35; −0.36]	4.2%
Hahs et al., 2019	9	−2.45	3.81	9	1.23	4.27	−0.87	[−1.84; 0.11]	3.8%
Losada et al., 2015	33	−6.70	6.53	31	−0.05	6.52	−1.01	[−1.53; −0.48]	4.2%
Yuan, 2021	35	−14.17	7.57	35	−2.05	8.80	−1.46	[−1.99; −0.93]	4.2%
Wang, 2020	72	−7.33	3.42	72	−0.82	3.37	−1.91	[−2.30; −1.51]	4.2%
Hadavand et al., 2022	20	−5.20	3.56	20	2.35	3.40	−2.12	[−2.91; −1.34]	4.0%
J. Xu, 2020	30	−7.40	3.04	30	−1.60	2.06	−2.20	[−2.85; −1.55]	4.1%
Marino et al., 2021	20	−22.30	4.77	20	−0.25	1.25	−6.20	[−7.75; −4.64]	3.3%
Çiçek Gümü.. & Öncel, 2022	30	−28.16	4.76	30	−0.83	3.83	−6.25	[−7.51; −4.99]	3.6%
**Random effects model**	**765**			**657**			**−1.04**	**[−1.65; −0.44]**	**100.0%**
**Prediction interval**	.			.				**[−4.19; 2.10]**	—
Heterogeneity: $I^2 = 90\%$ [87%; 93%], $\chi^2_{24} = 243.05$ ($p < 0.001$)									
**1– to 3–month follow–up**									
Mosher et al., 2022	20	−1.87	5.54	20	−3.11	7.08	0.19	[−0.43; 0.81]	12.8%
Mosher et al., 2019	25	−0.67	2.66	25	−0.99	2.68	0.12	[−0.44; 0.67]	13.0%
Drouillard, 2020	13	−4.13	8.04	10	0.12	6.85	−0.54	[−1.38; 0.30]	12.3%
Jolley et al., 2020	16	−3.60	6.97	12	1.90	7.53	−0.74	[−1.52; 0.04]	12.5%
Han et al., 2023	9	−7.66	7.57	10	−0.10	9.03	−0.86	[−1.81; 0.09]	12.1%
Xiao, 2018	34	−4.48	2.90	36	0.00	3.22	−1.44	[−1.97; −0.91]	13.0%
Wang, 2020	72	−6.59	3.21	72	−0.45	3.62	−1.79	[−2.17; −1.40]	13.2%
Çiçek Gümü.. & Öncel, 2022	30	−28.13	4.78	30	0.37	3.83	−6.50	[−7.80; −5.19]	11.0%
**Random effects model**	**219**			**215**			**−1.40**	**[−2.82; 0.02]**	**100.0%**
**Prediction interval**	.			.				**[−6.63; 3.83]**	—
Heterogeneity: $I^2 = 94\%$ [90%; 96%], $\chi^2_{7} = 117.38$ ($p < 0.001$)									
**4– to 6–month follow–up**									
Davis et al., 2020	35	−0.98	8.17	20	1.85	7.68	−0.35	[−0.90; 0.20]	13.0%
Sairanen et al., 2019	37	−4.36	7.11	37	−1.39	8.16	−0.38	[−0.84; 0.08]	13.1%
Losada et al., 2015	25	−5.15	6.47	22	−0.30	6.44	−0.74	[−1.33; −0.14]	12.9%
Potter et al., 2021 (Email + phone)	7	−5.11	7.82	4	1.12	5.34	−0.80	[−2.10; 0.49]	11.0%
Köhle et al., 2021 (Online + PF vs. Online +AF)	67	−0.30	0.64	70	0.40	0.65	−1.08	[−1.44; −0.72]	13.3%
Chong et al., 2019	84	−6.23	5.84	84	1.54	6.68	−1.23	[−1.56; −0.90]	13.3%
Potter et al., 2021 (Email)	9	−8.19	6.85	4	1.12	5.34	−1.34	[−2.66; −0.01]	10.9%
Hadavand et al., 2022	20	−4.35	3.71	20	2.75	3.38	−1.96	[−2.73; −1.20]	12.5%
**Random effects model**	**284**			**261**			**−0.95**	**[−1.32; −0.57]**	**100.0%**
**Prediction interval**	.			.				**[−2.08; 0.19]**	—
Heterogeneity: $I^2 = 67\%$ [30%; 84%], $\chi^2_{7} = 21.21$ ($p = 0.003$)									

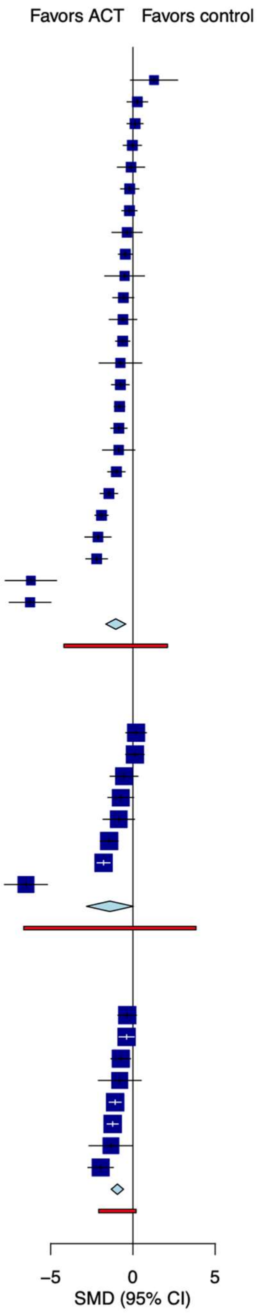

Heterogeneity: $I^2 = 90\%$ [87%; 92%], $\chi^2_{40} = 387.37$ ($p < 0.001$)
Test for subgroup differences: $p = 0.815$

eFigure 4. Forest Plots for Value-Based Living at Postintervention and 1- to 3-Month and 4- to 6-Month Follow-Ups

Study	Intervention group Total	Intervention group Mean	Intervention group SD	Control group Total	Control group Mean	Control group SD	SMD	95%–CI	Weight
**Post–intervention**									
Marino, 2021	20	21.30	6.79	20	−0.10	1.30	4.29	[3.12; 5.46]	9.1%
Potter, 2021 (Email + phone)	7	7.06	5.84	4	−1.38	6.32	1.28	[−0.11; 2.68]	8.1%
Hahs, 2019	9	3.44	3.89	9	−0.56	2.05	1.23	[0.20; 2.25]	9.7%
Han et al., 2023	9	5.33	5.46	10	0.10	6.57	0.82	[−0.12; 1.77]	10.1%
Lappalainen, 2021	40	5.14	5.15	42	1.27	4.62	0.78	[0.33; 1.23]	12.1%
Jolley, 2020	15	3.20	5.95	10	−2.00	7.24	0.78	[−0.06; 1.61]	10.6%
Potter, 2021 (Email)	9	5.13	9.30	4	−1.38	6.32	0.70	[−0.52; 1.93]	8.8%
Lloyd, 2016	5	−0.80	12.46	5	−6.80	9.80	0.48	[−0.78; 1.75]	8.6%
Davis, 2020	32	7.97	16.27	15	7.69	16.05	0.02	[−0.60; 0.63]	11.5%
Mosher, 2022	20	0.95	4.00	20	1.39	3.77	−0.11	[−0.73; 0.51]	11.5%
**Random effects model**	**166**			**139**			**0.98**	**[0.24; 1.71]**	**100.0%**
**Prediction interval**	.			.				**[−1.65; 3.60]**	**--**
Heterogeneity: $I^2 = 82\%$ [68%; 90%], $\chi^2_9 = 49.78$ ($p < 0.001$)									
**1– to 3–month follow–up**									
Jolley, 2020	17	3.60	6.09	12	−0.10	7.35	0.54	[−0.21; 1.30]	33.6%
Han et al., 2023	9	6.44	5.55	10	3.00	6.61	0.54	[−0.38; 1.46]	31.3%
Mosher, 2022	20	0.87	3.55	20	2.69	3.74	−0.49	[−1.12; 0.14]	35.1%
**Random effects model**	**46**			**42**			**0.15**	**[−0.57; 0.86]**	**100.0%**
**Prediction interval**	.			.				**[−7.69; 7.99]**	**--**
Heterogeneity: $I^2 = 64\%$ [0%; 90%], $\chi^2_2 = 5.52$ ($p = 0.063$)									
**4– to 6–month follow–up**									
Potter, 2021 (Email + phone)	7	10.66	5.83	4	−1.63	7.38	1.76	[0.23; 3.28]	27.0%
Potter, 2021 (Email)	9	3.14	5.87	4	−1.63	7.38	0.70	[−0.52; 1.92]	31.7%
Davis, 2020	32	10.77	15.67	15	15.29	15.40	−0.29	[−0.90; 0.33]	41.3%
**Random effects model**	**48**			**23**			**0.56**	**[−0.60; 1.72]**	**100.0%**
**Prediction interval**	.			.				**[−12.65; 13.77]**	**--**
Heterogeneity: $I^2 = 71\%$ [3%; 92%], $\chi^2_2 = 6.97$ ($p = 0.031$)									

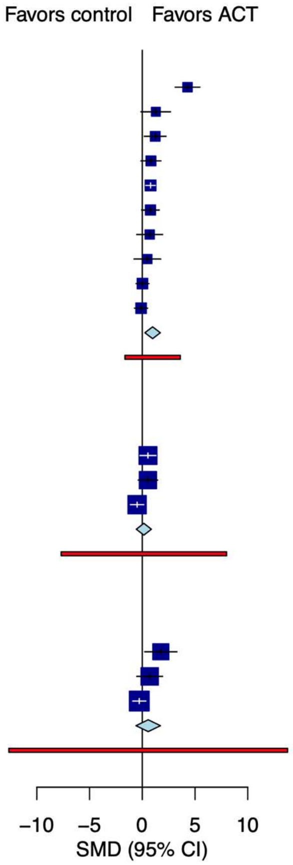

Heterogeneity: $I^2 = 79\%$ [66%; 87%], $\chi^2_{15} = 70.76$ ($p < 0.001$)
Test for subgroup differences: $p = 0.284$

eFigure 5. Forest Plots for Mindfulness at Postintervention and 1- to 3-Month and 4- to 6-Month Follow-Ups

Study	Intervention group Total	Intervention group Mean	Intervention group SD	Control group Total	Control group Mean	Control group SD	SMD	95%–CI	Weight
**Post–intervention**									
Marino et al, 2021	20	22.80	4.38	20	0.15	1.53	6.77	[5.09; 8.44]	10.4%
Potter et al, 2021 (Email + phone)	7	3.43	5.50	4	−2.00	4.25	0.97	[−0.36; 2.30]	11.5%
Hahs et al, 2019	9	6.34	8.20	9	−1.11	9.58	0.80	[−0.17; 1.76]	12.5%
Lappalainen et al, 2021	40	12.35	13.32	42	3.10	10.79	0.76	[0.31; 1.21]	13.6%
Sairanen et al, 2019	37	14.44	15.62	37	8.06	22.87	0.32	[−0.14; 0.78]	13.6%
Whittingham et al, 2022	37	0.74	3.34	30	−0.31	3.57	0.30	[−0.18; 0.79]	13.5%
Potter et al, 2021 (Email)	9	−0.47	4.95	4	−2.00	4.25	0.30	[−0.89; 1.48]	11.9%
Jolley et al, 2020	15	3.80	11.87	10	4.00	10.33	−0.02	[−0.82; 0.78]	12.9%
**Random effects model**	**174**			**156**			**1.19**	**[−0.23; 2.61]**	**100.0%**
**Prediction interval**	**.**			**.**				**[−3.99; 6.37]**	**---**
Heterogeneity: $I^2 = 88\%$ [79%; 93%], $\chi^2_7 = 58.29$ ($p < 0.001$)									
**4– to 6–month follow–up**									
Sairanen et al, 2019	37	19.71	16.47	37	−0.69	15.42	1.27	[0.76; 1.77]	36.9%
Potter et al, 2021 (Email)	9	5.53	3.97	4	0.38	4.58	1.16	[−0.13; 2.45]	31.8%
Potter et al, 2021 (Email + phone)	7	6.23	5.30	4	0.38	4.58	1.05	[−0.29; 2.40]	31.3%
**Random effects model**	**53**			**45**			**1.23**	**[0.79; 1.67]**	**100.0%**
**Prediction interval**	**.**			**.**				**[−1.63; 4.09]**	**---**
Heterogeneity: $I^2 = 0\%$ [0%; 90%], $\chi^2_2 = 0.1$ ($p = 0.952$)									
**1– to 3–month follow–up**									
Jolley et al, 2020	16	8.00	11.98	11	5.80	9.54	0.19	[−0.58; 0.96]	100.0%

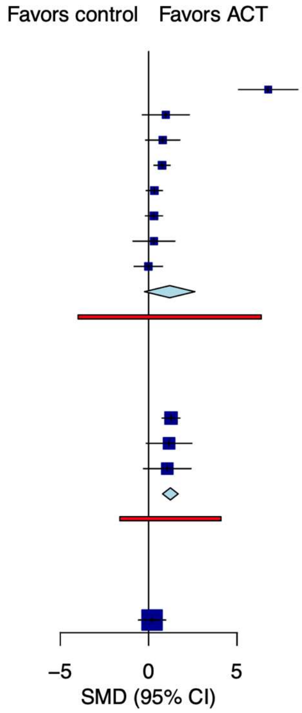

Heterogeneity: $I^2 = 83\%$ [73%; 90%], $\chi^2_{11} = 66.59$ ($p < 0.001$)
Test for subgroup differences: $p = 0.070$

eFigure 6. Forest Plots for Cognitive Confusion at Postintervention and 1- to 3-Month and 4- to 6-Month Follow-Ups

Study	Intervention group Total	Intervention group Mean	Intervention group SD	Control group Total	Control group Mean	Control group SD	SMD	95%–CI	Weight
**Post–intervention**									
Sairanen, 2019	37	−7.20	10.96	37	−7.32	11.92	0.01	[−0.45; 0.47]	22.7%
Drouillard, 2020	13	−3.50	9.09	10	−0.34	7.61	−0.36	[−1.19; 0.47]	13.2%
Han et al., 2023	9	−3.11	8.22	10	0.10	8.16	−0.37	[−1.28; 0.54]	11.9%
J. Xu, 2020	30	−5.93	5.27	30	−2.40	4.53	−0.71	[−1.23; −0.19]	20.7%
Hahs, 2019	9	−4.11	8.14	9	3.78	11.23	−0.77	[−1.73; 0.20]	11.0%
Yuan, 2021	35	−19.62	12.48	35	−2.26	12.24	−1.39	[−1.91; −0.86]	20.6%
**Random effects model**	**133**			**131**			**−0.61**	**[−1.06; −0.15]**	**100.0%**
**Prediction interval**	**.**			**.**				**[−2.00; 0.79]**	**--**
Heterogeneity: $I^2 = 70\%$ [28%; 87%], $\chi^2_5 = 16.41$ ($p = 0.006$)									
**1– to 3–month follow–up**									
Han et al., 2023	9	−3.67	8.28	10	−1.60	8.28	−0.24	[−1.14; 0.67]	48.7%
Drouillard, 2020	13	−6.72	8.61	10	0.37	7.23	−0.85	[−1.72; 0.02]	51.3%
**Random effects model**	**22**			**20**			**−0.56**	**[−1.18; 0.07]**	**100.0%**
Heterogeneity: $I^2 = 0\%$, $\chi^2_1 = 0.91$ ($p = 0.340$)									
**4– to 6–month follow–up**									
Sairanen, 2019	37	−9.78	10.37	37	−1.40	11.79	−0.75	[−1.22; −0.27]	100.0%

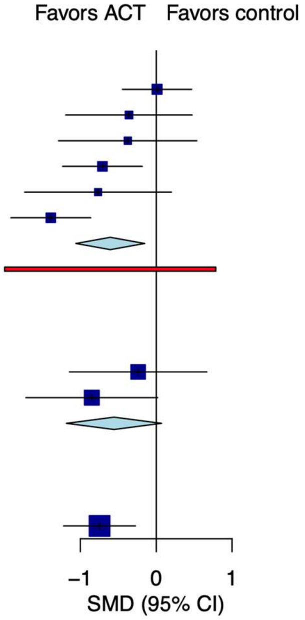

Heterogeneity: $I^2 = 55\%$ [4%; 79%], $\chi^2_8 = 17.68$ ($p = 0.024$)
Test for subgroup differences: $p = 0.868$

eFigure 7. Forest Plots for Depressive Symptoms at Postintervention and 1- to 3-Month and 4- to 6-Month Follow-Ups

Study	Intervention group Total	Intervention group Mean	Intervention group SD	Control group Total	Control group Mean	Control group SD	SMD	95%–CI	Weight
**Post–intervention**									
Mosher et al., 2019	25	−0.84	2.33	25	−1.00	2.40	0.07	[−0.49; 0.62]	7.3%
Chong et al., 2019	84	−2.09	4.87	84	−1.14	4.37	−0.20	[−0.51; 0.10]	7.5%
Brown et al., 2015	30	−2.93	6.89	29	−0.94	7.70	−0.27	[−0.78; 0.24]	7.3%
Whittingham et al., 2022	37	−0.25	2.69	30	0.74	4.20	−0.28	[−0.77; 0.20]	7.3%
Sairanen et al., 2019	37	−4.31	7.43	37	−2.27	6.51	−0.29	[−0.75; 0.17]	7.4%
Lappalainen et al., 2021	40	−3.00	3.91	42	−1.06	3.56	−0.51	[−0.96; −0.07]	7.4%
Han et al., 2023	9	−2.78	5.38	10	−0.50	2.13	−0.54	[−1.46; 0.38]	6.8%
Whittingham et al., 2016	23	−3.31	3.90	22	0.45	7.26	−0.64	[−1.24; −0.04]	7.2%
Hahs et al., 2019	9	−4.22	4.09	9	−0.45	3.55	−0.94	[−1.92; 0.05]	6.7%
Yuan, 2021	35	−11.71	8.44	35	−0.86	6.83	−1.40	[−1.92; −0.87]	7.3%
Losada et al., 2015	33	−13.35	7.37	31	−2.87	7.32	−1.41	[−1.96; −0.86]	7.3%
Marquez–Gonzalez et al., 2020	16	−14.84	7.22	9	0.74	7.45	−2.06	[−3.09; −1.04]	6.6%
Wang, 2020	72	−15.99	4.95	72	−0.61	5.00	−3.08	[−3.56; −2.59]	7.3%
Çiçek Gümü.. & Öncel, 2022	30	−12.66	2.66	30	−0.63	2.20	−4.86	[−5.89; −3.83]	6.6%
**Random effects model**	**480**			**465**			**−1.14**	**[−1.83; −0.45]**	**100.0%**
**Prediction interval**	**.**			**.**				**[−4.00; 1.72]**	**--**
Heterogeneity: $I^2 = 93\%$ [91%; 95%], $\chi^2_{13} = 198.96$ ($p < 0.001$)									
**4– to 6–month follow–up**									
Chong et al., 2019	84	−2.36	4.96	84	−0.16	4.32	−0.47	[−0.78; −0.16]	26.0%
Losada et al., 2015	25	−6.31	7.27	22	−2.83	7.23	−0.47	[−1.05; 0.11]	25.2%
Sairanen et al., 2019	37	−6.41	7.52	37	2.91	9.31	−1.09	[−1.58; −0.60]	25.5%
Marquez–Gonzalez et al., 2020	7	−11.02	10.01	15	−1.53	7.04	−1.13	[−2.10; −0.16]	23.3%
**Random effects model**	**153**			**158**			**−0.71**	**[−1.08; −0.35]**	**100.0%**
**Prediction interval**	**.**			**.**				**[−2.07; 0.64]**	**--**
Heterogeneity: $I^2 = 48\%$ [0%; 83%], $\chi^2_3 = 5.72$ ($p = 0.126$)									
**1– to 3–month follow–up**									
Mosher et al., 2019	25	−0.69	2.33	25	−0.86	2.40	0.07	[−0.48; 0.63]	20.6%
Han et al., 2023	9	−1.67	4.95	10	−1.20	2.78	−0.11	[−1.02; 0.79]	19.2%
X. X. Xu et al., 2020	41	−6.21	5.49	43	−3.11	6.08	−0.53	[−0.97; −0.09]	20.9%
Wang, 2020	72	−17.15	5.17	72	−1.27	4.93	−3.13	[−3.62; −2.64]	20.7%
Çiçek Gümü.. & Öncel, 2022	30	−12.13	2.67	30	0.17	2.23	−4.93	[−5.97; −3.89]	18.6%
**Random effects model**	**177**			**180**			**−1.71**	**[−3.62; 0.20]**	**100.0%**
**Prediction interval**	**.**			**.**				**[−9.21; 5.79]**	**--**
Heterogeneity: $I^2 = 97\%$ [95%; 98%], $\chi^2_4 = 141.75$ ($p < 0.001$)									

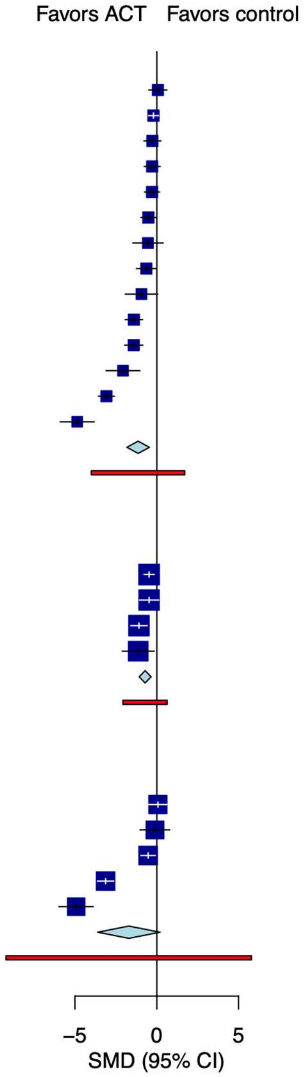

Heterogeneity: $I^2 = 94\%$ [92%; 95%], $\chi^2_{22} = 364.59$ ($p < 0.001$)
Test for subgroup differences: $p = 0.372$

eFigure 8. Forest Plots for Anxiety Symptoms at Postintervention and 1- to 3-Month and 4- to 6-Month Follow-Ups

Study	Intervention group Total	Intervention group Mean	Intervention group SD	Control group Total	Control group Mean	Control group SD	SMD	95%–CI	Weight
**Post–intervention**									
Mosher et al., 2019	25	−0.37	2.60	25	−0.48	2.67	0.04	[−0.51; 0.60]	7.9%
Sairanen et al., 2019	37	−2.10	6.95	37	−1.96	5.41	−0.02	[−0.48; 0.43]	8.1%
Whittingham et al., 2022	37	0.11	2.39	30	0.69	2.65	−0.23	[−0.71; 0.25]	8.0%
Chong et al., 2019	84	−2.70	4.26	84	−1.53	4.94	−0.25	[−0.56; 0.05]	8.3%
Han et al., 2023	9	−2.11	3.31	10	−0.90	2.82	−0.38	[−1.29; 0.53]	6.9%
Brown et al., 2015	30	−3.59	5.08	29	0.09	6.88	−0.60	[−1.12; −0.08]	7.9%
Whittingham et al., 2016	23	−1.69	3.60	22	1.48	5.29	−0.69	[−1.29; −0.09]	7.8%
Losada et al., 2015	33	−8.12	5.74	31	−2.29	5.72	−1.01	[−1.53; −0.48]	7.9%
Yuan, 2021	35	−8.23	6.72	35	0.23	6.00	−1.31	[−1.83; −0.79]	7.9%
Marquez–Gonzalez et al., 2020	16	−8.66	5.45	9	−0.34	5.77	−1.45	[−2.37; −0.52]	6.9%
Wang, 2020	72	−9.09	4.65	72	−0.41	5.19	−1.75	[−2.14; −1.37]	8.2%
Çiçek Gümü.. & Öncel, 2022	30	−10.20	3.89	30	−0.14	3.30	−2.75	[−3.47; −2.03]	7.5%
Hadavand et al., 2022	20	−7.00	2.56	20	2.90	3.17	−3.37	[−4.36; −2.38]	6.7%
**Random effects model**	**451**			**434**			**−1.02**	**[−1.57; −0.48]**	**100.0%**
**Prediction interval**	.			.				**[−3.21; 1.16]**	**--**
Heterogeneity: $I^2 = 90\%$ [85%; 94%], $\chi^2_{12} = 123.18$ ($p < 0.001$)									
**4– to 6–month follow–up**									
Losada et al., 2015	25	−6.93	5.65	22	−3.26	5.64	−0.64	[−1.23; −0.05]	20.6%
Chong et al., 2019	84	−2.13	4.27	84	1.03	4.81	−0.69	[−1.00; −0.38]	22.1%
Sairanen et al., 2019	37	−3.40	7.11	37	3.32	11.33	−0.70	[−1.17; −0.23]	21.3%
Marquez–Gonzalez et al., 2020	7	−5.46	5.83	15	0.25	5.80	−0.95	[−1.89; 0.00]	18.0%
Hadavand et al., 2022	20	−5.20	2.53	20	4.00	3.10	−3.18	[−4.14; −2.22]	17.9%
**Random effects model**	**173**			**178**			**−1.18**	**[−2.07; −0.28]**	**100.0%**
**Prediction interval**	.			.				**[−4.55; 2.19]**	**--**
Heterogeneity: $I^2 = 84\%$ [63%; 93%], $\chi^2_4 = 24.59$ ($p < 0.001$)									
**1– to 3–month follow–up**									
Han et al., 2023	9	−0.44	4.33	10	−0.90	2.82	0.12	[−0.78; 1.02]	18.0%
Mosher et al., 2019	25	−0.68	2.60	25	−0.87	2.67	0.07	[−0.48; 0.63]	20.4%
X.X. Xu et al., 2020	41	−5.16	5.15	43	−1.92	4.75	−0.65	[−1.09; −0.21]	21.0%
Wang, 2020	72	−9.42	4.60	72	−0.73	5.13	−1.77	[−2.16; −1.39]	21.3%
Çiçek Gümü.. & Öncel, 2022	30	−9.04	3.78	30	0.46	3.06	−2.73	[−3.44; −2.01]	19.3%
**Random effects model**	**177**			**180**			**−1.00**	**[−2.07; 0.06]**	**100.0%**
**Prediction interval**	.			.				**[−5.12; 3.12]**	**--**
Heterogeneity: $I^2 = 93\%$ [87%; 96%], $\chi^2_4 = 59.85$ ($p < 0.001$)									

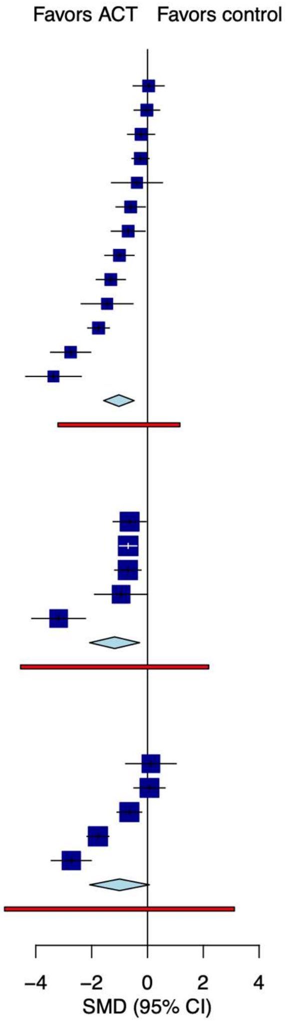

Heterogeneity: $I^2 = 90\%$ [86%; 92%], $\chi^2_{22} = 212.52$ ($p < 0.001$)
Test for subgroup differences: $p = 0.954$

eFigure 9. Forest Plots for Stress Symptoms at Postintervention and 1- to 3-Month and 4- to 6-Month Follow-Ups

Study	Intervention group Total	Intervention group Mean	Intervention group SD	Control group Total	Control group Mean	Control group SD	SMD	95%-CI	Weight
**Post–intervention**									
Davis et al., 2020	35	−0.29	6.44	20	−2.95	7.36	0.39	[−0.17; 0.94]	5.1%
Whittingham et al., 2022	37	0.15	3.32	30	−0.30	3.54	0.13	[−0.35; 0.61]	5.2%
Mosher et al., 2022	20	−1.93	2.97	20	−2.08	6.19	0.03	[−0.59; 0.65]	5.1%
Jolley et al., 2020	16	−1.80	5.30	10	−1.40	6.32	−0.07	[−0.86; 0.72]	4.8%
Jin et al., 2022	20	−11.28	16.46	20	−9.36	23.87	−0.09	[−0.71; 0.53]	5.1%
Han et al., 2023	9	−3.78	2.11	10	−3.40	3.53	−0.12	[−1.02; 0.78]	4.7%
Sairanen et al., 2019	37	−3.70	8.26	37	−2.57	7.18	−0.14	[−0.60; 0.31]	5.3%
De Wit et al., 2020	74	−0.63	5.73	74	0.53	5.79	−0.20	[−0.52; 0.12]	5.4%
Köhle et al., 2021 (Online + AF)	70	0.40	5.41	33	2.00	4.78	−0.30	[−0.72; 0.11]	5.3%
Potter et al., 2021 (Email)	9	0.86	7.79	4	4.87	12.32	−0.40	[−1.60; 0.79]	4.2%
Köhle et al., 2021 (Online + PF)	67	−0.10	4.82	33	2.00	4.78	−0.43	[−0.85; −0.01]	5.3%
Xiao, 2018	34	−3.26	6.51	36	−0.84	3.47	−0.46	[−0.94; 0.01]	5.2%
Lappalainen et al., 2021	40	−11.82	11.12	42	−5.83	11.85	−0.52	[−0.96; −0.08]	5.3%
Chong et al., 2019	84	−2.29	6.34	84	1.25	7.12	−0.52	[−0.83; −0.22]	5.4%
Mosher et al., 2019	25	−0.29	1.87	25	0.94	1.92	−0.64	[−1.21; −0.07]	5.1%
Whittingham et al., 2016	23	−3.91	4.94	22	0.05	7.03	−0.64	[−1.24; −0.04]	5.1%
Brown et al., 2015	30	−4.57	6.24	29	−0.04	6.89	−0.68	[−1.21; −0.15]	5.2%
Potter et al., 2021 (Email + phone)	7	−6.20	13.02	4	4.87	12.32	−0.79	[−2.08; 0.50]	4.0%
Marino et al., 2021	20	−6.90	6.12	20	1.80	7.19	−1.28	[−1.96; −0.59]	5.0%
Çiçek Gümü.. & Öncel, 2022	30	−12.80	2.56	30	−0.90	1.96	−5.15	[−6.23; −4.08]	4.4%
**Random effects model**	**687**			**583**			**−0.55**	**[−0.98; −0.12]**	**100.0%**
**Prediction interval**	.			.				**[−2.54; 1.44]**	**--**
Heterogeneity: $I^2 = 82\%$ [73%; 88%], $\chi^2_{19} = 103.73$ ($p < 0.001$)									
**1– to 3–month follow–up**									
Han et al., 2023	9	−2.67	2.98	10	−3.70	3.81	0.29	[−0.62; 1.19]	16.0%
Mosher et al., 2022	20	−0.53	3.33	20	−0.28	6.54	−0.05	[−0.67; 0.57]	17.4%
Mosher et al., 2019	25	−0.69	1.87	25	−0.41	1.92	−0.15	[−0.70; 0.41]	17.7%
Jolley et al., 2020	17	−3.40	4.74	12	−0.70	5.84	−0.50	[−1.25; 0.25]	16.8%
Xiao, 2018	34	−7.41	6.95	36	−1.56	3.45	−1.06	[−1.57; −0.56]	17.9%
Çiçek Gümü.. & Öncel, 2022	30	−13.17	2.49	30	−0.25	1.86	−5.80	[−6.98; −4.61]	14.4%
**Random effects model**	**135**			**133**			**−1.18**	**[−2.95; 0.60]**	**100.0%**
**Prediction interval**	.			.				**[−7.74; 5.39]**	**--**
Heterogeneity: $I^2 = 94\%$ [90%; 97%], $\chi^2_5 = 86.02$ ($p < 0.001$)									
**4– to 6–month follow–up**									
De Wit et al., 2020	74	−0.38	5.77	74	0.64	5.66	−0.18	[−0.50; 0.15]	15.6%
Davis et al., 2020	35	−5.35	5.84	20	−3.23	7.84	−0.32	[−0.87; 0.24]	14.9%
Köhle et al., 2021 (Online + PF vs. Online +AF)	67	−0.70	5.29	70	1.10	5.41	−0.33	[−0.67; 0.00]	15.6%
Chong et al., 2019	84	−2.99	6.23	84	0.68	6.36	−0.58	[−0.89; −0.27]	15.6%
Sairanen et al., 2019	37	−7.05	7.97	37	0.27	8.89	−0.86	[−1.34; −0.38]	15.2%
Potter et al., 2021 (Email)	9	−4.86	8.78	4	5.62	11.06	−1.03	[−2.30; 0.24]	11.8%
Potter et al., 2021 (Email + phone)	7	−11.60	14.77	4	5.62	11.06	−1.15	[−2.52; 0.21]	11.3%
**Random effects model**	**313**			**293**			**−0.47**	**[−0.69; −0.25]**	**100.0%**
**Prediction interval**	.			.				**[−0.98; 0.04]**	**--**
Heterogeneity: $I^2 = 31\%$ [0%; 71%], $\chi^2_6 = 8.73$ ($p = 0.189$)									

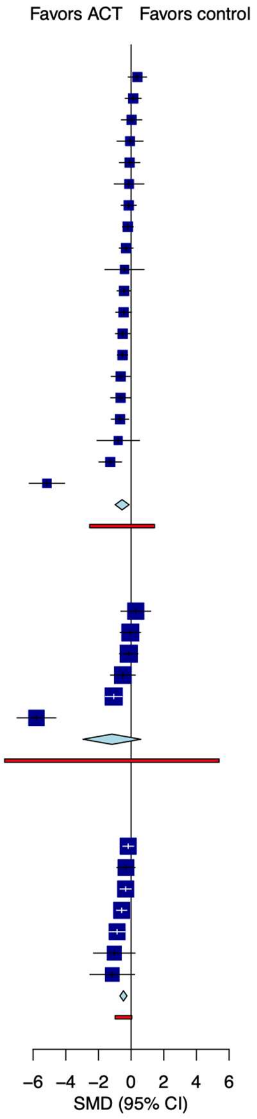

Heterogeneity: $I^2 = 84\%$ [79%; 88%], $\chi^2_{32} = 202.61$ ($p < 0.001$)
Test for subgroup differences: $p = 0.711$

eFigure 10. Funnel Plots Depicting Standard Error of Effect Sizes for Psychological Flexibility

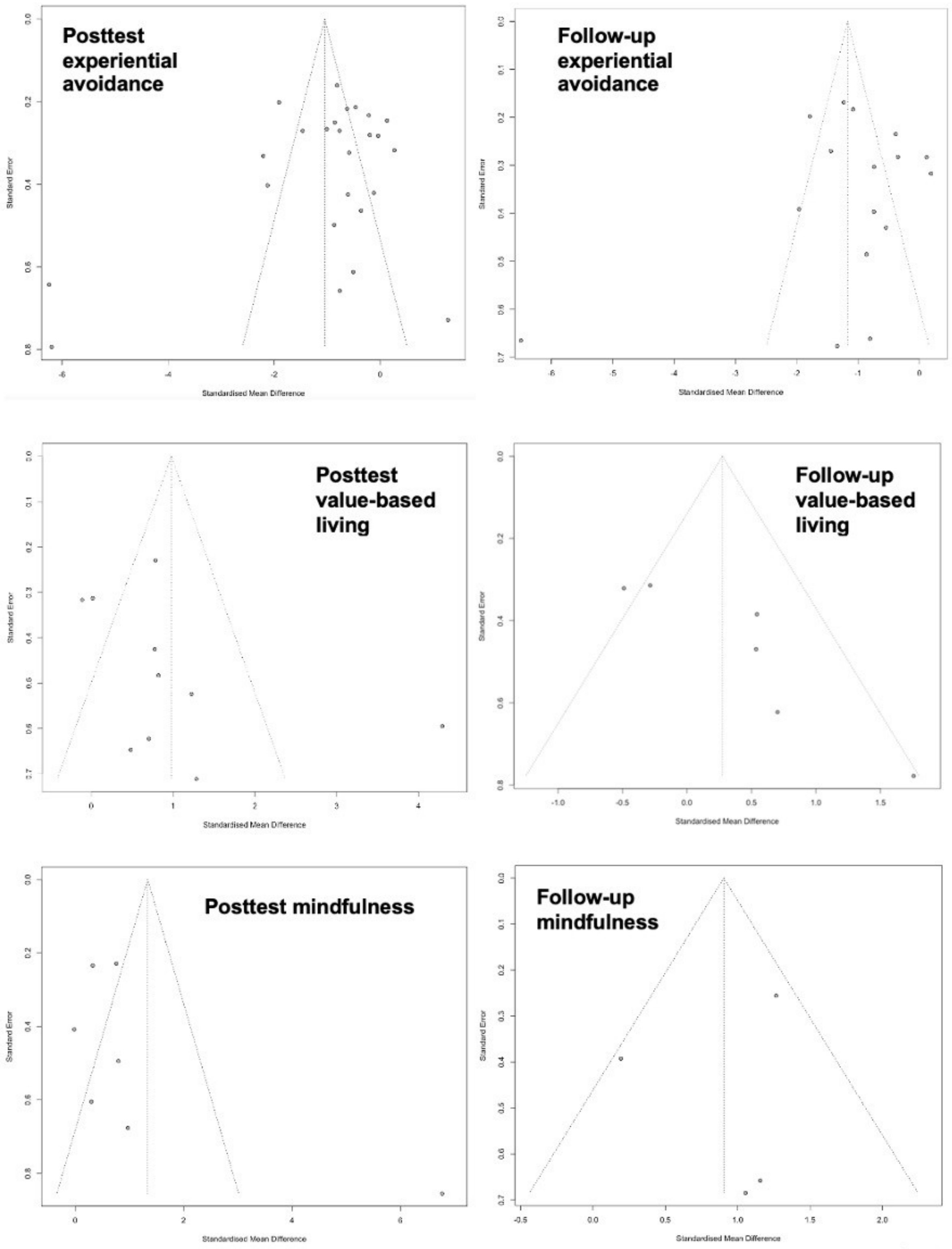

(continued)

eFigure 10. Funnel Plots Depicting Standard Error of Effect Sizes for Psychological Flexibility (continued)

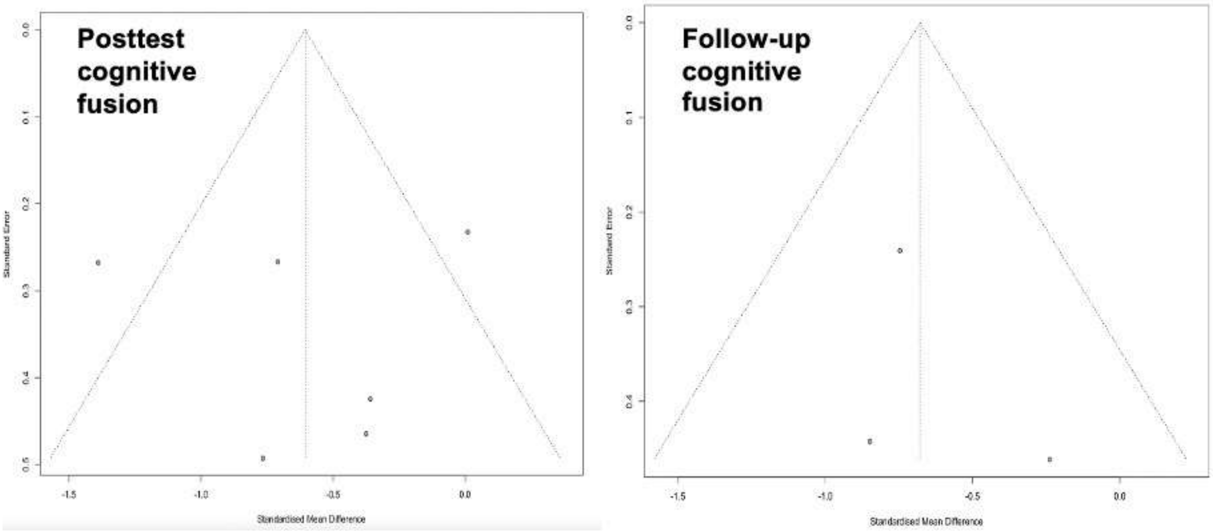

eFigure 11. Funnel Plots Depicting Standard Error of Effect Sizes for Other Psychological Health Outcomes

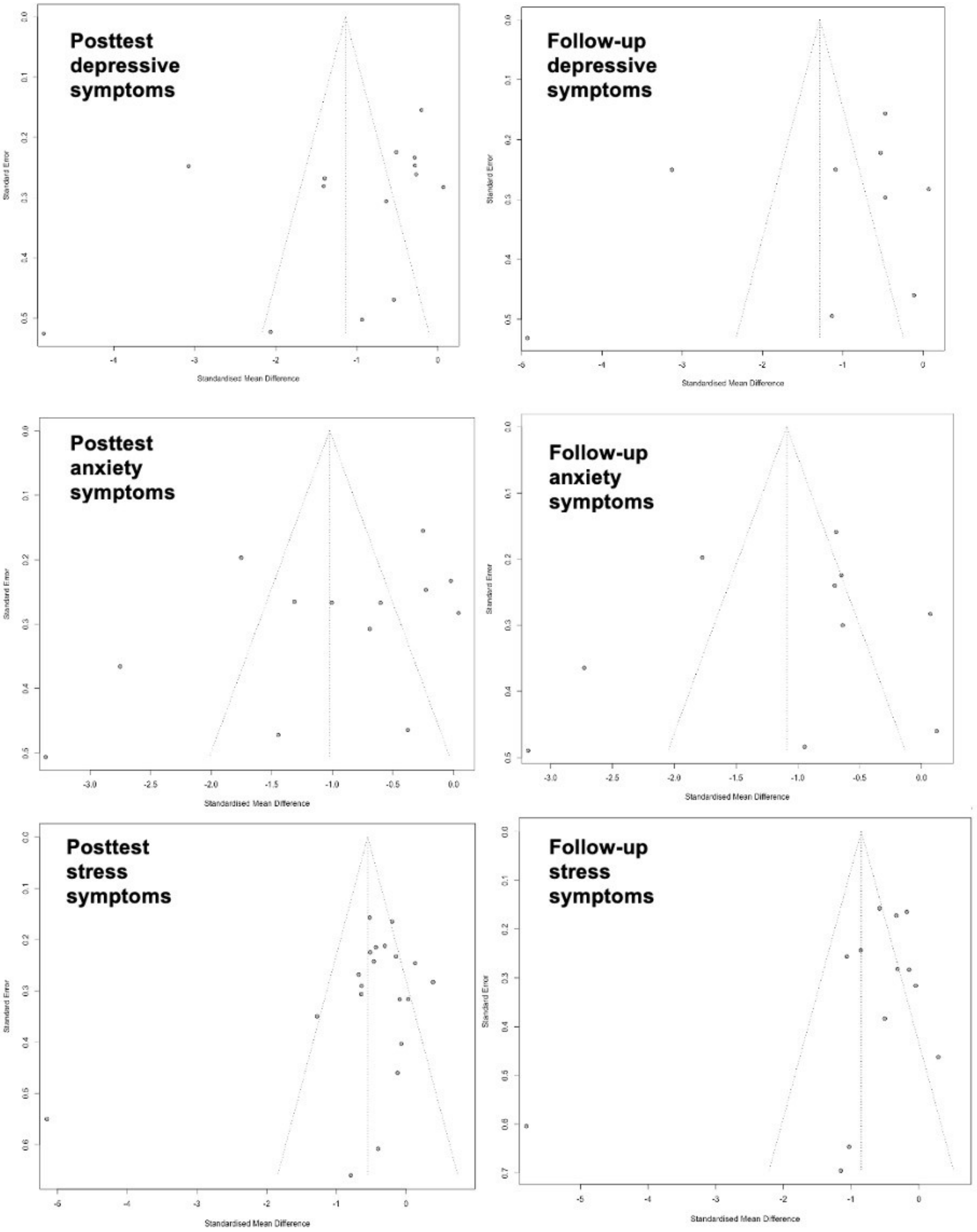

eReferences

1. The Cochrane Developmental, Psychosocial and Learning Problems Review Group. Data collection form for intervention reviews: RCTs only. Accessed May 1, 2023. https://dplp.cochrane.org/data-extraction-forms
2. Higgins J, Thomas J, Chandler J, et al, eds. *Cochrane Handbook for Systematic Reviews of Interventions, Version 6.2 (Updated February 2021).* Accessed May 1, 2023. https://training.cochrane.org/handbook
3. Rosenthal R. *Meta-Analytic Procedures for Social Research*. Accessed May 1, 2023. https://methods.sagepub.com/book/meta-analytic-procedures-for-social-research
4. Collins RN, Kishita N. The effectiveness of mindfulness- and acceptance-based interventions for informal caregivers of people with dementia: A meta-analysis. *Grerontol*. 2019;59(4):E363-E379. doi:10.1093/geront/gny024
5. Fritz CO, Morris PE, Richler JJ. Effect size estimates: current use, calculations, and interpretation. *J Exp Psychol Gen*. 2012;141(1):2. doi:10.1037/a0024338
6. Hedges LV. Distribution theory for Glass's estimator of effect size and related estimators. *J Educ Stat*. 1981;6(2):107-128. doi:10.2307/1164588
7. Borenstein M, Hedges LV, Higgins JP, Rothstein HR. *Introduction to meta-analysis*. John Wiley & Sons; 2021.
8. Viechtbauer W. Bias and efficiency of meta-analytic variance estimators in the random-effects model. *J Educ Behav Stat*. 2005;30(3):261-293. doi:10.3102/10769986030003261
9. Davis EL, Deane FP, Lyons GC, Barclay GD, Bourne J, Connolly V. Feasibility randomized controlled trial of a self-help acceptance and commitment therapy intervention for grief and psychological distress in carers of palliative care patients. *J Health Psychol*. 2020;25(3):322-339. doi:10.1177/1359105317715091
10. Whittingham K, Sanders MR, McKinlay L, Boyd RN. Parenting intervention combined with acceptance and commitment therapy: A trial with families of children with cerebral palsy. *J Pediatr Psychol*. 2016;41(5):531-42. doi:10.1093/jpepsy/jsv118
11. Brown FL, Whittingham K, Boyd RN, McKinlay L, Sofronoff K. Does Stepping Stones Triple P plus Acceptance and Commitment Therapy improve parent, couple, and family adjustment following paediatric acquired brain injury? A randomised controlled trial. *Behav Res Ther*. 2015;73:58-66. doi:10.1016/j.brat.2015.07.001
12. Whittingham K, Sheffield J, Mak C, Wright A, Boyd RN. Parenting Acceptance and Commitment Therapy: an RCT of an online course with families of children with CP. *Behav Res Ther*. 2022;155:104129. doi:10.1016/j.brat.2022.104129
13. Drouillard BE. *Supporting treatment selection in parents of children with autism spectrum disorder: An educational workshop with acceptance and commitment training*. Dissertation. University of Windsor (Canada); 2020. Accessed May 1, 2023. https://www.proquest.com/docview/2322824738?pq-origsite=gscholar&fromopenview=true
14. Yuan B. *A study on psychological intervention of acceptance and commitment therapy for parents of children with autism at first diagnosis.* Master's thesis. Nanchang University (China); 2021. Accessed May 1, 2023. https://d.wanfangdata.com.cn/thesis/ChJUaGVzaXNOZXdTMjAyMzAxMTISCUQwMjQzNjk2NBoIOGN3YXN3NGM%3D
15. Zhiping W. *Application study of acceptance and commitment therapy in psychological intervention of main caregivers for children with cerebral palsy.* Master's thesis. Anhui Medical University (China); 2020. Accessed May 1, 2023. https://d.wanfangdata.com.cn/thesis/ChJUaGVzaXNOZXdTMjAyMzAxMTISCUQwMjI3OTU0ORoIZXB5M25kazk%3D
16. Jing X. *Study of acceptance and commitment therapy in family function of the first episode schizophrenia patients*. Master's thesis. Nanchang University (China); 2020. Accessed May 1, 2023. https://d.wanfangdata.com.cn/thesis/ChJUaGVzaXNOZXdTMjAyMzAxMTISCUQwMjIwNDM4NxoIejVseXhxa3c%3D
17. Chong YY, Mak YW, Leung SP, Lam SY, Loke AY. Acceptance and Commitment Therapy for Parental Management of Childhood Asthma: An RCT. *Pediatrics*. 2019;143(2):e20181723. doi:10.1542/peds.2018-1723
18. Na x. *A study on intervention of acceptance and commitment therapy in psychological of parents of children with leukemia*. Master's thesis. University of South China (China); 2018.

https://d.wanfangdata.com.cn/thesis/ChJUaGVzaXNOZXdTMjAyMzAxMTISCUQwMTY4ODQwNhoIM3BpOHhobDU%3D
19. Jin X, Li H, Chong YY, Mann KF, Yao W, Wong CL. Feasibility and preliminary effects of acceptance and commitment therapy on reducing psychological distress and improving the quality of life of the parents of children with cancer: A pilot randomized controlled trial. *Psycho-Oncol*. 2023;32(1):165-169. doi:10.1002/pon.5941
20. Xiuxian X, Haiying H, Zhimin L. Effects of acceptance and commitment therapy on anxiety, depression, and posttraumatic growth of parents of children with leukemia. *Chinese Gen Pract Nurs*. 2020;18(25):3318-3320. doi:10.12104/j.issn.1674-4748.2020.25.015
21. Lappalainen P, Pakkala I, Strömmer J, Sairanen E, Kaipainen K, Lappalainen R. Supporting parents of children with chronic conditions: A randomized controlled trial of web-based and self-help ACT interventions. *Internet Interv*. 2021;24:100382. doi:10.1016/j.invent.2021.100382
22. Marino F, Failla C, Chilà P, et al. The Effect of Acceptance and Commitment Therapy for Improving Psychological Well-Being in Parents of Individuals with Autism Spectrum Disorders: A Randomized Controlled Trial. *Brain Sci*. 2021;11(7): 880. doi:10.3390/brainsci11070880
23. Köhle N, Drossaert CHC, Ten Klooster PM, et al. Web-based self-help intervention for partners of cancer patients based on acceptance and commitment therapy and self-compassion training: a randomized controlled trial with automated versus personal feedback. *Support Care Cancer*. 2021;29(9):5115-5125. doi:10.1007/s00520-021-06051-w
24. De Wit J, Beelen A, Drossaert CHC, et al. Blended psychosocial support for partners of patients with ALS and PMA: results of a randomized controlled trial. *Amyotroph Lateral Scher Frontotemp Degener*. 2020;21(5-6):344-354. doi:10.1080/21678421.2020.1757114
25. Marquez-Gonzalez M, Romero-Moreno R, Cabrera I, Olmos R, Perez-Miguel A, Losada A. Tailored versus manualized interventions for dementia caregivers: The functional analysis-guided modular intervention. *Psychol Aging*. 2020;35(1):41-54. doi:10.1037/pag0000412
26. Losada A, Márquez-González M, Romero-Moreno R, et al. Cognitive–behavioral therapy (CBT) versus acceptance and commitment therapy (ACT) for dementia family caregivers with significant depressive symptoms: Results of a randomized clinical trial. *J Consult Clin Psychol*. 2015;83(4):760-772. doi:10.1037/ccp0000028
27. Sairanen E, Lappalainen R, Lappalainen P, et al. Effectiveness of a web-based acceptance and commitment therapy intervention for wellbeing of parents whose children have chronic conditions: A randomized controlled trial. *J Contextual Behav Sci.* 2019; 13: 94-102. doi:10.1016/j.jcbs.2019.07.004
28. Kanstrup M, Wicksell RK, Kemani M, Wiwe Lipsker C, Lekander M, Holmström L. A clinical pilot study of individual and group treatment for adolescents with chronic pain and their parents: Effects of acceptance and commitment therapy on functioning. *Children (Basel)*. 2016;3(4):30. doi:10.3390/children3040030
29. Çiçek Gümüş E, Öncel S. Effects of acceptance and commitment therapy-based interventions on the mental states of parents with special needs children: Randomized controlled trial. *Curr Psychol*. 2022:1-14. doi:10.1007/s12144-022-03760-1
30. Hadavand M, Zanjani Z, Omidi A, Atoof F, Fakharian E. Acceptance and commitment: An intervention for improving family function and emotional problems in informal caregivers of people with severe traumatic brain injury: A randomized clinical trial. *Arch Trauma Res*. 2022;11(2):90-96. doi:10.4103/atr.atr_4_22
31. Potter KJ, Golijana-Moghaddam N, Evangelou N, Mhizha-Murira JR, das Nair R. Self-help acceptance and commitment therapy for carers of people with multiple sclerosis: A feasibility randomized controlled trial. *J Clin Psychol Med Settings*. 2021;28(2):279-294. doi:10.1007/s10880-020-09711-x
32. Jolley S, Johns LC, O'Donoghue E, et al. Group acceptance and commitment therapy for patients and caregivers in psychosis services: Feasibility of training and a preliminary randomized controlled evaluation. *Br J Clin Psychol*. 2020;59(4):524-551. doi:10.1111/bjc.12265
33. Lloyd A. *The use of Acceptance and Commitment Therapy to address psychological distress experienced by caregivers: a randomized controlled feasibility trial*. Dissertation. University of Glasgow (United Kingdom); 2016. https://theses.gla.ac.uk/7590/1/2016LloydDClinPsy.pdf
34. Mosher CE, Secinti E, Wu W, et al. Acceptance and commitment therapy for patient fatigue interference and caregiver burden in advanced gastrointestinal cancer: Results of a pilot randomized trial. *Palliat Med*. 2022;36(7):1104-1117. doi:10.1177/02692163221099610
35. Hahs AD, Dixon MR, Paliliunas D. Randomized controlled trial of a brief acceptance and commitment training for parents of individuals diagnosed with autism spectrum disorders. *J Contextual Behav Sci*. 2019;12:154-159. doi:10.1016/j.jcbs.2018.03.002

36. Mosher CE, Secinti E, Hirsh AT, et al. Acceptance and Commitment Therapy for Symptom Interference in Advanced Lung Cancer and Caregiver Distress: A Pilot Randomized Trial. *J Pain Symptom Manage*. 2019;58(4):632-644. doi:10.1016/j.jpainsymman.2019.06.021
37. Han A, Yuen HK, Jenkins J. The feasibility and preliminary effects of a pilot randomized controlled trial: Videoconferencing acceptance and commitment therapy in distressed family caregivers of people with dementia. *J Health Psychol*. 2023;28(6):554-567. doi:10.1177/13591053221141131
38. Hayes SC. *Get out of your mind and into your life: The new acceptance and commitment therapy*. New Harbinger Publications; 2005.
39. Harris R. *ACT made simple: An easy-to-read primer on acceptance and commitment therapy*. New Harbinger Publications; 2019.
