# Supplementary material for: Acceptance and Commitment Therapy Among Informal Caregivers of People With Chronic Health Conditions: A Systematic Review and Meta-Analysis
Source: JAMA Netw Open. 2023 Dec 5;6(12):e2346216. doi: 10.1001/jamanetworkopen.2023.46216 (PMC10698615; doi:10.1001/jamanetworkopen.2023.46216)
Supplement: Supplement 2. — Data Sharing Statement [file jamanetwopen-e2346216-s002.pdf]

Data Sharing Statement

Ye. Acceptance and Commitment Therapy Among Informal Caregivers of People With Chronic Health Conditions. *JAMA Netw Open*. Published December 05, 2023. doi:10.1001/jamanetworkopen.2023.46216

Data
Data available: No

Additional Information
Explanation for why data not available: Data for this review was derived from published randomized clinical trials and data analysis code is available on request from the corresponding author.
